# A Resistome Roadmap: From the Human Body to Pristine Environments

**DOI:** 10.3389/fmicb.2022.858831

**Published:** 2022-05-11

**Authors:** Lucia Maestre-Carballa, Vicente Navarro-López, Manuel Martinez-Garcia

**Affiliations:** ^1^Department of Physiology, Genetics and Microbiology, University of Alicante, Alicante, Spain; ^2^Clinical Microbiology and Infectious Disease Unit, Hospital Universitario Vinalopó, Elche, Spain

**Keywords:** antibiotic resistance genes, human microbiome project, pristine environments, antibiotic resistance, metagenomics

## Abstract

A comprehensive characterization of the human body resistome [sets of antibiotic resistance genes (ARGs)] is yet to be done and paramount for addressing the antibiotic microbial resistance threat. Here, we study the resistome of 771 samples from five major body parts (skin, nares, vagina, gut, and oral cavity) of healthy subjects from the Human Microbiome Project (HMP) and addressed the potential dispersion of ARGs in pristine environments. A total of 28,714 ARGs belonging to 235 different ARG types were found in the HMP proteome dataset (*n* = 9.1 × 10^7^ proteins analyzed). Our study reveals a distinct resistome profile (ARG type and abundance) between body sites and high interindividual variability. Nares had the highest ARG load (≈5.4 genes/genome) followed by the oral cavity, whereas the gut showed one of the highest ARG richness (shared with nares) but the lowest abundance (≈1.3 genes/genome). The fluroquinolone resistance genes were the most abundant in the human body, followed by macrolide–lincosamide–streptogramin (MLS) or tetracycline. Most ARGs belonged to common bacterial commensals and multidrug resistance trait were predominant in the nares and vagina. Many ARGs detected here were considered as low risk for human health, whereas only a few of them, such as *BlaZ*, *dfrA*14, *dfrA*17, or *tetM*, were classified as high-risk ARG. Our data also provide hope, since the spread of common ARG from the human body to pristine environments (*n* = 271 samples; 77 Gb of sequencing data and 2.1 × 10^8^ proteins analyzed) thus far remains very unlikely (only one case found in an autochthonous bacterium from a pristine environment). These findings broaden our understanding of ARG in the context of the human microbiome and the One-Health Initiative of WHO uniting human host–microbes and environments as a whole.

## Introduction

Since the discovery of antibiotics, human health and animal health have profoundly changed. Undoubtedly, antibiotics have not only saved millions of lives but also have transformed modern medicine (Ventola, 2015; Centers for Disease Control and Prevention, 2019). Prior to the antimicrobial agents’ era, the process of reduction in the prevalence of infectious diseases begun much earlier with the introduction of nutritional, environmental, and behavioral changes in our society (McKeown, 1980). Nevertheless, the use of antibiotics has substantially improved our health, but now unfortunately, an overuse, incorrect prescription, extensive use of antibiotics in agriculture and farming and the low availability of new antibiotics have led to a major antibiotic resistance crisis, wherein bacterial pathogens are becoming resistant to available antibiotics (Ventola, 2015). In the US, it has been estimated that antibiotic-resistant organisms cause 2.8 million infections and 35,900 deaths each year (Centers for Disease Control and Prevention, 2019). It is worth noting that the estimation of deaths due to antibiotic resistance is very challenging, and therefore, those number should be handled with care as recently discussed (Baquero, 2021). This is not only a global health issue but also affects food security and requires significant financial investment. For instance, it has been estimated that in 2017, the annual treatment of six multidrug-resistant bacteria costs approximately $4.6 billion to the US healthcare system (Nelson et al., 2021). By 2050, the predictions estimate that over 10 million deaths and a total cost of ≈100 trillion USD will be attributed to antibiotic resistance worldwide (Brogan and Mossialos, 2016; O’Neill, 2016), and recently, the WHO estimated that in 10 years, antimicrobial resistance could force up to 24 million people into extreme poverty (IACG, 2019).

Antibiotic resistance is a natural process in which bacteria become resistant to antibiotics using different mechanisms, which are classified as phenotypic resistance (due to physiological changes and non-hereditary) or acquired (when antibiotic resistance is genetically gained) (Olivares et al., 2013). Different antibiotic resistance genes (ARGs) that confer resistance to antibiotics could be acquired due to mutations in the bacterial genome (Sun et al., 2014) or through horizontal gene transfer (HGT). The transfer of ARGs could be mediated by bacteria, viruses, plasmids, or even vesicles (Emamalipour et al., 2020). Among the possible antibiotic classifications, the most common are the ones based on their chemical structure (drug classes, e.g., tetracycline, beta-lactams, and aminoglycosides), mode of action (determined by the antibiotic target, mainly proteins, cell membrane, and nucleic acids), and spectrum of activity (from narrow to broad) (Wright, 2010; Etebu and Arikekpar, 2016; Reygaert, 2018).

The occurrence of antibiotic resistance has increased and accelerated since antibiotics are constantly present in the environment, derived from anthropogenic sources such as wastewater treatment plants, hospitals, or domestic use (Rodriguez-Mozaz et al., 2020). Another cause of this increase is the dispersion of resistant bacteria from hot spots, such as wastewater treatment plants (from our own human microbiome) and built environments (i.e., microorganisms found in human-constructed environments) (Baron et al., 2018; Centers for Disease Control and Prevention, 2019), which continuously disseminates our microbes and thus parts of their genetic content.

Although ARGs are present in many environments, even in remote ones (Van Goethem et al., 2018; Naidoo et al., 2020), not all ARGs pose the same risk for human health, and thus, the detection of these genes should be interpreted with caution (Martínez et al., 2014). For instance, efflux pumps are widely distributed in bacteria, and they are involved in many different functions, such as cell homeostasis. Additionally, efflux pumps have been reported as one of the mechanisms responsible for antimicrobial resistance, and therefore, they should not be considered as sensu stricto ARG since their original function was not to provide antibiotic resistance.

The human microbiome project (HMP) (Methé et al., 2012), an interdisciplinary effort, was developed with the objective of characterizing the human microbiome. For this purpose, samples from different major body parts of healthy humans were obtained and sequenced, which produces one of the largest resources for the study of the human microbiome (Huttenhower et al., 2012). To the best of our knowledge, comprehensive analysis and cross-comparison of the human resistome from all human body parts studied within the HMP have not been performed, but to date, some valuable but separate and non-interconnected studies have been performed for the oral cavity and the skin (Carr et al., 2020; Li et al., 2021). Addressing the abundance and diversity of ARGs as a whole in all human body parts has enormous potential to broaden our knowledge on the dispersion of ARGs from human bacteria within different microbial populations in nature.

Thus, here, in the context of antibiotic resistance-related health concerns, in addition to analyzing in detail the ARGs present in the HMP-studied body sites, we studied the potential spread of ARGs from the human body to different types of pristine environments (polar, desert, cave, hot spring, and submarine volcano environments). These environments are supposed to be undisturbed and not affected by anthropic actions. While many places, such as caves or polar environments with no apparent and visible human activity, are often perceived as pristine environments, human activity unfortunately leaves an indirect ever-increasing footprint. Here, we use some of these pristine environments as a model to address and estimate the potential “mobility” of common human ARGs found in the human body to better assess the global impact of antibiotic resistance in our ecosystems, in line with the One Health concept (i.e., human health and animal health are interdependent and bound to ecosystems) (Atlas, 2012; Hernando-Amado et al., 2019). Pristine environments are commonly used as “reporter ecosystems” to monitor pollution and climate change and, in our case, specifically to measure how deep the potential impact of the spread of antibiotic resistance is.

## Materials and Methods

### Sample Collection

A total of 751 shotgun-sequenced samples from 15 different parts of the body from healthy American adults belonging to the HMP (Huttenhower et al., 2012) were retrieved from JGI-IMG/ER (Chen et al., 2021; Supplementary Table 1). Not all HMP-assembled data present at JGI-IMG/ER were accessible, thus only the available metagenomes were included in this study. The data were organized in 5 groups: skin (retro-auricular crease), nares, gut, vagina (posterior-fornix, mid vagina, and vagina introitus), and oral cavity (hard palate, buccal mucosa, saliva, subgingival plaque, attached gingivae, tongue dorsum, throat, palatine tonsils, and supragingival plaque).

A total of 20 metagenomes belonging to left and right retro-auricular crease that could not be found in JGI-IMG were downloaded from the HMP page^1^ and included with the rest of HMP samples (Supplementary Table 1).

Proteins of 271 metagenomes from pristine environments (or environments with no or little human presence) were downloaded from JGI-IMG, and they were organized in 5 groups: arid deserts (65), submarine volcanoes (66), hot springs (68), polar environments (57), and caves (15), which yields a total of 76 Gb (Supplementary Table 2). Environments associated with a host (e.g., tubeworms) were also discarded.

### Human Microbiome Project Resistome *in silico* Analysis

Proteins from 751 samples of the HMP were retrieved from de JGI-IMG/ER (Chen et al., 2021). In addition, 20 assembled metagenomes were downloaded directly from the HMP official page since they were not available in JGI. Open reading frames (ORF) of the genomic sequences downloaded from HMP were predicted with prodigal 2.6.3 (Hyatt et al., 2010).

Then, all obtained proteins were compared using BLASTp 2.8.1 + with the following antibiotic resistance protein databases: CARD 3.0.3 (Jia et al., 2017), ARG-ANNOT (Gupta et al., 2014), and RESFAMS (Gibson et al., 2015). Aiming to identify only high-confidence ARG, only those ARGs with *e*-value ≤ 10^–5^, amino acid identity ≥ 90%, and bit-score ≥ 70 with the mentioned ARG databases were considered as hits, thus being more conservative than other accepted thresholds (bit-score ≥ 70) (Enault et al., 2017). Housekeeping genes with mutations confering resistance to antibiotic were ruled out from our final results.

Best-hit ARGs found in HMP after comparing proteins with the above-mentioned ARG databases were grouped using their gene name (or synonyms) and, in addition, grouped according to the drug class they confer resistance to, following CARD 3.0.3 annotation (Jia et al., 2017) and being manually curated. Best-hits ARGs not appearing in CARD database were compared with NCBI nr database (2021) using BLASTp. Each ARG found in HMP was associated with a scaffold. The taxonomic affiliation for resistant bacteria was extracted from the scaffold annotation found in JGI/IMG-ER (Chen et al., 2021), which was done using the annotation pipeline MAP v.4.10 (Huntemann et al., 2016). The contig’s phylogeny was obtained establishing for each protein the taxonomical best-hit using USEARCH (Edgar and Bateman, 2010) against the IMG-NR database and determined the contig phylogeny based on a majority-based rule where the last common ancestor of the proteins was assigned (when at least 30% of the genes had a hit with USEARCH). To compare the obtained results, hits were normalized by the assembled mega-base pair (Mb).

Multiresistant contigs were manually curated, and only those with at least 2 different ARGs conferring resistance to at least two different drug classes were included in the analysis. The abundance of metagenomes with multiresistant contigs was calculated by dividing the number of metagenomes with at least one multiresistant contig by the total number of metagenomes studied. The frequency of multiresistant species only in metagenomes with more than one multiresistant bacteria was done by dividing the number of multiresistant by the total number of contigs.

The presence of common ARGs in all the analyzed parts of the body was performed using CD-HIT (90% identity) (Fu et al., 2012), which was used to cluster all the ARGs found in the HMP.

For studying the effect of our threshold in housekeeping genes that require few mutations to become resistant, gyrA genes from the HMP dataset that were considered *a priori* as ARG using our threshold were extracted and aligned against the gyrA fluroquinolone resistant gene deposited in CARD (Jia et al., 2017) from *Mycobacterium tuberculosis* [>gb|CCP42728.1|+|Mycobacterium tuberculosis gyrA conferring resistance to fluoroquinolones (Mycobacterium tuberculosis H37Rv)] and gyrA^R^ obtained from RESFAMS (Gibson et al., 2015) (NC_002952_2859949_p01) from *Staphylococcus*. The alignment was performed with MUSCLE available in Geneious 9.1.3.

### Human Microbiome Project Antibiotic Resistance Genes in Pristine Environments

To determine the presence of ARG from the HMP in pristine environments with presumptive low or none human presence, the ARGs obtained from the human samples were compared with the proteins from the chosen metagenomes using BLASTp 2.8.1+. Only those hits with an amino acid identity ≥ 90%, a bit-score ≥ 70, and *e*-value ≤ 10^–5^ were considered. The taxonomic annotation was retrieved from JGI-IMG/ER only for those scaffolds with at least 4 proteins to ensure the detection of HMP ARGs in autochthonous bacteria (3 out of, at least, 4 proteins should be from the autochthonous bacteria). All the hits were manually curated to avoid false positives, especially those produced by housekeeping genes. Those belonging to taxons that could not be associated with a specific environment were discarded.

Alignments were performed using the software Geneious 9.1.3.

Comparison between ARGs present in assembled and raw data was performed analyzing paired unassembled and assembled metagenomes from 5 gut samples from different subjects (Subjects ID: 159005010, 159247771, 159369152, 763961826, and 246515023; Supplementary Table 3) and from five buccal mucosa samples from 5 different subjects (Subjects ID: 370425937, 764325968, 604812005, 246515023, and 809635352; Supplementary Table 3). ARGs in assembled data were detected with BLASTp as mentioned above. Detection of ARGs in raw data was performed with two different strategies: DeepARG (Arango-Argoty et al., 2018), a machine learning algorithm that detects ARGs and normalizes it by the number of 16S rRNA gene (90% identity, *e*-value ≤ 10^–10^), and comparing the reads with BLASTx against the antibiotic resistance databases CARD (Jia et al., 2017), ARG-ANNOT (Gupta et al., 2014), and RESFAMS (Gibson et al., 2015) (*e*-value ≤ 10^–5^, amino acid identity ≥ 90%, and bit-score ≥ 70) and normalized by the unassembled Mb.

### Statistical Analysis

One-way ANOVA was performed to compare the ARG abundance (ARG/Mb) in each body site between samples from women and men.

Comparison between ARGs hits/Mb was performed with Welch test and pairwise *t*-test in R (R Core Team, 2014). *p*-Value ≤ 0.05 was considered as significant in all the statistical test performed.

Principal coordinates analysis (PCoA) was performed calculating the distance matrix using the Euclidean distance and plotted with ggplot (Wickham, 2016). For the different sites of the body, it was studied the relative abundance of each ARG categorized by antibiotic class resistance.

## Results

### Comprehensive Metagenomic Characterization of the Human Resistome

The HMP (Huttenhower et al., 2012) aimed to characterize the diversity and metabolic potential of the microbiomes of healthy human subjects from different body sites. In this study, we analyzed the resistome (i.e., pool of ARGs) of these body parts, which examines a total of 771 HMP samples from the oral cavity, skin, nares, vagina, and gut (Supplementary Tables 1, 4). Detection of ARGs was performed using amino acid sequence similarity searching against the following reference ARG databases (refer to Methods for details).

From all the detected HMP proteins (9.17E + 07), a total of 28,714 ARG hits were found, which represents between 0.02 and 0.08% of the relative abundance of HMP proteins depending on the body site analyzed (Supplementary Table 4). Overall, nearly all analyzed samples (99%) from the different human body sites showed at least one ARG. The exceptions were some specific HMP samples from the nares (≈14% of nares samples), skin (4.25% of skin samples), and vagina (45% of vagina samples), in which no ARG was detected (Supplementary Table 4).

**FIGURE 1:**
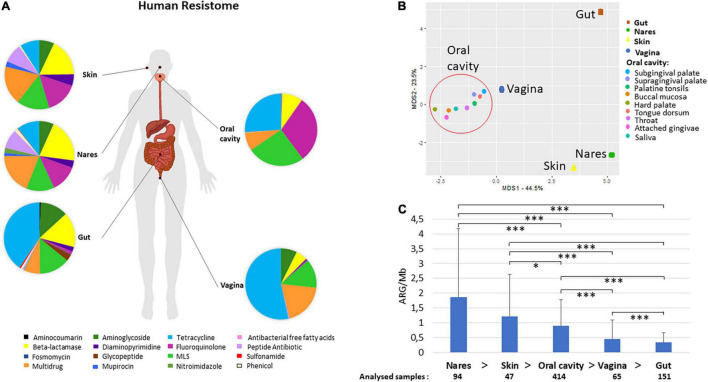
Human resistome. Human atlas of the ARGs grouped by drug class their confer resistance to present in different body parts. The body groups studied were the gut, the skin (retro-auricular crease), vagina (posterior-fornix, mid vagina, and vagina intraotus), the nares, and the oral cavity (hard palate, buccal mucosa, saliva, subgingival plaque, attached gingivae, tongue dorsum, throat, palatine tonsils, and supragingival plaque) **(A)**. PCoA of the different body sites distributed according to their relative abundance of AR to different drug classes **(B)**. The samples included in the group oral cavity (hard palate, buccal mucosa, saliva, subgingival plaque, attached gingivae, tongue dorsum, throat, palatine tonsils, and supragingival plaque –shaped as a circle) gathered together and separately from nares, skin, and gut samples. Abundance of ARGs calculated as ARGs hits per assembled Mb and number of samples included in each body group **(C)**. Welch test was performed to compare ARG abundances between different body sites. All paired samples showed statistically significant differences but the nares and the skin. *p*-values (P) considered as significant were indicated with an asterisk: *p ≤ 0.05, ***p ≤ 0.001.

On average, tetracycline resistance genes were the most abundant ARGs in the HMP dataset (Figure 1A), followed by MLS or fluoroquinolone resistance genes, the ranks of which were dependent on the analyzed body site. Tetracycline resistance genes were the most frequent ARGs in vagina (53.40%) and gut (40.52%), whereas their percentage decreased in oral cavity, skin, and nares (26.02, 9.03, and 10.45%), where the most dominant ARGs were fluoroquinolone (30%), multidrug (18.22%), and beta-lactamase resistance genes (≈20%), respectively (Figure 1A). In gut samples, the second-most abundant resistance genes were the ones conferring resistance against beta-lactams (as in skin), while in the vagina, the second-most abundant was multidrug resistance genes (19.37%). Aminocoumarin resistance genes were only found in the gut, whereas peptide ARGs (e.g., *ugd, yojI*, and *mprF*) were found in all body parts analyzed and they were more frequent in skin and nares (representing a sixfold increase compared with the relative abundance of this antibiotic class resistance gene in the rest of the body).

All samples from the oral cavity (hard palate, buccal mucosa, saliva, subgingival and supragingival plaque, attached/keratinized gingivae, tongue dorsum, throat, and palatine tonsils) showed a similar pattern of resistance to the different antibiotic classes with minor variations (Figure 1B and Supplementary Figure 1). Separated from the oral cavity, the skin and nares showed similar dominant ARGs grouped by drug classes [fluoroquinolone, multidrug, macrolide–lincosamide–streptogramin (MLS), and beta-lactamase resistance], although the ARG in nares displayed resistance to 14 different drug classes, while ARG present in skin displayed resistance to 10 different drug classes. The bacteria from the vagina had resistance against 8 antibiotic classes, being the lowest number of the 5 body parts compared in this study (the top three ARGs ranked were tetracycline, fluoroquinolone, and MLS resistance genes). Remarkably, nares and gut showed resistance to the highest number of antibiotic classes (14 out of the 16 different classes found in this study).

As shown in Figure 1C, the body part that had the highest abundance of ARGs per assembled Mb was the nares (1.86 ± 2.32 ARGs/Mb), followed by the skin (1.22 ± 1.42 ARGs/Mb) and oral cavity (0.90 ± 0.88 ARGs/Mb) (Figure 1C). It is worth noting that the gut (0.34 ± 0.33 ARGs/Mb) had the lowest amount of ARGs per Mb among all the analyzed body parts (Figure 1C). The Welch test that employed to compare the abundance of different body parts showed statistically significant differences (*p*-value ≤ 0.05) between almost all body parts but not between the skin and nares (Figure 1C). No significant differences were found between male and female subjects in any of the body sites analyzed (Supplementary Figure 2). According to recent estimates of the average genome size (AGS) of human microbes from different body parts of HMP datasets (Nayfach and Pollard, 2015), in general, the correlation of ARGs and the AGS indicated that the number of ARGs per bacterial genome ranged from 1.3 in stool (AGS = 3.9 Mb) to 3 in nares (AGS≈2.5 Mb).

### Characterization of Dominant Antibiotic Resistance Genes in the Human Body

In this section, beyond the above-described diversity and abundance of ARG classes in the HMP dataset, we sought to study in detail the pool of different types of ARGs and the identity of antibiotic-resistant microbes harboring these ARGs. Within each of the antibiotic classes, different types of ARGs are described in databases [2,404 in CARD (Jia et al., 2017), 2,038 in ARG_ANNOT (Gupta et al., 2014), and 3,169 in RESFAMS (Gibson et al., 2015)]. In addition, based on the antibiotic mechanism of action (Reygaert, 2018), five types of antibiotics are defined: antibiotics that (1) inhibit cell wall synthesis (e.g., beta-lactams), (2) depolarize the cell membrane (e.g., lipopeptides), (3) target nucleic acid synthesis (e.g., quinolones), (4) inhibit metabolic pathways (e.g., sulfonamides), and (5) affect protein synthesis (e.g., MLS antibiotics or tetracyclines) (Reygaert, 2018). Furthermore, ARGs could provide protection against one specific antibiotic or different types of antibiotics.

In the HMP datasets, after comparison with all three of the above ARG databases, a total of 235 different type of ARGs were found in all the analyzed samples (Supplementary Tables 5, 6). The gut samples had 155 different ARGs, the highest number and diversity among the analyzed body sites, whereas the lowest ARG diversity was found in the vagina (37) (Supplementary Table 6). The most abundant type of ARG in the oral cavity was *patB*, which provides resistance to fluoroquinolones *via* antibiotic efflux (ARO: 3000025). The *mprF* gene was the predominant ARG in the nares and skin, whereas *tetQ* was the most common ARG in the gut, and in the vagina, the most frequent gene was *tetM* (Supplementary Table 6). ARGs that are considered as high risk for clinic (Rank I) due to their wide host range mobility and niche adaptation (Zhang et al., 2021) were found less abundant in the analyzed datasets, for instance, *dfrA*14 and *dfrA*17 in gut or *blaZ* and *tetM* in skin, nares, and oral cavity.

**FIGURE 2:**
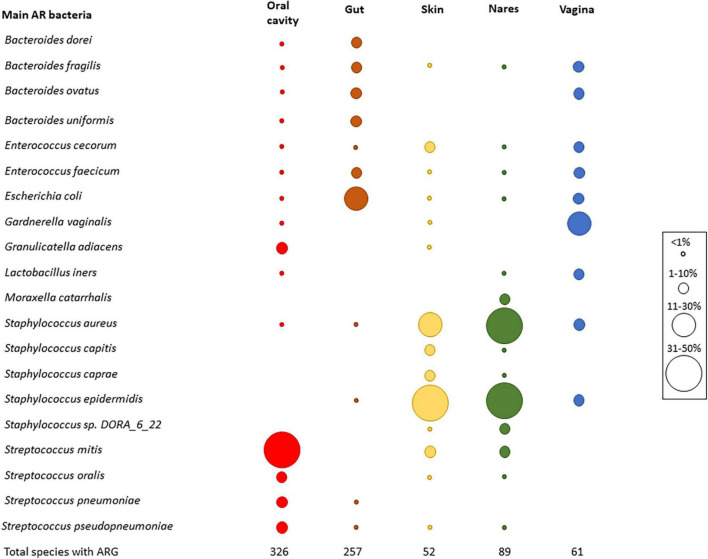
Main AR bacteria in HMP dataset. Relative abundance of the most abundant resistant bacteria. Top five bacteria were chosen in each body part, and then, the graph was completed with the relative frequency of all the chosen bacteria in all body parts. Circle sizes were different to determine the relative abundance of each species, and colors were used to differentiate the body parts (red—oral cavity, brown—gut, skin—yellow, green—nares, blue—vagina). At the bottom of the graph, the number of different species that carried ARGs is shown.

Regarding the identification of the most common antibiotic resistant (AR) bacteria in HMP datasets (Figure 2) based on the best-hit score, as expected, the results differed among body parts. The oral cavity had 326 different species harboring ARGs, followed by the gut (257 different species). The skin showed the lowest number of different species with ARGs (a total of 52) (Figure 2). *Streptococcus mitis* was the most abundant AR bacterium in the oral cavity. In the gut, the most abundant AR bacterium was *Escherichia coli*, while in nares and skin, *Staphylococcus* was the predominant AR bacterium (*Staphylococcus aureus* in nares and *Staphylococcus epidermis* in skin). Finally, *Gardnerella vaginalis* was the most abundant resistant species in the vagina. *S. aureus*, *E. coli*, and *Bacteroides fragilis* were the most abundant AR bacteria found in all body sites (Figure 2).

**FIGURE 3:**
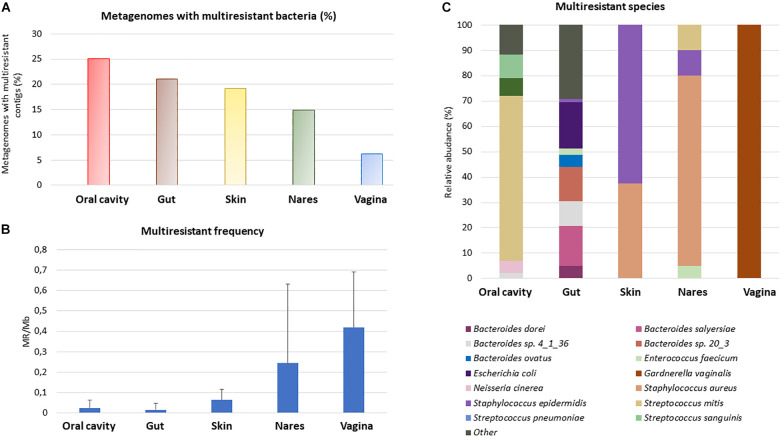
Multiresistance in the human body. Those assembled genome fragments (i.e., contigs) that had more than one ARG conferring resistance to at least 2 different antibiotic families were considered as multiresistant. Percentage of metagenomes with multiresistant contigs compared with all the metagenomes studied from the same HMP sample **(A)**. Study of the multiresistant contigs frequency in metagenomes with at least one multiresistant contig **(B)**, to compare the different samples, the number of multiresistant contigs was divided by the assembled Mb. Standard deviation is shown in the graph. Relative abundance of the most abundant MR **(C)**. Only MR whose relative abundance was, at least in one body site, equal or greater than 5% was represented.

The increasing multiantibiotic-resistant bacteria (MRBs) are a major threat to human health. We next attempted to identify genome fragments (i.e., contigs) having two or more ARGs which provide insights into multi- antibiotic resistance (MR) bacteria in HMP datasets. For this purpose, we applied the criteria for the detection of more than one ARG in the same assembled genome fragment (which could also be a plasmid or a plasmid fragment), which confers resistance to at least two different antibiotic classes in each of the analyzed HMP samples. The percentage of metagenomic samples from the HMP with the presence of MR was between ≈25% (oral cavity) and 6% (vagina). A total of 21% of the analyzed gut samples had > 1 contig conferring MR potential, whereas in the skin, the percentage was 19%, and in nares, 15% of the samples showed MR (Figure 3A). The MR frequency changed depending on the studied group. Vagina samples showed the highest MR-related contig frequency, with a large difference among vaginal samples (0.42 ± 0.27 MRB/assembled Mb). The skin, oral cavity, and gut had the lowest frequency of MR (Figure 3B).

The most predominant resistant bacterial species (Figure 2) were found to be also the most abundant MR species in each body site (Figure 3C). The MR profile was different depending on the sampling site. In the vagina, there was only one MR species whereas in skin, there were only 2 main species carrying more than one ARG, and the gut had 23 MR species, with the highest number of different MR found in all body sites. None of the MR species were found in all the body parts. In fact, 6 species (*Bacteroides* sp. *4_1_36, Bacteroides fragilis, Enterococcus faecium, Staphylococcus aureus, Staphylococcus epidermidis*, and *Streptococcus mitis*) out of the 33 MRBs found were in two or three different parts of the body (Supplementary Figure 3).

When all of the above ARGs detected in healthy humans were clustered (≥90% amino acid identity) to study a highly conserved core of shared ARGs, it was observed that there were 3 common ARGs in the 5 body parts analyzed. One, MFS-type efflux protein (*msrD*), was related to resistance to macrolides. The other 2 genes were related to ribosomal resistance against tetracycline (*tetO* and *tetQ*) associated with conjugative plasmids or transposons. In feces, *tetO* was found not only in bacteria belonging to the phylum Firmicutes (*Clostridiales bacterium VE202-13;* Ga0104838_1543581) but also in bacteria of the phylum Actinobacteria (*Trueperella pyogenes MS249;* Ga0111491_10662371).

### Detection of Human Microbiome Project Antibiotic Resistance Genes in Pristine Environments

Resistomes and ARGs dispersion from hot spots such as wastewater plants or hospitals to downstream aquatic environments have been extensively studied and characterized (Rowe et al., 2017; Ju et al., 2018; Khan et al., 2019). Although the presence of ARGs in environments with low or scarce human intervention has been explored (Van Goethem et al., 2018; Naidoo et al., 2020), to the best of our knowledge, it has never been explored whether common ARGs from HMP datasets are present in autochthonous bacteria from different pristine environments.

**FIGURE 4:**
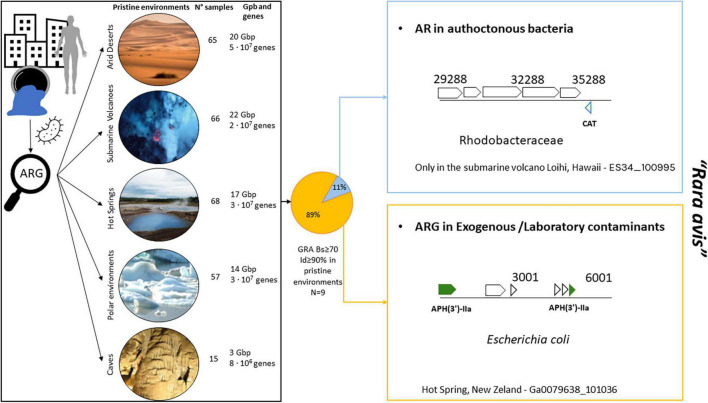
Detection of ARGs from HMP dataset in pristine environments [arid deserts (*n* = 65), submarine volcanoes (*n* = 66), hot springs (*n* = 68), polar environment (*n* = 57), and caves (*n* = 15)]. Only 9 ARGs were found in pristine environments according to our criteria (refer to Methods and Results). The only case of ARG found in an autochthonous bacterium in pristine environments was that of a chloramphenicol acetyltransferase (CAT) gene belonging to *Salmonella* sp. (100% identity with Ga0111015_155701; a nares sample) present in a marine bacterium found in Loihi (a submarine volcano) from the family *Rhodobacteraceae*. The presence of CAT from *Enterobacteriaceae* in *Rhodobacter* has been previously described in the coastal water of Jiaozhou Bay (Dang et al., 2008). Chloramphenicol-resistant bacteria often harbor plasmids carrying the CAT gene (Shaw et al., 1979) that could have been transferred to *Rhodobacter*. Desert photo taken from Boris Ulzibat (PEXELS). Submarine volcano photograph courtesy of NOAA/NSF/WHOI page (https://oceanexplorer.noaa.gov/facts/volcanoes.html).

To determine the presence of ARGs from the HMP in pristine environments (Figure 4; polar, desert, cave, hot spring, and submarine volcano environments; Supplementary Table 2) with no *a priori* anthropogenic influence, proteins from 271 different pristine environments (i.e., metagenomic datasets) were screened to search for ARGs detected in the analyzed HMP samples. Only those proteins with identity ≥ 90%, with bit-score ≥ 70 and belonging to genomic scaffolds with at least 4 proteins were considered for further analysis. It is important to remark that if an ARG from the HMP dataset is detected in a genome fragment from a pristine environment, two different hypotheses could be considered: this detected ARG in pristine environments was (1) an allochthon HMP gene dispersed from anthropic areas that was acquired by autochthonous bacteria inhabiting the pristine environment, or (2) this ARG in pristine environments is actually the result of contamination during sample manipulation, collection, or post-processing (e.g., DNA contaminant fragments in reagents from kits, DNA sequencing, and other metagenomics-related experiments) and thus is not truly present in these pristine environments.

In the 271 analyzed samples from pristine environments (a total of 77 Gb of sequencing information and 2.1E + 08 analyzed proteins), we detected a total of 9 ARGs from HMP dataset. Only one of those ARGs were found in a genome fragment of a putative autochthonous bacterium from the family *Rhodobacteraceae* recovered in a submarine volcano (Figure 4 and Supplementary Table 7; a chloramphenicol acetyltransferase gene 100% amino acid identical with the HMP gene dataset). The rest and great majority of detected ARGs in pristine environments were simply exogenous contaminant present in these metagenomes from manipulation or laboratory reagents. For instance, it is obvious that *Escherichia coli* should not be detected in hot springs. However, we indeed found ARGs in *E. coli* genome fragments in the corresponding hot spring metagenomes (Figure 4, bottom right panel).

## Discussion

The human resistome has received increased attention in the recent years due to its impact in our society. Usually, resistome studies focus their attention on one body site, usually studying the gut (Hu et al., 2013; Palleja et al., 2018) or, more recently, the skin (Li et al., 2021). To our knowledge, only one study has compared resistome traits from the gut with different parts of the oral cavity, examined *via* different protocols (Carr et al., 2020). In our study, the advantage of using only HMP samples that were subjected to standardized procedures was the elimination of biases and variability introduced by contrasting procedures from different surveys (Huttenhower et al., 2012). Here, in our study, we found that the most abundant ARGs in the HMP resistome provided resistance against fluoroquinolone, MLS, and tetracycline resistance genes, followed by multidrug resistance genes and beta-lactams. Members of these antibiotic classes were among the most commonly prescribed oral antibiotics in 2010 (Hicks et al., 2013), right before the samples were obtained, which shows a plausible relation between the consumed antibiotics and the detected resistance in American subjects, even though we cannot rule out the influence of antibiotics consumed through the food (Lee, 2003; Salyers et al., 2004). A human gut study from Chinese, Spanish, and Danish subjects showed that more than 75% of the ARGs were tetracycline resistance genes, MLS resistance genes, and beta-lactams (Hu et al., 2013). This was consistent with our data since these three antibiotic classes accounted for 61% of the relative abundance found in our study with HMP samples (Van Boeckel et al., 2014). The characterization of resistomes from metagenomic data can also be performed from unassembled data (Arango-Argoty et al., 2018; Maestre-Carballa et al., 2019). Here, the analysis from unassembled data (Supplementary Figure 4 and Supplementary Table 3) showed that major ARGs grouped by drug classes relative abundance were similar to the one obtained with assembled data shown in Figure 1. Even though, we cannot rule out that the normalized abundances (no. of ARG per Mb) could be biased by the metagenomic assembly step or by the very short lengths of Illumina DNA reads and the predicted amino acid sequences obtained from the HMP datasets (Supplementary Figure 5).

The different physiological conditions bacteria–host interactions, AGS (Nayfach and Pollard, 2015), and especially, diversity of bacterial species (Forsberg et al., 2014) present in each body part could be the important factors contributing to the differences in ARG abundance, which were statistically significant for different paired body parts analyzed, except in the skin–nares pair (Figure 1C). In addition, the well-known interindividual variability in the human microbiome was also observed here for ARG abundance. The highest ARG abundance was found in the nares, a body entrance for microorganisms carried by air, which could include pathogenic bacteria such as *Legionella* or *Mycobacterium* species. Airborne bacteria could also carry ARGs (Li et al., 2018); therefore, ARGs could first arrive at the nares. It has been calculated that we inhale 7 m^3^ of air and 10^4^–10^6^ bacterial cells per cubic meter of air per day (Kumpitsch et al., 2019) albeit the quantity varies depending on different factors, such as geography, weather, micro-niches, and air pollution (Li et al., 2018; Kumpitsch et al., 2019; Zhang et al., 2019). In addition, seasonal variation in bacterial species in the nares environment has been observed (Camarinha-Silva et al., 2012). However, considering that bacteria present on the nares surface, in contrast to gut or oral bacteria, are not typically in “direct contact” with antibiotics, it is certainly surprising that the nares microbiome maintains the highest rate of ARG abundance, and more intriguing are the mechanisms used to acquire and fix these ARGs.

As shown in this study, the number of ARGs per assembled Mb in the gut was lower than that in the other body parts, but the ARG richness was greater. This observation is consistent with the results of Carr et al. (2020), who compared oral and fecal samples. Even though the abundance was measured with other parameters [reads per kilobase of read per million (RPKM) and coverage greater than 90%], the ARG abundance in stool was smaller than that in oral samples from China, the United States, and Fiji but not western Europe (Carr et al., 2020). Carr et al. (2020) hypothesized that different niches in the oral cavity, such as the dorsum of the tongue, could aid the deposition of debris and microbes or even the formation of biofilms, which are the structures that favor HGT between different species (Giaouris et al., 2015). Given the high bacterial richness estimated in stool, compared with other body parts (Huse et al., 2012), the low ARG abundance per assembled Mb in gut could be explained because the AR bacteria found in this study, such as *Escherichia coli* (Figure 2), represent only a 1% of the intestinal microbiota (Delmas et al., 2015). Other resistant species such as *Bacterioides uniformis* and *B*. *fragilis* were also found to have low abundance in human stool (0.1% of 16S rRNA amplified genes) (Hong et al., 2008).

Regarding the bacterial species with antibiotic resistance, consistent with the other studies, we found that commensals such as *Staphylococcus aureus* and *Staphylococcus epidermidis* in skin were the top 10 AR bacteria (Li et al., 2021). Further, some of them, such as *S. aureus*, were multidrug-resistant bacteria, with a total of 3 ARGs in skin (*blaZ, aadD*, *ermC*) conferring resistance to 3 different antibiotic classes (beta-lactam, aminoglycoside, and MLS; JGI scaffold ID Ga0105645_100266). Additionally, as expected, methicillin-resistant *S. aureus* (MRSA), which is listed among the CDC’s Antibiotic Resistance Threats in the United States (Centers for Disease Control and Prevention, 2019), was present naturally in nares from different subjects. Another species found in oral HMP samples listed in the AR Threats report was *Streptococcus pneumoniae*.

Many of the most abundant ARGs found in this study, such as efflux pumps (e.g., *parB* or *msrD*), are usually considered as not sensu stricto ARG. Indeed, in many cases, these genes do not represent a major concern in medicine, but they are only used as the indicator of resistant bacterial species, such as *mprF* (Martínez et al., 2014). To a lesser extent, we also found ARGs that are considered as high risk in medicine (Rank I) due to their wide host range mobility and niche adaptation (e.g., *blaZ*, *tetM, dfrA*14, or *dfrA*17) (Zhang et al., 2021).

Even though the presence of ARGs has been detected in pristine environments and therefore they could be considered as the potential ARG sources (Van Goethem et al., 2018; Naidoo et al., 2020), we consider that our results provide hope, since the dispersion of ARGs detected in the HMP dataset to pristine environments is extremely infrequent and anecdotical, with only one ARG in an autochthonous bacterium among dozens of millions of analyzed genes. Furthermore, even using more relaxed thresholds, it can be considered as a *rara avis* event. As shown in Figure 4, nearly all detected ARGs from pristine environments actually belonged to laboratory contaminants or exogenous bacteria that were not obviously found in these habitats (e.g., *E. coli* in hot springs), since it is extremely unlikely that those species colonize pristine environments, whose ecology differs greatly from the human tissues. Sometimes, a general metagenomic analysis could mislead the interpretation of the data if sequencing and genomic assembled data are not carefully inspected. Our study exemplifies very well this bias since an initial ARG search detection indeed discovered a certain number of ARGs, but later on, it was demonstrated that they were clearly not naturally present in these pristine environments.

Finally, it is important to discuss the potential caveats and biases of our study. Here, we have used sequence similarity-based searches with strict conservative thresholds for detecting ARGs in metagenomics datasets to avoid false positives. Only hits with amino acid identity ≥ 90% and bit-score ≥ 70 against ARGs deposited in curated reference antibiotic resistance databases were considered. This methodology has been widely used in the previous publications (Van Goethem et al., 2018; Chng et al., 2020; Lira et al., 2020). Obviously, unknown ARGs yet to be discovered or not present in our reference ARG databases cannot be detected using our methodology. Likewise, probably, our approach has ruled out some actual ARGs present in samples that display a score similarity below our thresholds (i.e., false negatives). However, it is worth noting, as highlighted in the previous studies using our methodologies, that more rigorous thresholds are clearly preferred. It is very interesting to read the discussion on how to use less strict thresholds when detecting ARGs in viruses can profoundly mislead data interpretation (Enault et al., 2017). This is even more important when analyzing datasets from pristine environments since a conservative approach is preferable over using riskier thresholds. Even though we have used strict thresholds to detect only *bona fide* ARGs, it may be noted that some genes could “escape” from this filter. For instance, some housekeeping genes (constitutive genes required for basic cellular functions) only require one or few mutations to conferring antibiotic resistance [e.g., *rplS* (Torii et al., 2003), *parY* (Schmutz et al., 2003), and *gyrA* (Avalos et al., 2015)]. For instance, the mutant version of the housekeeping gene *gyrA* found in common antibiotic resistance databases used in this study typically displays mutations in a very short motif called “QRDR” that is responsible for quinolone resistance (Avalos et al., 2015; Jia et al., 2017). However, in our search in HMP datasets, despite having high similarity and above our thresholds, the great majority of detected *gyrA* proteins in HMP did not have this motif (Supplementary Figure 6) and therefore was totally unclear whether they confer antibiotic resistance. Similar cases were found for other housekeeping genes, even when they displayed high sequence similarity. Thus, to avoid including false positives that would overestimate ARG abundance, housekeeping genes with mutation conferring resistance (included in the ARG databases used) were ruled out from our analysis.

Overall, our study provides a comprehensive analysis of the human microbiome resistomes from different body sites studied by the HMP consortium, which provides valuable biological insights that can serve as the baseline for further studies and be thus integrated into AMR surveillance protocols to determine the fate of the diversity and abundance of ARGs in the long term. Our data also show that the level and impact of ARGs spreading and selection pressure to fix these alleles in non-anthropogenic areas is negligible. However, it is in our hands, as a society, to control these selection pressures and, if possible, reverse and ameliorate the impact of ARGs in nature.

## Data Availability Statement

The original contributions presented in the study are included in the article/Supplementary Material, further inquiries can be directed to the corresponding author/s.

## Ethics Statement

This study does not require ethical approval because it uses information freely available in the public domain.

## Author Contributions

MM-G conceived the research. LM-C gathered the data. MM-G and LM-C analyzed, interpreted the data, and wrote the manuscript. VN-L and MM-G revised the manuscript and provided funds. All authors have approved the submitted version.

## Conflict of Interest

The authors declare that the research was conducted in the absence of any commercial or financial relationships that could be construed as a potential conflict of interest.

## Publisher’s Note

All claims expressed in this article are solely those of the authors and do not necessarily represent those of their affiliated organizations, or those of the publisher, the editors and the reviewers. Any product that may be evaluated in this article, or claim that may be made by its manufacturer, is not guaranteed or endorsed by the publisher.

## Footnotes

1
https://www.hmpdacc.org/HMASM/
