# Supplementary material for: A Resistome Roadmap: From the Human Body to Pristine Environments
Source: Front Microbiol. 2022 May 11;13:858831. doi: 10.3389/fmicb.2022.858831 (PMC9134733; doi:10.3389/fmicb.2022.858831)
Supplement: Supplementary file 1 [file Data_Sheet_1.docx]

**Supplementary Information**

**This supplementary information contains:**

**-6 Supplementary Figures**

**-7 Supplementary Tables**

**-Reference section**


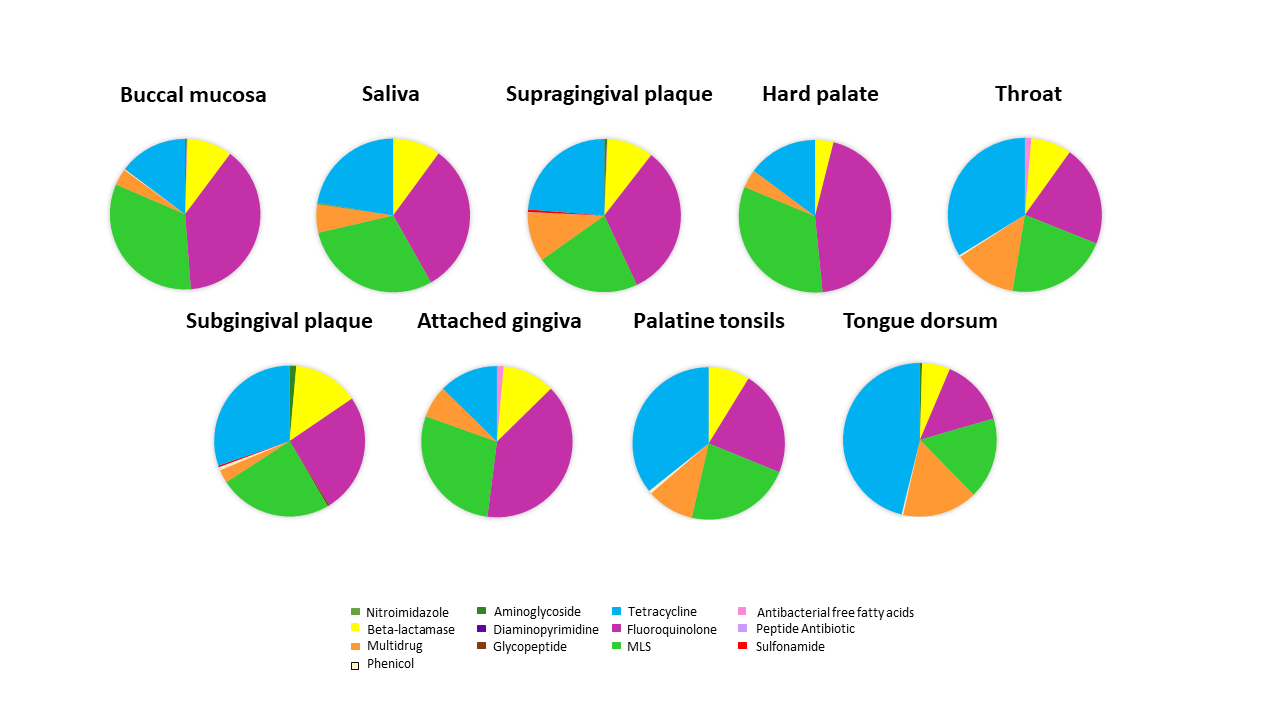


**Supplementary Figure 1. Antibiotic resistant profile: oral cavity subsamples.** Antibiotic resistance profile of the subsamples used to study the oral cavity. Similar relative abundance of antibiotic resistance distribution was found.


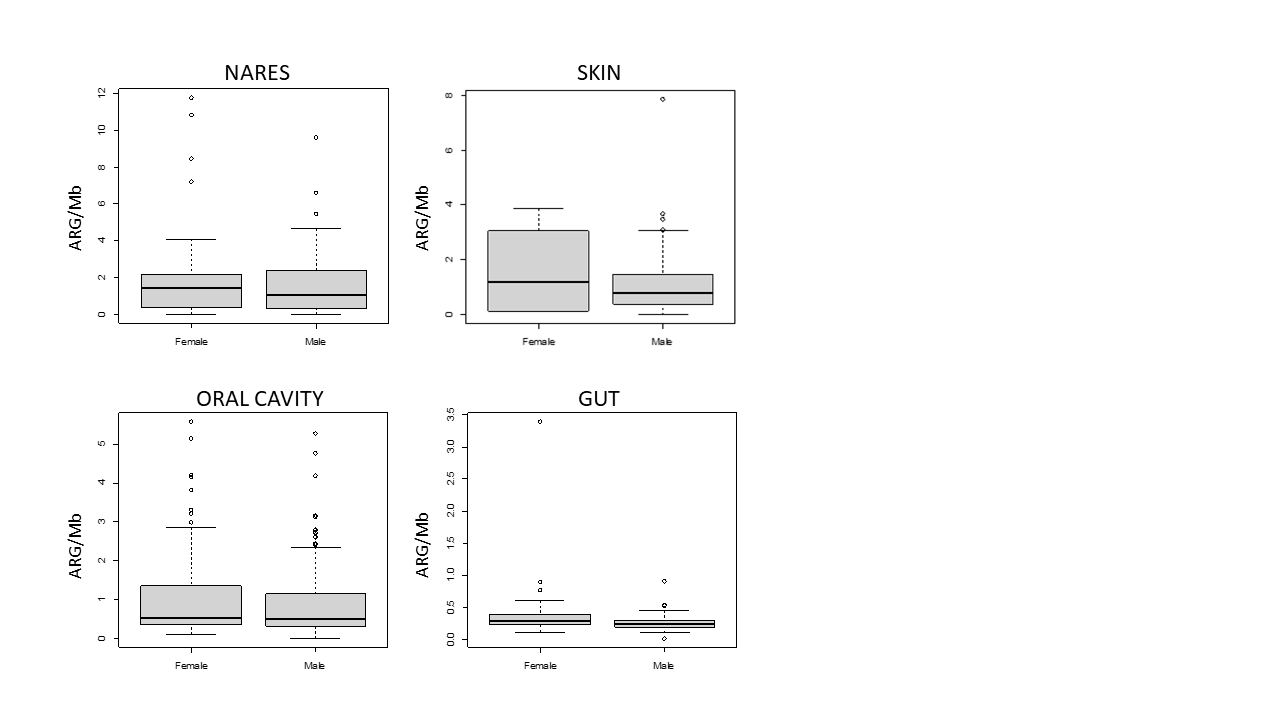


**Supplementary Figure 2.** **Antibiotic resistant abundance: sex comparison.** Even though not statistically significant differences were found in the ARG abundance (measured as ARG/Mb; one-way ANOVA statistical test) between the female and male subjects, woman had in all body sites analysed more ARGs abundance than man.


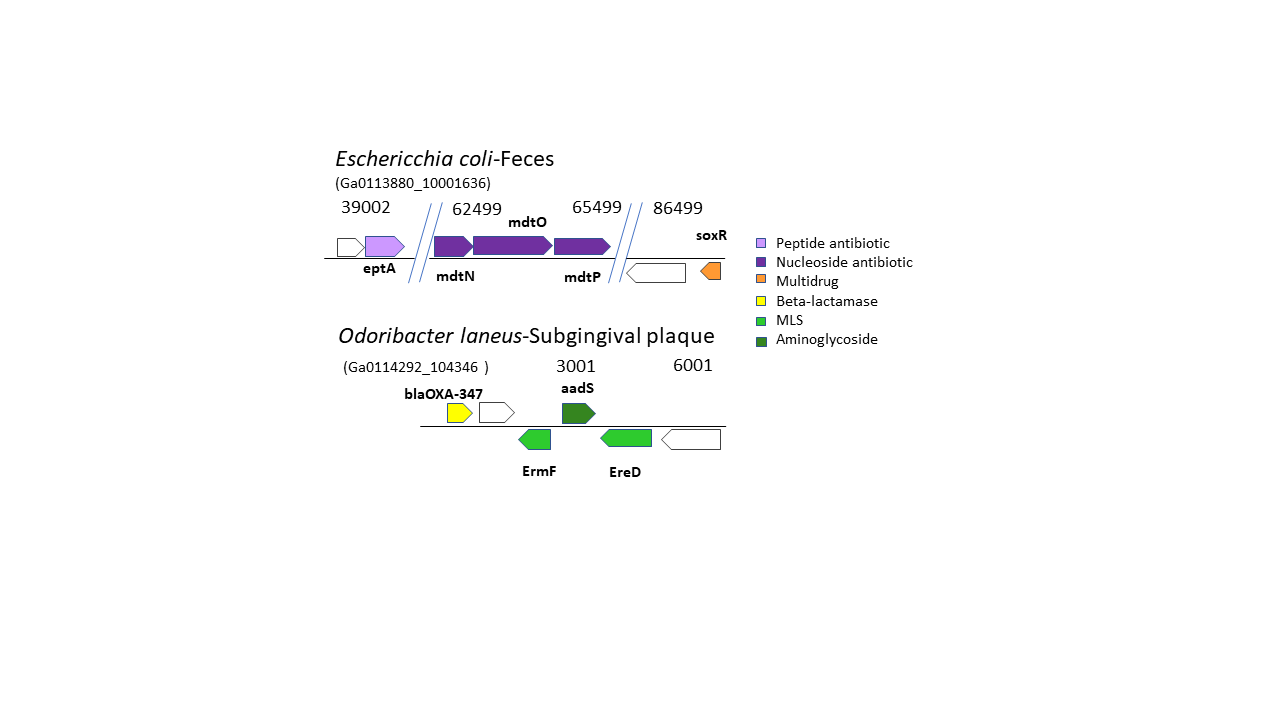


**Supplementary Figure 3. Example of two multirresistant bacteria found in the human body.** Two multiantibiotic-resistant contigs found in the human body. They presented 5 and 4 different ARGs each, that are coloured according to the antibiotic to whom they confer resistance to. Taxonomic classification and gene annotation was obtained from the JGI-IMG/ER (Chen et al., 2021).


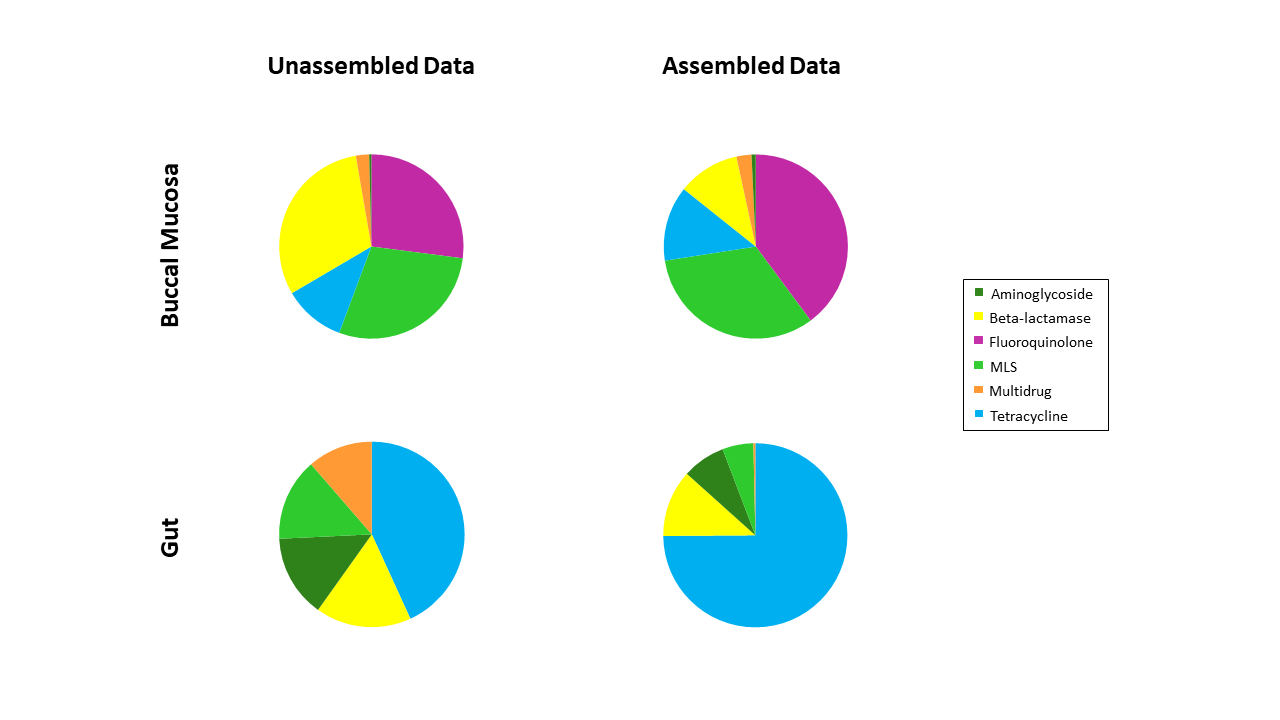


**Supplementary Figure 4. Relative abundance of ARGs grouped by antibiotic class they confer resistance to in assembled and unassembled data.** Comparison of the more abundant ARGs classes in unassembled and assembled paired data for 5 buccal mucosa samples (upper panel) and 5 gut samples (bottom panel). Similar ARGs classes relative abundances were found between assembled and unassembled data for each body parts studied.


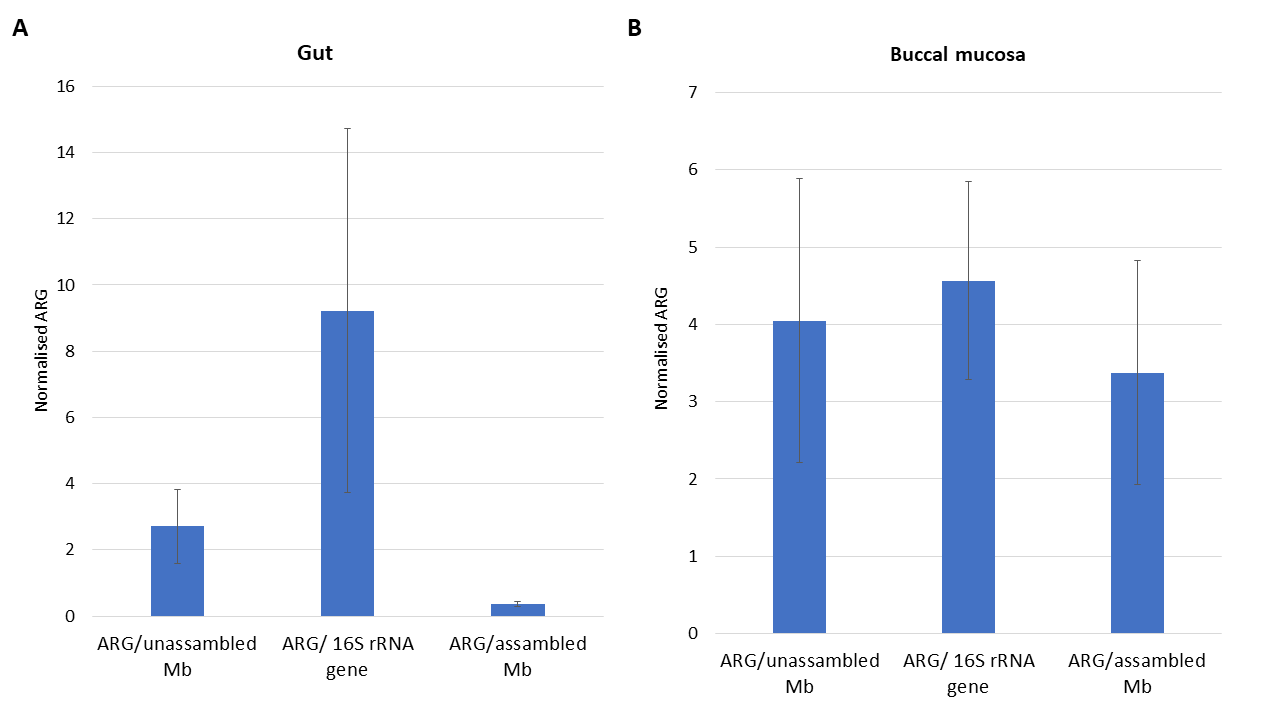


**Supplementary Figure 5. Abundance of ARGs in two body sites.** Study of the ARG abundance in raw and assembled data for 5 gut samples (A) and 5 buccal mucosa samples (B). Three different methods were employed to study the ARG abundance. ARGs in raw data were analyzed using BLASTx against the antibiotic resistance databases CARD (Jia et al., 2017), ARG-ANNOT (Gupta et al., 2014) and RESFAMS (Gibson et al., 2015) (e-value ≤ 10^-5^, amino acid identity ≥ 90% and bit-score ≥ 70) and normalised by the unassembled Mb or with DeepARG (Arango-Argoty et al., 2018), which normalized the data by 16S rRNA gene (Identity 90%). Assembled data was analysed against the same databases (and parameters) mentioned above but using BLASTp.


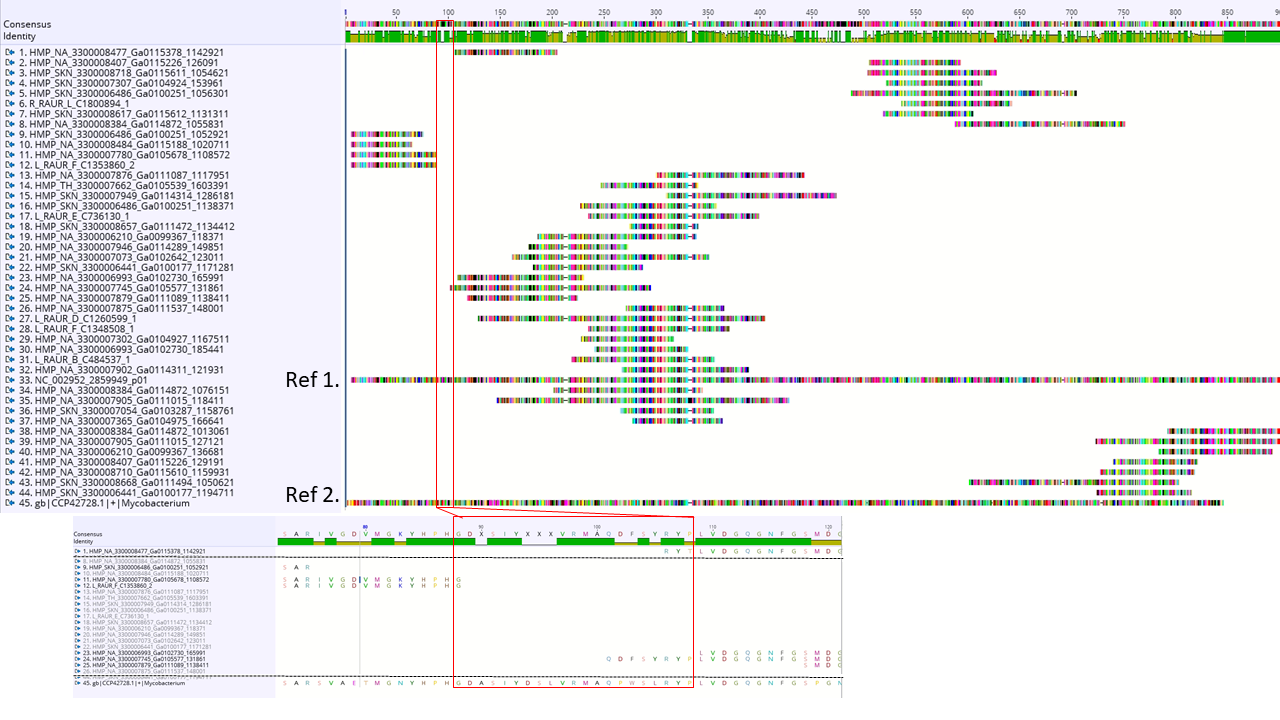


**Supplementary Figure 6. GyrA protein alignment.** All the proteins from the HMP dataset classified as the housekeeping gene gyrA were extracted and aligned in order to find the mutations that confer them resistance against quinolones (Avalos et al., 2015; Jia et al., 2017). For that gyrA proteins were compared with two gyrA fluoroquinolone resistant genes from *Staphylococcus* (NC_002952_2859949_p01; Ref 1.) (Gibson et al., 2015) and Mycobacterium tuberculosis ([>gb|CCP42728.1|+|Mycobacterium tuberculosis gyrA conferring resistance to fluoroquinolones [Mycobacterium tuberculosis H37Rv]](http://www.ncbi.nlm.nih.gov/protein/CCP42728.1)) (Jia et al., 2017). Red lines are used to highlight the quinolone resistance determining region (QRDR) where certain mutations confer fluoroquinolone resistance. A great majority of the gyrA proteins did not align with QRDR region. The top panel has been cropped to observe the detail of the alignment (bottom panel).

**Supplementary Table 1.** **Human Microbiome Project samples.** To characterize the Human Microbiome Project (HMP) resistome, 771 body metagenomes were analysed. Shotgun-metagenome data was obtained either from JGI/IMG-ER (IMG Genome ID) (Markowitz et al., 2012) or HMP web page (SRS Code) (Huttenhower et al., 2012).

| **Genome Name / Sample Name** | **IMG Genome ID/SRS Code** | **Obtained from** |
| --- | --- | --- |
| Human anterior nares microbial communities from NIH, USA - visit 2, subject 763759525 | 3300006202 | JGI/IMG-ER |
| Human anterior nares microbial communities from NIH, USA - visit 1, subject 765034022 | 3300006205 | JGI/IMG-ER |
| Human anterior nares microbial communities from NIH, USA - visit 2, subject 158883629 | 3300006215 | JGI/IMG-ER |
| Human anterior nares microbial communities from NIH, USA - visit 1, subject 763901136 | 3300006491 | JGI/IMG-ER |
| Human anterior nares microbial communities from NIH, USA - visit 2 of subject 158256496 | 3300006676 | JGI/IMG-ER |
| Human anterior nares microbial communities from NIH, USA - visit 2, subject 158944319 | 3300006964 | JGI/IMG-ER |
| Human anterior nares microbial communities from NIH, USA - visit 1, subject 763577454 | 3300006992 | JGI/IMG-ER |
| Human anterior nares microbial communities from NIH, USA - visit 2, subject 764062976 | 3300007009 | JGI/IMG-ER |
| Human anterior nares microbial communities from NIH, USA - visit 1, subject 764649650 | 3300007012 | JGI/IMG-ER |
| Human anterior nares microbial communities from NIH, USA - visit 1, subject 765013792 | 3300007073 | JGI/IMG-ER |
| Human anterior nares microbial communities from NIH, USA - visit 2, subject 158337416 reassembly | 3300007251 | JGI/IMG-ER |
| Human anterior nares microbial communities from NIH, USA - visit 1, subject 765337473 reassembly | 3300007260 | JGI/IMG-ER |
| Human anterior nares microbial communities from NIH, USA - visit 1, subject 158984779 reassembly | 3300007302 | JGI/IMG-ER |
| Human anterior nares microbial communities from NIH, USA - visit 1, subject 765640925 reassembly | 3300007365 | JGI/IMG-ER |
| Human anterior nares microbial communities from NIH, USA - visit 2, subject 764042746 reassembly | 3300007737 | JGI/IMG-ER |
| Human anterior nares microbial communities from NIH, USA - visit 1, subject 159915365 reassembly | 3300007837 | JGI/IMG-ER |
| Human anterior nares microbial communities from NIH, USA - visit 1, subject 160704339 reassembly | 3300007855 | JGI/IMG-ER |
| Human anterior nares microbial communities from NIH, USA - visit 1, subject 158458797 reassembly | 3300007865 | JGI/IMG-ER |
| Human anterior nares microbial communities from NIH, USA - visit 2, subject 764143897 reassembly | 3300007872 | JGI/IMG-ER |
| Human anterior nares microbial communities from NIH, USA - visit 1, subject 160643649 reassembly | 3300007876 | JGI/IMG-ER |
| Human anterior nares microbial communities from NIH, USA - visit 2, subject 159713063 reassembly | 3300007878 | JGI/IMG-ER |
| Human anterior nares microbial communities from NIH, USA - visit 1, subject 158742018 reassembly | 3300007890 | JGI/IMG-ER |
| Human anterior nares microbial communities from NIH, USA - visit 1, subject 160582958 reassembly | 3300007899 | JGI/IMG-ER |
| Human anterior nares microbial communities from NIH, USA - visit 1, subject 159753524 replicate 1 reassembly | 3300007902 | JGI/IMG-ER |
| Human anterior nares microbial communities from NIH, USA - visit 1, subject 508703490 reassembly | 3300007914 | JGI/IMG-ER |
| Human anterior nares microbial communities from NIH, USA - visit 1, subject 706846339 reassembly | 3300007920 | JGI/IMG-ER |
| Human anterior nares microbial communities from NIH, USA - visit 1, subject 737052003 reassembly | 3300007923 | JGI/IMG-ER |
| Human anterior nares microbial communities from NIH, USA - visit 1, subject 765620695 reassembly | 3300007934 | JGI/IMG-ER |
| Human anterior nares microbial communities from NIH, USA - visit 1, subject 764588959 reassembly | 3300007944 | JGI/IMG-ER |
| Human anterior nares microbial communities from NIH, USA - visit 1, subject 338793263 reassembly | 3300007946 | JGI/IMG-ER |
| Human anterior nares microbial communities from NIH, USA - visit 1, subject 158337416 reassembly | 3300008291 | JGI/IMG-ER |
| Human anterior nares microbial communities from NIH, USA - visit 2, subject 159247771 reassembly | 3300008329 | JGI/IMG-ER |
| Human anterior nares microbial communities from NIH, USA - visit 1, subject 159753524 reassembly | 3300008332 | JGI/IMG-ER |
| Human anterior nares microbial communities from NIH, USA - visit 2, subject 763840445 reassembly | 3300008381 | JGI/IMG-ER |
| Human anterior nares microbial communities from NIH, USA - visit 1, subject 160319967 reassembly | 3300008434 | JGI/IMG-ER |
| Human anterior nares microbial communities from NIH, USA - visit 1, subject 550534656 reassembly | 3300008710 | JGI/IMG-ER |
| Human anterior nares microbial communities from NIH, USA - visit 1, subject 160178356 | 3300006210 | JGI/IMG-ER |
| Human anterior nares microbial communities from NIH, USA - visit 1, subject 158479027 | 3300006422 | JGI/IMG-ER |
| Human anterior nares microbial communities from NIH, USA - visit 1, subject 763435843 | 3300006429 | JGI/IMG-ER |
| Human anterior nares microbial communities from NIH, USA - visit 2 of subject 763961826 replicate 2 | 3300006438 | JGI/IMG-ER |
| Human anterior nares microbial communities from NIH, USA - visit 2 of subject 764325968 | 3300006445 | JGI/IMG-ER |
| Human anterior nares microbial communities from NIH, USA - visit 2, subject 765560005 | 3300006490 | JGI/IMG-ER |
| Human anterior nares microbial communities from NIH, USA - visit 1, subject 764487809 | 3300006501 | JGI/IMG-ER |
| Human anterior nares microbial communities from NIH, USA - visit 1, subject 160218816 | 3300006724 | JGI/IMG-ER |
| Human anterior nares microbial communities from NIH, USA - visit 2, subject 763982056 | 3300006741 | JGI/IMG-ER |
| Human anterior nares microbial communities from NIH, USA - visit 2, subject 158499257 | 3300006976 | JGI/IMG-ER |
| Human anterior nares microbial communities from NIH, USA - visit 1, subject 764224817 | 3300006993 | JGI/IMG-ER |
| Human anterior nares microbial communities from NIH, USA - visit 1, subject 160421117 | 3300006994 | JGI/IMG-ER |
| Human anterior nares microbial communities from NIH, USA - visit 1, subject 159672603 | 3300007011 | JGI/IMG-ER |
| Human anterior nares microbial communities from NIH, USA - visit 1, subject 765701615 | 3300007014 | JGI/IMG-ER |
| Human anterior nares microbial communities from NIH, USA - visit 2, subject 158924089 | 3300007021 | JGI/IMG-ER |
| Human anterior nares microbial communities from NIH, USA - visit 1, subject 159814214 reassembly | 3300007246 | JGI/IMG-ER |
| Human anterior nares microbial communities from NIH, USA - visit 2, subject 764083206 replicate 1 reassembly | 3300007247 | JGI/IMG-ER |
| Human anterior nares microbial communities from NIH, USA - visit 1, subject 763860675 reassembly | 3300007359 | JGI/IMG-ER |
| Human anterior nares microbial communities from NIH, USA - visit 1, subject 160765029 reassembly | 3300007528 | JGI/IMG-ER |
| Human anterior nares microbial communities from NIH, USA - visit 1, subject 764083206 replicate 1 reassembly | 3300007739 | JGI/IMG-ER |
| Human anterior nares microbial communities from NIH, USA - visit 1, subject 159490532 reassembly | 3300007745 | JGI/IMG-ER |
| Human anterior nares microbial communities from NIH, USA - visit 1, subject 861967750 reassembly | 3300007752 | JGI/IMG-ER |
| Human anterior nares microbial communities from NIH, USA - visit 2, subject 158479027 reassembly | 3300007761 | JGI/IMG-ER |
| Human anterior nares microbial communities from NIH, USA - visit 1, subject 763496533 reassembly | 3300007762 | JGI/IMG-ER |
| Human anterior nares microbial communities from NIH, USA - visit 1, subject 763961826 reassembly | 3300007780 | JGI/IMG-ER |
| Human anterior nares microbial communities from NIH, USA - visit 1, subject 823052294 reassembly | 3300007830 | JGI/IMG-ER |
| Human anterior nares microbial communities from NIH, USA - visit 2, subject 764447348 reassembly | 3300007842 | JGI/IMG-ER |
| Human anterior nares microbial communities from NIH, USA - visit 1, subject 160400887 reassembly | 3300007868 | JGI/IMG-ER |
| Human anterior nares microbial communities from NIH, USA - visit 1, subject 159591683 reassembly | 3300007875 | JGI/IMG-ER |
| Human anterior nares microbial communities from NIH, USA - visit 2, subject 764892411 reassembly | 3300007877 | JGI/IMG-ER |
| Human anterior nares microbial communities from NIH, USA - visit 2, subject 763961826 replicate 1 reassembly | 3300007879 | JGI/IMG-ER |
| Human anterior nares microbial communities from NIH, USA - visit 1, subject 158398106 reassembly | 3300007883 | JGI/IMG-ER |
| Human anterior nares microbial communities from NIH, USA - visit 1, subject 159571453 reassembly | 3300007886 | JGI/IMG-ER |
| Human anterior nares microbial communities from NIH, USA - visit 2, subject 765094712 reassembly | 3300007889 | JGI/IMG-ER |
| Human anterior nares microbial communities from NIH, USA - visit 1, subject 764083206 replicate 2 reassembly | 3300007894 | JGI/IMG-ER |
| Human anterior nares microbial communities from NIH, USA - visit 1, subject 765135172 reassembly | 3300007898 | JGI/IMG-ER |
| Human anterior nares microbial communities from NIH, USA - visit 1, subject 159551223 reassembly | 3300007905 | JGI/IMG-ER |
| Human anterior nares microbial communities from NIH, USA - visit number 3 of subject 159510762 reassembly | 3300007911 | JGI/IMG-ER |
| Human anterior nares microbial communities from NIH, USA - visit 2, subject 763435843 reassembly | 3300007912 | JGI/IMG-ER |
| Human anterior nares microbial communities from NIH, USA - visit 2, subject 764083206 replicate 2 reassembly | 3300007913 | JGI/IMG-ER |
| Human anterior nares microbial communities from NIH, USA - visit 1, subject 764447348 reassembly | 3300007922 | JGI/IMG-ER |
| Human anterior nares microbial communities from NIH, USA - visit 1, subject 763982056 reassembly | 3300007928 | JGI/IMG-ER |
| Human anterior nares microbial communities from NIH, USA - visit 1, subject 764508039 reassembly | 3300007930 | JGI/IMG-ER |
| Human anterior nares microbial communities from NIH, USA - visit 2, subject 159591683 reassembly | 3300007933 | JGI/IMG-ER |
| Human anterior nares microbial communities from NIH, USA - visit 1, subject 160603188 reassembly | 3300007936 | JGI/IMG-ER |
| Human anterior nares microbial communities from NIH, USA - visit 2, subject 159814214 reassembly | 3300007942 | JGI/IMG-ER |
| Human anterior nares microbial communities from NIH, USA - visit 1, subject 764325968 reassembly | 3300007943 | JGI/IMG-ER |
| Human anterior nares microbial communities from NIH, USA - visit 2, subject 764487809 reassembly | 3300007947 | JGI/IMG-ER |
| Human anterior nares microbial communities from NIH, USA - visit 1, subject 160380657 reassembly | 3300007948 | JGI/IMG-ER |
| Human anterior nares microbial communities from NIH, USA - visit 1, subject 765560005 reassembly | 3300008284 | JGI/IMG-ER |
| Human anterior nares microbial communities from NIH, USA - visit 1, subject 159207311 reassembly | 3300008292 | JGI/IMG-ER |
| Human anterior nares microbial communities from NIH, USA - visit 1, subject 765094712 reassembly | 3300008303 | JGI/IMG-ER |
| Human anterior nares microbial communities from NIH, USA - visit 2, subject 159490532 reassembly | 3300008349 | JGI/IMG-ER |
| Human anterior nares microbial communities from NIH, USA - visit 1, subject 404239096 reassembly | 3300008384 | JGI/IMG-ER |
| Human anterior nares microbial communities from NIH, USA - visit 2, subject 764305738 reassembly | 3300008407 | JGI/IMG-ER |
| Human anterior nares microbial communities from NIH, USA - visit 2, subject 159369152 reassembly | 3300008475 | JGI/IMG-ER |
| Human anterior nares microbial communities from NIH, USA - visit 2, subject 763496533 reassembly | 3300008477 | JGI/IMG-ER |
| Human anterior nares microbial communities from NIH, USA - visit 1, subject 765074482 reassembly | 3300008484 | JGI/IMG-ER |
| Human posterior fornix microbial communities from NIH, USA - visit 1, subject 765640925 | 3300006200 | JGI/IMG-ER |
| Human posterior fornix microbial communities from NIH, USA - visit 2, subject 764143897 | 3300006209 | JGI/IMG-ER |
| Human posterior fornix microbial communities from NIH, USA - visit 2, subject 763820215 | 3300006212 | JGI/IMG-ER |
| Human posterior fornix microbial communities from NIH, USA - visit 1, subject 338793263 | 3300006238 | JGI/IMG-ER |
| Human posterior fornix microbial communities from NIH, USA - visit 1, subject 763577454 | 3300006239 | JGI/IMG-ER |
| Human posterior fornix microbial communities from NIH, USA - visit 2 of subject 158337416 | 3300006272 | JGI/IMG-ER |
| Human posterior fornix microbial communities from NIH, USA - visit 3 of subject 158883629 | 3300006406 | JGI/IMG-ER |
| Human posterior fornix microbial communities from NIH, USA - visit 1, subject 763840445 | 3300006485 | JGI/IMG-ER |
| Human posterior fornix microbial communities from NIH, USA - visit 1, subject 763901136 | 3300006493 | JGI/IMG-ER |
| Human posterior fornix microbial communities from NIH, USA - visit 2, subject 370425937 | 3300006510 | JGI/IMG-ER |
| Human posterior fornix microbial communities from NIH, USA - visit 2, subject 763840445 | 3300006560 | JGI/IMG-ER |
| Human posterior fornix microbial communities from NIH, USA - visit 2, subject 159227541 | 3300006723 | JGI/IMG-ER |
| Human posterior fornix microbial communities from NIH, USA - visit 2, subject 158256496 | 3300006725 | JGI/IMG-ER |
| Human posterior fornix microbial communities from NIH, USA - visit 1, subject 370425937 | 3300006740 | JGI/IMG-ER |
| Human posterior fornix microbial communities from NIH, USA - visit 1, subject 508703490 | 3300006811 | JGI/IMG-ER |
| Human posterior fornix microbial communities from NIH, USA - visit 1, subject 638754422 | 3300006877 | JGI/IMG-ER |
| Human posterior fornix microbial communities from NIH, USA - visit 1, subject 765337473 | 3300006975 | JGI/IMG-ER |
| Human posterior fornix microbial communities from NIH, USA - visit 1, subject 737052003 | 3300006977 | JGI/IMG-ER |
| Human posterior fornix microbial communities from NIH, USA - visit 1, subject 159227541 | 3300006979 | JGI/IMG-ER |
| Human posterior fornix microbial communities from NIH, USA - visit 1, subject 764042746 | 3300007001 | JGI/IMG-ER |
| Human posterior fornix microbial communities from NIH, USA - visit 1, subject 764143897 | 3300007002 | JGI/IMG-ER |
| Human posterior fornix microbial communities from NIH, USA - visit 2, subject 764042746 | 3300007010 | JGI/IMG-ER |
| Human posterior fornix microbial communities from NIH, USA - visit 1, subject 158458797 | 3300007035 | JGI/IMG-ER |
| Human posterior fornix microbial communities from NIH, USA - visit 2, subject 159005010 reassembly | 3300007272 | JGI/IMG-ER |
| Human posterior fornix microbial communities from NIH, USA - visit 1, subject 158944319 reassembly | 3300007303 | JGI/IMG-ER |
| Human posterior fornix microbial communities from NIH, USA - visit 1, subject 809635352 reassembly | 3300007364 | JGI/IMG-ER |
| Human posterior fornix microbial communities from NIH, USA - visit 2, subject 809635352 reassembly | 3300007738 | JGI/IMG-ER |
| Human posterior fornix microbial communities from NIH, USA - visit 1, subject 160582958 reassembly | 3300007826 | JGI/IMG-ER |
| Human posterior fornix microbial communities from NIH, USA - visit 1, subject 764649650 reassembly | 3300007827 | JGI/IMG-ER |
| Human posterior fornix microbial communities from NIH, USA - visit 1, subject 159915365 reassembly | 3300007829 | JGI/IMG-ER |
| Human posterior fornix microbial communities from NIH, USA - visit 1, subject 765013792 reassembly | 3300007838 | JGI/IMG-ER |
| Human posterior fornix microbial communities from NIH, USA - visit 1, subject 160319967 reassembly | 3300007843 | JGI/IMG-ER |
| Human posterior fornix microbial communities from NIH, USA - visit 1, subject 160704339 reassembly | 3300007856 | JGI/IMG-ER |
| Human posterior fornix microbial communities from NIH, USA - visit 1, subject 158742018 reassembly | 3300007857 | JGI/IMG-ER |
| Human posterior fornix microbial communities from NIH, USA - visit 2, subject 764062976 reassembly | 3300007870 | JGI/IMG-ER |
| Human posterior fornix microbial communities from NIH, USA - visit 2, subject 159713063 reassembly | 3300007874 | JGI/IMG-ER |
| Human posterior fornix microbial communities from NIH, USA - visit 1, subject 160643649 reassembly | 3300007880 | JGI/IMG-ER |
| Human posterior fornix microbial communities from NIH, USA - visit 2, subject 158883629 reassembly | 3300007882 | JGI/IMG-ER |
| Human posterior fornix microbial communities from NIH, USA - visit 1, subject 764588959 reassembly | 3300007884 | JGI/IMG-ER |
| Human posterior fornix microbial communities from NIH, USA - visit 2, subject 763577454 reassembly | 3300007892 | JGI/IMG-ER |
| Human posterior fornix microbial communities from NIH, USA - visit number 3 of subject 159753524 reassembly | 3300007895 | JGI/IMG-ER |
| Human posterior fornix microbial communities from NIH, USA - visit 1, subject 159247771 reassembly | 3300007903 | JGI/IMG-ER |
| Human posterior fornix microbial communities from NIH, USA - visit 2, subject 246515023 reassembly | 3300007904 | JGI/IMG-ER |
| Human posterior fornix microbial communities from NIH, USA - visit 1, subject 763759525 reassembly | 3300007916 | JGI/IMG-ER |
| Human posterior fornix microbial communities from NIH, USA - visit 1, subject 765620695 reassembly | 3300007927 | JGI/IMG-ER |
| Human posterior fornix microbial communities from NIH, USA - visit 1, subject 160502038 reassembly | 3300007929 | JGI/IMG-ER |
| Human posterior fornix microbial communities from NIH, USA - visit 1, subject 550534656 reassembly | 3300007941 | JGI/IMG-ER |
| Human posterior fornix microbial communities from NIH, USA - visit 1, subject 706846339 reassembly | 3300007952 | JGI/IMG-ER |
| Human posterior fornix microbial communities from NIH, USA - visit 2, subject 763759525 reassembly | 3300008124 | JGI/IMG-ER |
| Human posterior fornix microbial communities from NIH, USA - visit 1, subject 158337416 reassembly | 3300008351 | JGI/IMG-ER |
| Human posterior fornix microbial communities from NIH, USA - visit 1, subject 159753524 replicate 1 reassembly | 3300008411 | JGI/IMG-ER |
| Human posterior fornix microbial communities from NIH, USA - visit 1, subject 246515023 reassembly | 3300008418 | JGI/IMG-ER |
| Human posterior fornix microbial communities from NIH, USA - visit 2, subject 764811490 reassembly | 3300008419 | JGI/IMG-ER |
| Human posterior fornix microbial communities from NIH, USA - visit 2, subject 159247771 reassembly | 3300008432 | JGI/IMG-ER |
| Human posterior fornix microbial communities from NIH, USA - visit 2, subject 158944319 reassembly | 3300008476 | JGI/IMG-ER |
| Human posterior fornix microbial communities from NIH, USA - visit 1, subject 159753524 replicate 2 reassembly | 3300008501 | JGI/IMG-ER |
| Human posterior fornix microbial communities from NIH, USA - visit 1, subject 763820215 reassembly | 3300008623 | JGI/IMG-ER |
| Human posterior fornix microbial communities from NIH, USA - visit 2, subject 159753524 reassembly | 3300008678 | JGI/IMG-ER |
| Human posterior fornix microbial communities from NIH, USA - visit 1, subject 675950834 reassembly | 3300008711 | JGI/IMG-ER |
| Human posterior fornix microbial communities from NIH, USA - visit 2, subject 763901136 reassembly | 3300008719 | JGI/IMG-ER |
| Human Mid vagina microbial communities from NIH, USA - visit 2, subject 763577454 reassembly | 3300007891 | JGI/IMG-ER |
| Human Mid vagina microbial communities from NIH, USA - visit 1, subject 763577454 reassembly | 3300007939 | JGI/IMG-ER |
| Human Vaginal introitus microbial communities from NIH, USA - visit 1, subject 763577454 | 3300006991 | JGI/IMG-ER |
| Human Vaginal introitus microbial communities from NIH, USA - visit 2, subject 764042746 reassembly | 3300007809 | JGI/IMG-ER |
| Human Vaginal introitus microbial communities from NIH, USA - visit 2, subject 763577454 reassembly | 3300007893 | JGI/IMG-ER |
| Human buccal mucosa microbial communities from NIH, USA - visit 1, subject 246515023 | 3300006214 | JGI/IMG-ER |
| Human buccal mucosa microbial communities from NIH, USA - visit 1, subject 764588959 | 3300006242 | JGI/IMG-ER |
| Human buccal mucosa microbial communities from NIH, USA - visit 2, subject 370425937 | 3300006243 | JGI/IMG-ER |
| Human buccal mucosa microbial communities from NIH, USA - visit 2, subject 764811490 | 3300006244 | JGI/IMG-ER |
| Human buccal mucosa microbial communities from NIH, USA - visit 2, subject 159005010 | 3300006246 | JGI/IMG-ER |
| Human buccal mucosa microbial communities from NIH, USA - visit 1, subject 809635352 | 3300006284 | JGI/IMG-ER |
| Human buccal mucosa microbial communities from NIH, USA - visit 2 of subject 158883629 | 3300006475 | JGI/IMG-ER |
| Human buccal mucosa microbial communities from NIH, USA - visit 2, subject 763577454 | 3300006520 | JGI/IMG-ER |
| Human buccal mucosa microbial communities from NIH, USA - visit 1, subject 159753524 replicate 2 | 3300006521 | JGI/IMG-ER |
| Human buccal mucosa microbial communities from NIH, USA - visit 1, subject 508703490 | 3300006897 | JGI/IMG-ER |
| Human buccal mucosa microbial communities from NIH, USA - visit 1, subject 158337416 | 3300006980 | JGI/IMG-ER |
| Human buccal mucosa microbial communities from NIH, USA - visit 1, subject 370425937 | 3300006996 | JGI/IMG-ER |
| Human buccal mucosa microbial communities from NIH, USA - visit 1, subject 158458797 | 3300007024 | JGI/IMG-ER |
| Human buccal mucosa microbial communities from NIH, USA - visit 1, subject 764042746 | 3300007034 | JGI/IMG-ER |
| Human buccal mucosa microbial communities from NIH, USA - visit 1, subject 160643649 | 3300007047 | JGI/IMG-ER |
| Human buccal mucosa microbial communities from NIH, USA - visit 2, subject 158944319 | 3300007087 | JGI/IMG-ER |
| Human buccal mucosa microbial communities from NIH, USA - visit 2, subject 158337416 reassembly | 3300007259 | JGI/IMG-ER |
| Human buccal mucosa microbial communities from NIH, USA - visit 2, subject 763840445 reassembly | 3300007268 | JGI/IMG-ER |
| Human buccal mucosa microbial communities from NIH, USA - visit 1, subject 160319967 reassembly | 3300007278 | JGI/IMG-ER |
| Human buccal mucosa microbial communities from NIH, USA - visit 1, subject 158742018 reassembly | 3300007287 | JGI/IMG-ER |
| Human buccal mucosa microbial communities from NIH, USA - visit 2, subject 159247771 reassembly | 3300007309 | JGI/IMG-ER |
| Human buccal mucosa microbial communities from NIH, USA - visit 1, subject 763840445 reassembly | 3300007366 | JGI/IMG-ER |
| Human buccal mucosa microbial communities from NIH, USA - visit 2, subject 763820215 reassembly | 3300007367 | JGI/IMG-ER |
| Human buccal mucosa microbial communities from NIH, USA - visit 1, subject 550534656 reassembly | 3300007529 | JGI/IMG-ER |
| Human buccal mucosa microbial communities from NIH, USA - visit 1, subject 159915365 reassembly | 3300007660 | JGI/IMG-ER |
| Human buccal mucosa microbial communities from NIH, USA - visit 1, subject 160502038 reassembly | 3300007779 | JGI/IMG-ER |
| Human buccal mucosa microbial communities from NIH, USA - visit 2, subject 809635352 reassembly | 3300007839 | JGI/IMG-ER |
| Human buccal mucosa microbial communities from NIH, USA - visit 1, subject 765034022 reassembly | 3300007841 | JGI/IMG-ER |
| Human buccal mucosa microbial communities from NIH, USA - visit 1, subject 763901136 reassembly | 3300007854 | JGI/IMG-ER |
| Human buccal mucosa microbial communities from NIH, USA - visit 1, subject 160704339 reassembly | 3300007859 | JGI/IMG-ER |
| Human buccal mucosa microbial communities from NIH, USA - visit number 3 of subject 158883629 reassembly | 3300007897 | JGI/IMG-ER |
| Human buccal mucosa microbial communities from NIH, USA - visit number 3 of subject 159753524 reassembly | 3300007915 | JGI/IMG-ER |
| Human buccal mucosa microbial communities from NIH, USA - visit 2, subject 763901136 reassembly | 3300007926 | JGI/IMG-ER |
| Human buccal mucosa microbial communities from NIH, USA - visit 2, subject 158256496 reassembly | 3300007932 | JGI/IMG-ER |
| Human buccal mucosa microbial communities from NIH, USA - visit 1, subject 160582958 reassembly | 3300007967 | JGI/IMG-ER |
| Human buccal mucosa microbial communities from NIH, USA - visit 2, subject 638754422 reassembly | 3300008056 | JGI/IMG-ER |
| Human buccal mucosa microbial communities from NIH, USA - visit 2, subject 159713063 reassembly | 3300008068 | JGI/IMG-ER |
| Human buccal mucosa microbial communities from NIH, USA - visit 2, subject 764143897 reassembly | 3300008074 | JGI/IMG-ER |
| Human buccal mucosa microbial communities from NIH, USA - visit 1, subject 765337473 reassembly | 3300008127 | JGI/IMG-ER |
| Human buccal mucosa microbial communities from NIH, USA - visit 1, subject 763820215 reassembly | 3300008277 | JGI/IMG-ER |
| Human buccal mucosa microbial communities from NIH, USA - visit 1, subject 338793263 reassembly | 3300008302 | JGI/IMG-ER |
| Human buccal mucosa microbial communities from NIH, USA - visit 1, subject 638754422 reassembly | 3300008336 | JGI/IMG-ER |
| Human buccal mucosa microbial communities from NIH, USA - visit 2, subject 246515023 reassembly | 3300008382 | JGI/IMG-ER |
| Human buccal mucosa microbial communities from NIH, USA - visit 1, subject 765620695 reassembly | 3300008413 | JGI/IMG-ER |
| Human buccal mucosa microbial communities from NIH, USA - visit 1, subject 765013792 reassembly | 3300008415 | JGI/IMG-ER |
| Human buccal mucosa microbial communities from NIH, USA - visit 2, subject 159753524 reassembly | 3300008433 | JGI/IMG-ER |
| Human buccal mucosa microbial communities from NIH, USA - visit 2, subject 763759525 reassembly | 3300008471 | JGI/IMG-ER |
| Human buccal mucosa microbial communities from NIH, USA - visit 1, subject 737052003 reassembly | 3300008473 | JGI/IMG-ER |
| Human buccal mucosa microbial communities from NIH, USA - visit 1, subject 158944319 reassembly | 3300008474 | JGI/IMG-ER |
| Human buccal mucosa microbial communities from NIH, USA - visit 2, subject 764062976 reassembly | 3300008500 | JGI/IMG-ER |
| Human buccal mucosa microbial communities from NIH, USA - visit 1, subject 675950834 reassembly | 3300008615 | JGI/IMG-ER |
| Human buccal mucosa microbial communities from NIH, USA - visit 1, subject 706846339 reassembly | 3300008643 | JGI/IMG-ER |
| Human buccal mucosa microbial communities from NIH, USA - visit 1, subject 763577454 reassembly | 3300008708 | JGI/IMG-ER |
| Human buccal mucosa microbial communities from NIH, USA - visit 1, subject 159753524 replicate 1 reassembly | 3300008720 | JGI/IMG-ER |
| Human buccal mucosa microbial communities from NIH, USA - visit 1, subject 763759525 reassembly | 3300008749 | JGI/IMG-ER |
| Human buccal mucosa microbial communities from NIH, USA - visit 1, subject 764649650 reassembly | 3300008751 | JGI/IMG-ER |
| Human buccal mucosa microbial communities from NIH, USA - visit 1, subject 765135172 | 3300006286 | JGI/IMG-ER |
| Human buccal mucosa microbial communities from NIH, USA - visit 2 of subject 764325968 | 3300006287 | JGI/IMG-ER |
| Human buccal mucosa microbial communities from NIH, USA - visit 2, subject 604812005 | 3300006291 | JGI/IMG-ER |
| Human buccal mucosa microbial communities from NIH, USA - visit 1, subject 160603188 | 3300006297 | JGI/IMG-ER |
| Human buccal mucosa microbial communities from NIH, USA - visit 2 of subject 764224817 | 3300006301 | JGI/IMG-ER |
| Human buccal mucosa microbial communities from NIH, USA - visit 1, subject 159814214 | 3300006302 | JGI/IMG-ER |
| Human buccal mucosa microbial communities from NIH, USA - visit 2 of subject 158802708 | 3300006407 | JGI/IMG-ER |
| Human buccal mucosa microbial communities from NIH, USA - visit 2, subject 764487809 | 3300006489 | JGI/IMG-ER |
| Human buccal mucosa microbial communities from NIH, USA - visit 1, subject 823052294 | 3300006545 | JGI/IMG-ER |
| Human buccal mucosa microbial communities from NIH, USA - visit 1, subject 764285508 | 3300006563 | JGI/IMG-ER |
| Human buccal mucosa microbial communities from NIH, USA - visit 1, subject 160158126 | 3300006742 | JGI/IMG-ER |
| Human buccal mucosa microbial communities from NIH, USA - visit 1, subject 861967750 | 3300006836 | JGI/IMG-ER |
| Human buccal mucosa microbial communities from NIH, USA - visit 1, subject 160400887 | 3300006898 | JGI/IMG-ER |
| Human buccal mucosa microbial communities from NIH, USA - visit 1, subject 764305738 replicate 1 | 3300007039 | JGI/IMG-ER |
| Human buccal mucosa microbial communities from NIH, USA - visit 1, subject 159207311 | 3300007065 | JGI/IMG-ER |
| Human buccal mucosa microbial communities from NIH, USA - visit 1, subject 763435843 | 3300007127 | JGI/IMG-ER |
| Human buccal mucosa microbial communities from NIH, USA - visit number 3 of subject 763536994 | 3300007132 | JGI/IMG-ER |
| Human buccal mucosa microbial communities from NIH, USA - visit 1, subject 159551223 reassembly | 3300007262 | JGI/IMG-ER |
| Human buccal mucosa microbial communities from NIH, USA - visit 1, subject 159490532 reassembly | 3300007284 | JGI/IMG-ER |
| Human buccal mucosa microbial communities from NIH, USA - visit 2, subject 764892411 reassembly | 3300007306 | JGI/IMG-ER |
| Human buccal mucosa microbial communities from NIH, USA - visit 1, subject 404239096 reassembly | 3300007497 | JGI/IMG-ER |
| Human buccal mucosa microbial communities from NIH, USA - visit 2, subject 160158126 reassembly | 3300007659 | JGI/IMG-ER |
| Human buccal mucosa microbial communities from NIH, USA - visit 1, subject 765701615 reassembly | 3300007663 | JGI/IMG-ER |
| Human buccal mucosa microbial communities from NIH, USA - visit 1, subject 763982056 reassembly | 3300007773 | JGI/IMG-ER |
| Human buccal mucosa microbial communities from NIH, USA - visit 1, subject 159591683 reassembly | 3300007782 | JGI/IMG-ER |
| Human buccal mucosa microbial communities from NIH, USA - visit 1, subject 159611913 reassembly | 3300007787 | JGI/IMG-ER |
| Human buccal mucosa microbial communities from NIH, USA - visit 2, subject 159490532 reassembly | 3300007789 | JGI/IMG-ER |
| Human buccal mucosa microbial communities from NIH, USA - visit 2, subject 159207311 reassembly | 3300007792 | JGI/IMG-ER |
| Human buccal mucosa microbial communities from NIH, USA - visit 1, subject 763961826 reassembly | 3300007810 | JGI/IMG-ER |
| Human buccal mucosa microbial communities from NIH, USA - visit 1, subject 764305738 replicate 2 reassembly | 3300007825 | JGI/IMG-ER |
| Human buccal mucosa microbial communities from NIH, USA - visit 2, subject 686765762 reassembly | 3300007844 | JGI/IMG-ER |
| Human buccal mucosa microbial communities from NIH, USA - visit 1, subject 159632143 reassembly | 3300007867 | JGI/IMG-ER |
| Human buccal mucosa microbial communities from NIH, USA - visit 2, subject 764305738 reassembly | 3300007869 | JGI/IMG-ER |
| Human buccal mucosa microbial communities from NIH, USA - visit 1, subject 765094712 reassembly | 3300007887 | JGI/IMG-ER |
| Human buccal mucosa microbial communities from NIH, USA - visit 1, subject 764487809 reassembly | 3300007888 | JGI/IMG-ER |
| Human buccal mucosa microbial communities from NIH, USA - visit 1, subject 159571453 reassembly | 3300007919 | JGI/IMG-ER |
| Human buccal mucosa microbial communities from NIH, USA - visit 1, subject 764508039 reassembly | 3300007931 | JGI/IMG-ER |
| Human buccal mucosa microbial communities from NIH, USA - visit 2, subject 159611913 reassembly | 3300007935 | JGI/IMG-ER |
| Human buccal mucosa microbial communities from NIH, USA - visit 1, subject 604812005 reassembly | 3300007937 | JGI/IMG-ER |
| Human buccal mucosa microbial communities from NIH, USA - visit 2, subject 158479027 reassembly | 3300007938 | JGI/IMG-ER |
| Human buccal mucosa microbial communities from NIH, USA - visit 1, subject 158499257 reassembly | 3300007951 | JGI/IMG-ER |
| Human buccal mucosa microbial communities from NIH, USA - visit 1, subject 764447348 reassembly | 3300007968 | JGI/IMG-ER |
| Human buccal mucosa microbial communities from NIH, USA - visit 1, subject 160380657 reassembly | 3300007995 | JGI/IMG-ER |
| Human buccal mucosa microbial communities from NIH, USA - visit 1, subject 765560005 reassembly | 3300008057 | JGI/IMG-ER |
| Human buccal mucosa microbial communities from NIH, USA - visit 2, subject 159571453 reassembly | 3300008061 | JGI/IMG-ER |
| Human buccal mucosa microbial communities from NIH, USA - visit 2, subject 158499257 reassembly | 3300008104 | JGI/IMG-ER |
| Human buccal mucosa microbial communities from NIH, USA - visit 2, subject 159814214 reassembly | 3300008130 | JGI/IMG-ER |
| Human buccal mucosa microbial communities from NIH, USA - visit 2, subject 763961826 reassembly | 3300008151 | JGI/IMG-ER |
| Human buccal mucosa microbial communities from NIH, USA - visit 2, subject 159369152 reassembly | 3300008270 | JGI/IMG-ER |
| Human buccal mucosa microbial communities from NIH, USA - visit 1, subject 160218816 reassembly | 3300008281 | JGI/IMG-ER |
| Human buccal mucosa microbial communities from NIH, USA - visit 1, subject 765074482 reassembly | 3300008307 | JGI/IMG-ER |
| Human buccal mucosa microbial communities from NIH, USA - visit 1, subject 159268001 reassembly | 3300008330 | JGI/IMG-ER |
| Human buccal mucosa microbial communities from NIH, USA - visit 1, subject 764224817 reassembly | 3300008331 | JGI/IMG-ER |
| Human buccal mucosa microbial communities from NIH, USA - visit 1, subject 763860675 reassembly | 3300008338 | JGI/IMG-ER |
| Human buccal mucosa microbial communities from NIH, USA - visit 2, subject 159268001 reassembly | 3300008348 | JGI/IMG-ER |
| Human buccal mucosa microbial communities from NIH, USA - visit 1, subject 686765762 reassembly | 3300008414 | JGI/IMG-ER |
| Human buccal mucosa microbial communities from NIH, USA - visit 2, subject 159591683 reassembly | 3300008431 | JGI/IMG-ER |
| Human buccal mucosa microbial communities from NIH, USA - visit 2, subject 764083206 reassembly | 3300008483 | JGI/IMG-ER |
| Human buccal mucosa microbial communities from NIH, USA - visit 1, subject 160178356 reassembly | 3300008498 | JGI/IMG-ER |
| Human buccal mucosa microbial communities from NIH, USA - visit 1, subject 160765029 reassembly | 3300008511 | JGI/IMG-ER |
| Human buccal mucosa microbial communities from NIH, USA - visit 2, subject 159551223 reassembly | 3300008604 | JGI/IMG-ER |
| Human buccal mucosa microbial communities from NIH, USA - visit 2, subject 763435843 reassembly | 3300008618 | JGI/IMG-ER |
| Human buccal mucosa microbial communities from NIH, USA - visit 1, subject 763496533 reassembly | 3300008630 | JGI/IMG-ER |
| Human buccal mucosa microbial communities from NIH, USA - visit 1, subject 764083206 reassembly | 3300008637 | JGI/IMG-ER |
| Human buccal mucosa microbial communities from NIH, USA - visit 2, subject 765094712 reassembly | 3300008654 | JGI/IMG-ER |
| Human buccal mucosa microbial communities from NIH, USA - visit 2, subject 763496533 reassembly | 3300008756 | JGI/IMG-ER |
| Human attached/keratinized gingiva microbial communities from NIH, USA - visit 1, subject 763496533 | 3300007285 | JGI/IMG-ER |
| Human attached/keratinized gingiva microbial communities from NIH, USA - visit 1, subject 763961826 reassembly | 3300007786 | JGI/IMG-ER |
| Human attached/keratinized gingiva microbial communities from NIH, USA - visit 2, subject 763961826 reassembly | 3300007828 | JGI/IMG-ER |
| Human attached/keratinized gingiva microbial communities from NIH, USA - visit 2, subject 763496533 reassembly | 3300007940 | JGI/IMG-ER |
| Human attached/keratinized gingiva microbial communities from NIH, USA - visit 2, subject 763577454 reassembly | 3300008495 | JGI/IMG-ER |
| Human attached/keratinized gingiva microbial communities from NIH, USA - visit 1, subject 763577454 reassembly | 3300008714 | JGI/IMG-ER |
| Human hard palate microbial communities from NIH, USA - visit 2, subject 765560005 reassembly | 3300007800 | JGI/IMG-ER |
| Human palatine tonsils microbial communities from NIH, USA - visit 2, subject 764042746 reassembly | 3300007652 | JGI/IMG-ER |
| Human palatine tonsils microbial communities from NIH, USA - visit 2, subject 763961826 reassembly | 3300008154 | JGI/IMG-ER |
| Human palatine tonsils microbial communities from NIH, USA - visit 2, subject 763577454 reassembly | 3300008494 | JGI/IMG-ER |
| Human palatine tonsils microbial communities from NIH, USA - visit 1, subject 763496533 reassembly | 3300008628 | JGI/IMG-ER |
| Human palatine tonsils microbial communities from NIH, USA - visit 1, subject 763577454 reassembly | 3300008707 | JGI/IMG-ER |
| Human palatine tonsils microbial communities from NIH, USA - visit 2, subject 763496533 reassembly | 3300008754 | JGI/IMG-ER |
| Human saliva microbial communities from NIH, USA - visit 2, subject 763577454 | 3300006496 | JGI/IMG-ER |
| Human saliva microbial communities from NIH, USA - visit 1, subject 763577454 | 3300007086 | JGI/IMG-ER |
| Human saliva microbial communities from NIH, USA - visit 1, subject 763961826 | 3300007196 | JGI/IMG-ER |
| Human saliva microbial communities from NIH, USA - visit 2, subject 763496533 reassembly | 3300007971 | JGI/IMG-ER |
| Human saliva microbial communities from NIH, USA - visit 1, subject 763496533 reassembly | 3300008632 | JGI/IMG-ER |
| Human subgingival plaque microbial communities from NIH, USA - visit 2, subject 764042746 | 3300006307 | JGI/IMG-ER |
| Human subgingival plaque microbial communities from NIH, USA - visit 1, subject 763435843 | 3300007145 | JGI/IMG-ER |
| Human subgingival plaque microbial communities from NIH, USA - visit 1, subject 763961826 | 3300007206 | JGI/IMG-ER |
| Human subgingival plaque microbial communities from NIH, USA - visit 2, subject 763577454 reassembly | 3300007525 | JGI/IMG-ER |
| Human subgingival plaque microbial communities from NIH, USA - visit 1, subject 763496533 reassembly | 3300007527 | JGI/IMG-ER |
| Human subgingival plaque microbial communities from NIH, USA - visit 2, subject 763961826 reassembly | 3300008139 | JGI/IMG-ER |
| Human subgingival plaque microbial communities from NIH, USA - visit 2, subject 763496533 reassembly | 3300008160 | JGI/IMG-ER |
| Human subgingival plaque microbial communities from NIH, USA - visit 1, subject 763577454 reassembly | 3300008614 | JGI/IMG-ER |
| Human throat microbial communities from NIH, USA - visit 2, subject 765560005 reassembly | 3300007662 | JGI/IMG-ER |
| Human throat microbial communities from NIH, USA - visit 1, subject 763961826 reassembly | 3300007783 | JGI/IMG-ER |
| Human throat microbial communities from NIH, USA - visit 1, subject 763496533 reassembly | 3300007978 | JGI/IMG-ER |
| Human throat microbial communities from NIH, USA - visit 2, subject 763961826 reassembly | 3300008148 | JGI/IMG-ER |
| Human throat microbial communities from NIH, USA - visit 2, subject 763577454 reassembly | 3300008492 | JGI/IMG-ER |
| Human throat microbial communities from NIH, USA - visit 1, subject 763577454 reassembly | 3300008616 | JGI/IMG-ER |
| Human throat microbial communities from NIH, USA - visit 2, subject 763496533 reassembly | 3300008755 | JGI/IMG-ER |
| Human retroauricular crease microbial communities from NIH, USA - visit 1, subject 338793263 reassembly | 3300008657 | JGI/IMG-ER |
| Human retroauricular crease microbial communities from NIH, USA - visit 1, subject 764042746 reassembly | 3300008731 | JGI/IMG-ER |
| Human left retroauricular crease microbial communities from NIH, USA - visit 2 of subject 763961826 | 3300006441 | JGI/IMG-ER |
| Human left retroauricular crease microbial communities from NIH, USA - visit 2, subject 764083206 reassembly | 3300007950 | JGI/IMG-ER |
| Human retroauricular crease microbial communities from NIH, USA - visit 1, subject 159450072 reassembly | 3300007866 | JGI/IMG-ER |
| Human retroauricular crease microbial communities from NIH, USA - visit 1, subject 159591683 replicate 1 | 3300006486 | JGI/IMG-ER |
| Human retroauricular crease microbial communities from NIH, USA - visit 1, subject 159591683 replicate 2 reassembly | 3300008668 | JGI/IMG-ER |
| Human retroauricular crease microbial communities from NIH, USA - visit 1, subject 16040087 replicate 1 | 3300006288 | JGI/IMG-ER |
| Human retroauricular crease microbial communities from NIH, USA - visit 1, subject 16040088 replicate 2 | 3300007054 | JGI/IMG-ER |
| Human retroauricular crease microbial communities from NIH, USA - visit 1, subject 160603188 replicate 1 reassembly | 3300008325 | JGI/IMG-ER |
| Human retroauricular crease microbial communities from NIH, USA - visit 1, subject 160603188 replicate 2 | 3300007131 | JGI/IMG-ER |
| Human retroauricular crease microbial communities from NIH, USA - visit 2, subject 159490532 reassembly | 3300008617 | JGI/IMG-ER |
| Human retroauricular crease microbial communities from NIH, USA - visit 2, subject 159571453 reassembly | 3300007270 | JGI/IMG-ER |
| Human retroauricular crease microbial communities from NIH, USA - visit 2, subject 159591683 replicate 1 reassembly | 3300007307 | JGI/IMG-ER |
| Human retroauricular crease microbial communities from NIH, USA - visit 2, subject 159591683 replicate 2 reassembly | 3300007311 | JGI/IMG-ER |
| Human retroauricular crease microbial communities from NIH, USA - visit 2, subject 15961191 | 3300006959 | JGI/IMG-ER |
| Human retroauricular crease microbial communities from NIH, USA - visit 2, subject 763496533 reassembly | 3300008327 | JGI/IMG-ER |
| Human retroauricular crease microbial communities from NIH, USA - visit 2, subject 764224817 reassembly | 3300007785 | JGI/IMG-ER |
| Human retroauricular crease microbial communities from NIH, USA - visit 2, subject 764305738 | 3300007040 | JGI/IMG-ER |
| Human retroauricular crease microbial communities from NIH, USA - visit 2, subject 764447348 reassembly | 3300007310 | JGI/IMG-ER |
| Human retroauricular crease microbial communities from NIH, USA - visit 2, subject 765094712 reassembly | 3300008718 | JGI/IMG-ER |
| Human retroauricular creasemicrobial communities from NIH, USA - visit 1, subject 404239096 | 3300006472 | JGI/IMG-ER |
| Human right retroauricular crease microbial communities from NIH, USA - visit 2 of subject 763961826 replicate 2 | 3300006456 | JGI/IMG-ER |
| Human right retroauricular crease microbial communities from NIH, USA - visit 2 of subject 764083206 replicate 2 | 3300006677 | JGI/IMG-ER |
| Human right retroauricular crease microbial communities from NIH, USA - visit 2, subject 15961191 replicate 2 reassembly | 3300007781 | JGI/IMG-ER |
| Human right retroauricular crease microbial communities from NIH, USA - visit 2, subject 763961826 replicate 1 reassembly | 3300008593 | JGI/IMG-ER |
| Human right retroauricular crease microbial communities from NIH, USA - visit 2, subject 764083206 replicate 1 reassembly | 3300007949 | JGI/IMG-ER |
| Human tongue dorsum microbial communities from NIH, USA - visit 1, subject 706846339 | 3300006254 | JGI/IMG-ER |
| Human tongue dorsum microbial communities from NIH, USA - visit 1, subject 765034022 | 3300006259 | JGI/IMG-ER |
| Human tongue dorsum microbial communities from NIH, USA - visit 1, subject 159915365 | 3300006260 | JGI/IMG-ER |
| Human tongue dorsum microbial communities from NIH, USA - visit 1, subject 246515023 | 3300006262 | JGI/IMG-ER |
| Human tongue dorsum microbial communities from NIH, USA - visit 1, subject 809635352 | 3300006289 | JGI/IMG-ER |
| Human tongue dorsum microbial communities from NIH, USA - visit 2, subject 764062976 | 3300006319 | JGI/IMG-ER |
| Human tongue dorsum microbial communities from NIH, USA - visit 2, subject 763577454 | 3300006320 | JGI/IMG-ER |
| Human tongue dorsum microbial communities from NIH, USA - visit 2 of subject 764143897 | 3300006460 | JGI/IMG-ER |
| Human tongue dorsum microbial communities from NIH, USA - visit 2 of subject 158883629 | 3300006477 | JGI/IMG-ER |
| Human tongue dorsum microbial communities from NIH, USA - visit 2 of subject 159005010 | 3300006678 | JGI/IMG-ER |
| Human tongue dorsum microbial communities from NIH, USA - visit 2, subject 159227541 | 3300006748 | JGI/IMG-ER |
| Human tongue dorsum microbial communities from NIH, USA - visit 2, subject 809635352 | 3300007079 | JGI/IMG-ER |
| Human tongue dorsum microbial communities from NIH, USA - visit 1, subject 158944319 | 3300007107 | JGI/IMG-ER |
| Human tongue dorsum microbial communities from NIH, USA - visit 1, subject 675950834 | 3300007125 | JGI/IMG-ER |
| Human tongue dorsum microbial communities from NIH, USA - visit 2, subject 764042746 | 3300007167 | JGI/IMG-ER |
| Human tongue dorsum microbial communities from NIH, USA - visit 2, subject 246515023 | 3300007186 | JGI/IMG-ER |
| Human tongue dorsum microbial communities from NIH, USA - visit 1, subject 550534656 | 3300007194 | JGI/IMG-ER |
| Human tongue dorsum microbial communities from NIH, USA - visit 2, subject 763901136 replicate 1 | 3300007208 | JGI/IMG-ER |
| Human tongue dorsum microbial communities from NIH, USA - visit 2, subject 158337416 reassembly | 3300007297 | JGI/IMG-ER |
| Human tongue dorsum microbial communities from NIH, USA - visit 2, subject 159247771 reassembly | 3300007315 | JGI/IMG-ER |
| Human tongue dorsum microbial communities from NIH, USA - visit 1, subject 158742018 reassembly | 3300007318 | JGI/IMG-ER |
| Human tongue dorsum microbial communities from NIH, USA - visit 2, subject 763820215 reassembly | 3300007339 | JGI/IMG-ER |
| Human tongue dorsum microbial communities from NIH, USA - visit 1, subject 763840445 reassembly | 3300007566 | JGI/IMG-ER |
| Human tongue dorsum microbial communities from NIH, USA - visit 1, subject 764588959 reassembly | 3300007648 | JGI/IMG-ER |
| Human tongue dorsum microbial communities from NIH, USA - visit 1, subject 160502038 reassembly | 3300007711 | JGI/IMG-ER |
| Human tongue dorsum microbial communities from NIH, USA - visit 1, subject 158458797 reassembly | 3300007795 | JGI/IMG-ER |
| Human tongue dorsum microbial communities from NIH, USA - visit 1, subject 764143897 reassembly | 3300007924 | JGI/IMG-ER |
| Human tongue dorsum microbial communities from NIH, USA - visit 1, subject 763901136 replicate 2 reassembly | 3300007977 | JGI/IMG-ER |
| Human tongue dorsum microbial communities from NIH, USA - visit 1, subject 765620695 reassembly | 3300007996 | JGI/IMG-ER |
| Human tongue dorsum microbial communities from NIH, USA - visit 2, subject 763759525 reassembly | 3300008080 | JGI/IMG-ER |
| Human tongue dorsum microbial communities from NIH, USA - visit 1, subject 160643649 reassembly | 3300008090 | JGI/IMG-ER |
| Human tongue dorsum microbial communities from NIH, USA - visit 2, subject 158256496 reassembly | 3300008091 | JGI/IMG-ER |
| Human tongue dorsum microbial communities from NIH, USA - visit 1, subject 763820215 reassembly | 3300008099 | JGI/IMG-ER |
| Human tongue dorsum microbial communities from NIH, USA - visit 2, subject 159713063 reassembly | 3300008128 | JGI/IMG-ER |
| Human tongue dorsum microbial communities from NIH, USA - visit 2, subject 763840445 reassembly | 3300008133 | JGI/IMG-ER |
| Human tongue dorsum microbial communities from NIH, USA - visit 2, subject 764811490 reassembly | 3300008136 | JGI/IMG-ER |
| Human tongue dorsum microbial communities from NIH, USA - visit 1, subject 160704339 reassembly | 3300008145 | JGI/IMG-ER |
| Human tongue dorsum microbial communities from NIH, USA - visit number 3 of subject 158883629 reassembly | 3300008273 | JGI/IMG-ER |
| Human tongue dorsum microbial communities from NIH, USA - visit 1, subject 764042746 reassembly | 3300008274 | JGI/IMG-ER |
| Human tongue dorsum microbial communities from NIH, USA - visit 1, subject 764649650 reassembly | 3300008278 | JGI/IMG-ER |
| Human tongue dorsum microbial communities from NIH, USA - visit 1, subject 338793263 reassembly | 3300008279 | JGI/IMG-ER |
| Human tongue dorsum microbial communities from NIH, USA - visit 1, subject 160582958 reassembly | 3300008280 | JGI/IMG-ER |
| Human tongue dorsum microbial communities from NIH, USA - visit 2, subject 638754422 reassembly | 3300008283 | JGI/IMG-ER |
| Human tongue dorsum microbial communities from NIH, USA - visit 1, subject 159753524 replicate 1 reassembly | 3300008305 | JGI/IMG-ER |
| Human tongue dorsum microbial communities from NIH, USA - visit 1, subject 508703490 reassembly | 3300008333 | JGI/IMG-ER |
| Human tongue dorsum microbial communities from NIH, USA - visit 2, subject 370425937 reassembly | 3300008436 | JGI/IMG-ER |
| Human tongue dorsum microbial communities from NIH, USA - visit 1, subject 638754422 reassembly | 3300008490 | JGI/IMG-ER |
| Human tongue dorsum microbial communities from NIH, USA - visit 2, subject 763901136 replicate 2 reassembly | 3300008493 | JGI/IMG-ER |
| Human tongue dorsum microbial communities from NIH, USA - visit number 3 of subject 159753524 reassembly | 3300008504 | JGI/IMG-ER |
| Human tongue dorsum microbial communities from NIH, USA - visit 1, subject 370425937 reassembly | 3300008521 | JGI/IMG-ER |
| Human tongue dorsum microbial communities from NIH, USA - visit 1, subject 160319967 reassembly | 3300008524 | JGI/IMG-ER |
| Human tongue dorsum microbial communities from NIH, USA - visit 1, subject 765337473 reassembly | 3300008537 | JGI/IMG-ER |
| Human tongue dorsum microbial communities from NIH, USA - visit 2, subject 158944319 reassembly | 3300008581 | JGI/IMG-ER |
| Human tongue dorsum microbial communities from NIH, USA - visit 1, subject 765640925 reassembly | 3300008589 | JGI/IMG-ER |
| Human tongue dorsum microbial communities from NIH, USA - visit 1, subject 158337416 reassembly | 3300008664 | JGI/IMG-ER |
| Human tongue dorsum microbial communities from NIH, USA - visit 1, subject 159753524 replicate 2 reassembly | 3300008709 | JGI/IMG-ER |
| Human tongue dorsum microbial communities from NIH, USA - visit 1, subject 763577454 reassembly | 3300008715 | JGI/IMG-ER |
| Human tongue dorsum microbial communities from NIH, USA - visit 1, subject 765013792 reassembly | 3300008734 | JGI/IMG-ER |
| Human tongue dorsum microbial communities from NIH, USA - visit 1, subject 737052003 reassembly | 3300008739 | JGI/IMG-ER |
| Human tongue dorsum microbial communities from NIH, USA - visit 2, subject 159490532 | 3300006245 | JGI/IMG-ER |
| Human tongue dorsum microbial communities from NIH, USA - visit 2, subject 159510762 | 3300006250 | JGI/IMG-ER |
| Human tongue dorsum microbial communities from NIH, USA - visit 2, subject 159369152 | 3300006251 | JGI/IMG-ER |
| Human tongue dorsum microbial communities from NIH, USA - visit 2, subject 764325968 | 3300006256 | JGI/IMG-ER |
| Human tongue dorsum microbial communities from NIH, USA - visit 1, subject 160380657 | 3300006321 | JGI/IMG-ER |
| Human tongue dorsum microbial communities from NIH, USA - visit 1, subject 763860675 | 3300006328 | JGI/IMG-ER |
| Human tongue dorsum microbial communities from NIH, USA - visit 2 of subject 764487809 | 3300006458 | JGI/IMG-ER |
| Human tongue dorsum microbial communities from NIH, USA - visit 1, subject 159369152 | 3300006459 | JGI/IMG-ER |
| Human tongue dorsum microbial communities from NIH, USA - visit 2 of subject 159207311 | 3300006462 | JGI/IMG-ER |
| Human tongue dorsum microbial communities from NIH, USA - visit 2, subject 158479027 | 3300006524 | JGI/IMG-ER |
| Human tongue dorsum microbial communities from NIH, USA - visit 1, subject 404239096 | 3300006564 | JGI/IMG-ER |
| Human tongue dorsum microbial communities from NIH, USA - visit 1, subject 159268001 | 3300006566 | JGI/IMG-ER |
| Human tongue dorsum microbial communities from NIH, USA - visit 1, subject 160158126 | 3300006745 | JGI/IMG-ER |
| Human tongue dorsum microbial communities from NIH, USA - visit 1, subject 159571453 | 3300006832 | JGI/IMG-ER |
| Human tongue dorsum microbial communities from NIH, USA - visit 1, subject 604812005 | 3300007096 | JGI/IMG-ER |
| Human tongue dorsum microbial communities from NIH, USA - visit 1, subject 764325968 | 3300007123 | JGI/IMG-ER |
| Human tongue dorsum microbial communities from NIH, USA - visit 2, subject 159268001 | 3300007124 | JGI/IMG-ER |
| Human tongue dorsum microbial communities from NIH, USA - visit 1, subject 159207311 | 3300007128 | JGI/IMG-ER |
| Human tongue dorsum microbial communities from NIH, USA - visit 1, subject 764305738 | 3300007220 | JGI/IMG-ER |
| Human tongue dorsum microbial communities from NIH, USA - visit 1, subject 159814214 reassembly | 3300007295 | JGI/IMG-ER |
| Human tongue dorsum microbial communities from NIH, USA - visit 1, subject 160603188 reassembly | 3300007300 | JGI/IMG-ER |
| Human tongue dorsum microbial communities from NIH, USA - visit 1, subject 861967750 reassembly | 3300007314 | JGI/IMG-ER |
| Human tongue dorsum microbial communities from NIH, USA - visit 2, subject 159571453 reassembly | 3300007316 | JGI/IMG-ER |
| Human tongue dorsum microbial communities from NIH, USA - visit 1, subject 159551223 reassembly | 3300007358 | JGI/IMG-ER |
| Human tongue dorsum microbial communities from NIH, USA - visit 1, subject 764285508 reassembly | 3300007502 | JGI/IMG-ER |
| Human tongue dorsum microbial communities from NIH, USA - visit 1, subject 764224817 reassembly | 3300007531 | JGI/IMG-ER |
| Human tongue dorsum microbial communities from NIH, USA - visit 1, subject 763961826 reassembly | 3300007724 | JGI/IMG-ER |
| Human tongue dorsum microbial communities from NIH, USA - visit 2, subject 686765762 reassembly | 3300007728 | JGI/IMG-ER |
| Human tongue dorsum microbial communities from NIH, USA - visit 1, subject 159591683 reassembly | 3300007753 | JGI/IMG-ER |
| Human tongue dorsum microbial communities from NIH, USA - visit 1, subject 763435843 reassembly | 3300007803 | JGI/IMG-ER |
| Human tongue dorsum microbial communities from NIH, USA - visit 2, subject 763536994 reassembly | 3300007966 | JGI/IMG-ER |
| Human tongue dorsum microbial communities from NIH, USA - visit 1, subject 764447348 reassembly | 3300007976 | JGI/IMG-ER |
| Human tongue dorsum microbial communities from NIH, USA - visit 1, subject 765094712 reassembly | 3300007979 | JGI/IMG-ER |
| Human tongue dorsum microbial communities from NIH, USA - visit 2, subject 160158126 reassembly | 3300007994 | JGI/IMG-ER |
| Human tongue dorsum microbial communities from NIH, USA - visit 2, subject 764224817 reassembly | 3300008098 | JGI/IMG-ER |
| Human tongue dorsum microbial communities from NIH, USA - visit number 3 of subject 763536994 reassembly | 3300008125 | JGI/IMG-ER |
| Human tongue dorsum microbial communities from NIH, USA - visit 2, subject 764892411 reassembly | 3300008138 | JGI/IMG-ER |
| Human tongue dorsum microbial communities from NIH, USA - visit 2, subject 763982056 reassembly | 3300008141 | JGI/IMG-ER |
| Human tongue dorsum microbial communities from NIH, USA - visit 2, subject 158499257 reassembly | 3300008143 | JGI/IMG-ER |
| Human tongue dorsum microbial communities from NIH, USA - visit 2, subject 763961826 replicate 1 reassembly | 3300008147 | JGI/IMG-ER |
| Human tongue dorsum microbial communities from NIH, USA - visit 1, subject 158499257 reassembly | 3300008153 | JGI/IMG-ER |
| Human tongue dorsum microbial communities from NIH, USA - visit 2, subject 763496533 reassembly | 3300008269 | JGI/IMG-ER |
| Human tongue dorsum microbial communities from NIH, USA - visit 2, subject 764447348 reassembly | 3300008275 | JGI/IMG-ER |
| Human tongue dorsum microbial communities from NIH, USA - visit 2, subject 763860675 reassembly | 3300008276 | JGI/IMG-ER |
| Human tongue dorsum microbial communities from NIH, USA - visit 2, subject 764305738 reassembly | 3300008306 | JGI/IMG-ER |
| Human tongue dorsum microbial communities from NIH, USA - visit 2, subject 159611913 reassembly | 3300008328 | JGI/IMG-ER |
| Human tongue dorsum microbial communities from NIH, USA - visit 2, subject 763961826 replicate 2 reassembly | 3300008345 | JGI/IMG-ER |
| Human tongue dorsum microbial communities from NIH, USA - visit 1, subject 160400887 reassembly | 3300008362 | JGI/IMG-ER |
| Human tongue dorsum microbial communities from NIH, USA - visit 1, subject 765701615 reassembly | 3300008406 | JGI/IMG-ER |
| Human tongue dorsum microbial communities from NIH, USA - visit 1, subject 160218816 reassembly | 3300008420 | JGI/IMG-ER |
| Human tongue dorsum microbial communities from NIH, USA - visit 2, subject 159551223 reassembly | 3300008472 | JGI/IMG-ER |
| Human tongue dorsum microbial communities from NIH, USA - visit 1, subject 160421117 reassembly | 3300008502 | JGI/IMG-ER |
| Human tongue dorsum microbial communities from NIH, USA - visit 1, subject 159632143 reassembly | 3300008503 | JGI/IMG-ER |
| Human tongue dorsum microbial communities from NIH, USA - visit number 3 of subject 159510762 reassembly | 3300008506 | JGI/IMG-ER |
| Human tongue dorsum microbial communities from NIH, USA - visit 1, subject 160765029 reassembly | 3300008514 | JGI/IMG-ER |
| Human tongue dorsum microbial communities from NIH, USA - visit 1, subject 765074482 reassembly | 3300008515 | JGI/IMG-ER |
| Human tongue dorsum microbial communities from NIH, USA - visit 1, subject 764508039 reassembly | 3300008518 | JGI/IMG-ER |
| Human tongue dorsum microbial communities from NIH, USA - visit 1, subject 765135172 reassembly | 3300008522 | JGI/IMG-ER |
| Human tongue dorsum microbial communities from NIH, USA - visit 2, subject 158802708 reassembly | 3300008534 | JGI/IMG-ER |
| Human tongue dorsum microbial communities from NIH, USA - visit 2, subject 159591683 reassembly | 3300008556 | JGI/IMG-ER |
| Human tongue dorsum microbial communities from NIH, USA - visit 2, subject 764083206 reassembly | 3300008575 | JGI/IMG-ER |
| Human tongue dorsum microbial communities from NIH, USA - visit 1, subject 686765762 reassembly | 3300008607 | JGI/IMG-ER |
| Human tongue dorsum microbial communities from NIH, USA - visit 1, subject 823052294 reassembly | 3300008611 | JGI/IMG-ER |
| Human tongue dorsum microbial communities from NIH, USA - visit 1, subject 763536994 reassembly | 3300008621 | JGI/IMG-ER |
| Human tongue dorsum microbial communities from NIH, USA - visit 1, subject 764487809 reassembly | 3300008626 | JGI/IMG-ER |
| Human tongue dorsum microbial communities from NIH, USA - visit 2, subject 765094712 reassembly | 3300008636 | JGI/IMG-ER |
| Human tongue dorsum microbial communities from NIH, USA - visit 2, subject 763435843 reassembly | 3300008638 | JGI/IMG-ER |
| Human tongue dorsum microbial communities from NIH, USA - visit 1, subject 764083206 reassembly | 3300008639 | JGI/IMG-ER |
| Human tongue dorsum microbial communities from NIH, USA - visit 2, subject 604812005 reassembly | 3300008660 | JGI/IMG-ER |
| Human tongue dorsum microbial communities from NIH, USA - visit 1, subject 765560005 reassembly | 3300008730 | JGI/IMG-ER |
| Human tongue dorsum microbial communities from NIH, USA - visit 1, subject 159611913 reassembly | 3300008732 | JGI/IMG-ER |
| Human tongue dorsum microbial communities from NIH, USA - visit 2, subject 159814214 reassembly | 3300008742 | JGI/IMG-ER |
| Human tongue dorsum microbial communities from NIH, USA - visit 1, subject 763982056 reassembly | 3300008747 | JGI/IMG-ER |
| Human tongue dorsum microbial communities from NIH, USA - visit 1, subject 160178356 reassembly | 3300008748 | JGI/IMG-ER |
| Human stool microbial communities from NIH, USA - visit 1, subject 158883629 | 3300006252 | JGI/IMG-ER |
| Human stool microbial communities from NIH, USA - visit 2, subject 159247771 | 3300006258 | JGI/IMG-ER |
| Human stool microbial communities from NIH, USA - visit 1, subject 737052003 | 3300006298 | JGI/IMG-ER |
| Human stool microbial communities from NIH, USA - visit 1, subject 550534656 | 3300006299 | JGI/IMG-ER |
| Human stool microbial communities from NIH, USA - visit 2 of subject 159753524 | 3300006312 | JGI/IMG-ER |
| Human stool microbial communities from NIH, USA - visit 2 of subject 763759525 | 3300006476 | JGI/IMG-ER |
| Human stool microbial communities from NIH, USA - visit 1, subject 764588959 | 3300006502 | JGI/IMG-ER |
| Human stool microbial communities from NIH, USA - visit 2, subject 764062976 | 3300006525 | JGI/IMG-ER |
| Human stool microbial communities from NIH, USA - visit 1, subject 638754422 | 3300007043 | JGI/IMG-ER |
| Human stool microbial communities from NIH, USA - visit 2, subject 246515023 | 3300007055 | JGI/IMG-ER |
| Human stool microbial communities from NIH, USA - visit 1, subject 765620695 | 3300007109 | JGI/IMG-ER |
| Human stool microbial communities from NIH, USA - visit 1, subject 158944319 | 3300007111 | JGI/IMG-ER |
| Human stool microbial communities from NIH, USA - visit 2, subject 764811490 | 3300007138 | JGI/IMG-ER |
| Human stool microbial communities from NIH, USA - visit 1, subject 764062976 | 3300007210 | JGI/IMG-ER |
| Human stool microbial communities from NIH, USA - visit 1, subject 706846339 | 3300007222 | JGI/IMG-ER |
| Human stool microbial communities from NIH, USA - visit 2, subject 764184357 reassembly | 3300007296 | JGI/IMG-ER |
| Human stool microbial communities from NIH, USA - visit 1, subject 159247771 reassembly | 3300007312 | JGI/IMG-ER |
| Human stool microbial communities from NIH, USA - visit 2, subject 158883629 reassembly | 3300007313 | JGI/IMG-ER |
| Human stool microbial communities from NIH, USA - visit 1, subject 765013792 reassembly | 3300007353 | JGI/IMG-ER |
| Human stool microbial communities from NIH, USA - visit 2, subject 158337416 reassembly | 3300007361 | JGI/IMG-ER |
| Human stool microbial communities from NIH, USA - visit 1, subject 675950834 reassembly | 3300007362 | JGI/IMG-ER |
| Human stool microbial communities from NIH, USA - visit 1, subject 763840445 reassembly | 3300007498 | JGI/IMG-ER |
| Human stool microbial communities from NIH, USA - visit 1, subject 809635352 reassembly | 3300007530 | JGI/IMG-ER |
| Human stool microbial communities from NIH, USA - visit number 3 of subject 159227541 reassembly | 3300007641 | JGI/IMG-ER |
| Human stool microbial communities from NIH, USA - visit 2, subject 158256496 reassembly | 3300007669 | JGI/IMG-ER |
| Human stool microbial communities from NIH, USA - visit 1, subject 763577454 reassembly | 3300007742 | JGI/IMG-ER |
| Human stool microbial communities from NIH, USA - visit 1, subject 764143897 reassembly | 3300007796 | JGI/IMG-ER |
| Human stool microbial communities from NIH, USA - visit 1, subject 159753524 replicate 1 reassembly | 3300007801 | JGI/IMG-ER |
| Human stool microbial communities from NIH, USA - visit 1, subject 508703490 reassembly | 3300007805 | JGI/IMG-ER |
| Human stool microbial communities from NIH, USA - visit 1, subject 764042746 reassembly | 3300007806 | JGI/IMG-ER |
| Human stool microbial communities from NIH, USA - visit 1, subject 160704339 reassembly | 3300007921 | JGI/IMG-ER |
| Human stool microbial communities from NIH, USA - visit 1, subject 246515023 reassembly | 3300007975 | JGI/IMG-ER |
| Human stool microbial communities from NIH, USA - visit 1, subject 159915365 reassembly | 3300007990 | JGI/IMG-ER |
| Human stool microbial communities from NIH, USA - visit 1, subject 764649650 reassembly | 3300007991 | JGI/IMG-ER |
| Human stool microbial communities from NIH, USA - visit 1, subject 159227541 reassembly | 3300008060 | JGI/IMG-ER |
| Human stool microbial communities from NIH, USA - visit 1, subject 160582958 reassembly | 3300008079 | JGI/IMG-ER |
| Human stool microbial communities from NIH, USA - visit 2, subject 158944319 reassembly | 3300008100 | JGI/IMG-ER |
| Human stool microbial communities from NIH, USA - visit 2, subject 159227541 reassembly | 3300008101 | JGI/IMG-ER |
| Human stool microbial communities from NIH, USA - visit 1, subject 158337416 reassembly | 3300008272 | JGI/IMG-ER |
| Human stool microbial communities from NIH, USA - visit 1, subject 763820215 reassembly | 3300008301 | JGI/IMG-ER |
| Human stool microbial communities from NIH, USA - visit 1, subject 158458797 reassembly | 3300008326 | JGI/IMG-ER |
| Human stool microbial communities from NIH, USA - visit 2, subject 370425937 reassembly | 3300008350 | JGI/IMG-ER |
| Human stool microbial communities from NIH, USA - visit 2, subject 764042746 reassembly | 3300008383 | JGI/IMG-ER |
| Human stool microbial communities from NIH, USA - visit 1, subject 370425937 reassembly | 3300008461 | JGI/IMG-ER |
| Human stool microbial communities from NIH, USA - visit 2, subject 763577454 reassembly | 3300008479 | JGI/IMG-ER |
| Human stool microbial communities from NIH, USA - visit 1, subject 160643649 reassembly | 3300008512 | JGI/IMG-ER |
| Human stool microbial communities from NIH, USA - visit 1, subject 160319967 reassembly | 3300008513 | JGI/IMG-ER |
| Human stool microbial communities from NIH, USA - visit 1, subject 159733294 reassembly | 3300008547 | JGI/IMG-ER |
| Human stool microbial communities from NIH, USA - visit 2, subject 159713063 reassembly | 3300008561 | JGI/IMG-ER |
| Human stool microbial communities from NIH, USA - visit 1, subject 763901136 reassembly | 3300008571 | JGI/IMG-ER |
| Human stool microbial communities from NIH, USA - visit 1, subject 765034022 reassembly | 3300008585 | JGI/IMG-ER |
| Human stool microbial communities from NIH, USA - visit 1, subject 158742018 reassembly | 3300008599 | JGI/IMG-ER |
| Human stool microbial communities from NIH, USA - visit 1, subject 765640925 reassembly | 3300008622 | JGI/IMG-ER |
| Human stool microbial communities from NIH, USA - visit 2, subject 763901136 reassembly | 3300008640 | JGI/IMG-ER |
| Human stool microbial communities from NIH, USA - visit number 3 of subject 159753524 reassembly | 3300008676 | JGI/IMG-ER |
| Human stool microbial communities from NIH, USA - visit 2, subject 809635352 SPADES reassembly | 3300008692 | JGI/IMG-ER |
| Human stool microbial communities from NIH, USA - visit 1, subject 160502038 reassembly | 3300008722 | JGI/IMG-ER |
| Human stool microbial communities from NIH, USA - visit 2, subject 763840445 reassembly | 3300008728 | JGI/IMG-ER |
| Human stool microbial communities from NIH, USA - visit 1, subject 763759525 reassembly | 3300008733 | JGI/IMG-ER |
| Human stool microbial communities from NIH, USA - visit 1, subject 338793263 reassembly | 3300008737 | JGI/IMG-ER |
| Human stool microbial communities from NIH, USA - visit 2, subject 159005010 reassembly | 3300008750 | JGI/IMG-ER |
| Human stool microbial communities from NIH, USA - visit 1, subject 159753524 replicate 2 reassembly | 3300009343 | JGI/IMG-ER |
| Human stool microbial communities from NIH, USA - visit 2, subject 764224817 | 3300006257 | JGI/IMG-ER |
| Human stool microbial communities from NIH, USA - visit 1, subject 160380657 | 3300006296 | JGI/IMG-ER |
| Human stool microbial communities from NIH, USA - visit 1, subject 159146620 | 3300006349 | JGI/IMG-ER |
| Human stool microbial communities from NIH, USA - visit 2 of subject 159207311 | 3300006463 | JGI/IMG-ER |
| Human stool microbial communities from NIH, USA - visit 1, subject 160400887 | 3300006464 | JGI/IMG-ER |
| Human stool microbial communities from NIH, USA - visit 1, subject 159591683 | 3300006487 | JGI/IMG-ER |
| Human stool microbial communities from NIH, USA - visit 1, subject 159268001 | 3300006499 | JGI/IMG-ER |
| Human stool microbial communities from NIH, USA - visit 1, subject 764508039 | 3300006523 | JGI/IMG-ER |
| Human stool microbial communities from NIH, USA - visit 2 of subject 765074482 replicate 2 | 3300006568 | JGI/IMG-ER |
| Human stool microbial communities from NIH, USA - visit 2, subject 686765762 | 3300006722 | JGI/IMG-ER |
| Human stool microbial communities from NIH, USA - visit 1, subject 159632143 | 3300006739 | JGI/IMG-ER |
| Human stool microbial communities from NIH, USA - visit 1, subject 823052294 | 3300006746 | JGI/IMG-ER |
| Human stool microbial communities from NIH, USA - visit 1, subject 160218816 | 3300006747 | JGI/IMG-ER |
| Human stool microbial communities from NIH, USA - visit 2, subject 159591683 | 3300007062 | JGI/IMG-ER |
| Human stool microbial communities from NIH, USA - visit 2, subject 158924089 | 3300007097 | JGI/IMG-ER |
| Human stool microbial communities from NIH, USA - visit 1, subject 765135172 | 3300007098 | JGI/IMG-ER |
| Human stool microbial communities from NIH, USA - visit 2, subject 764447348 | 3300007105 | JGI/IMG-ER |
| Human stool microbial communities from NIH, USA - visit 1, subject 160421117 | 3300007108 | JGI/IMG-ER |
| Human stool microbial communities from NIH, USA - visit 2, subject 763536994 | 3300007184 | JGI/IMG-ER |
| Human stool microbial communities from NIH, USA - visit 1, subject 159510762 reassembly | 3300007288 | JGI/IMG-ER |
| Human stool microbial communities from NIH, USA - visit 1, subject 765094712 reassembly | 3300007292 | JGI/IMG-ER |
| Human stool microbial communities from NIH, USA - visit 1, subject 160603188 reassembly | 3300007298 | JGI/IMG-ER |
| Human stool microbial communities from NIH, USA - visit 1, subject 160765029 reassembly | 3300007299 | JGI/IMG-ER |
| Human stool microbial communities from NIH, USA - visit 2, subject 158802708 reassembly | 3300007305 | JGI/IMG-ER |
| Human stool microbial communities from NIH, USA - visit 1, subject 159611913 reassembly | 3300007356 | JGI/IMG-ER |
| Human stool microbial communities from the National Institute of Health, USA - subject 158499257, visit 2 reassembly | 3300007360 | JGI/IMG-ER |
| Human stool microbial communities from NIH, USA - visit 1, subject 764285508 reassembly | 3300007501 | JGI/IMG-ER |
| Human stool microbial communities from NIH, USA - visit 1, subject 763496533 reassembly | 3300007524 | JGI/IMG-ER |
| Human stool microbial communities from NIH, USA - visit 1, subject 604812005 reassembly | 3300007650 | JGI/IMG-ER |
| Human stool microbial communities from NIH, USA - visit 2, subject 158479027 reassembly | 3300007657 | JGI/IMG-ER |
| Human stool microbial communities from NIH, USA - visit 1, subject 765074482 reassembly | 3300007664 | JGI/IMG-ER |
| Human stool microbial communities from NIH, USA - visit 1, subject 160158126 reassembly | 3300007705 | JGI/IMG-ER |
| Human stool microbial communities from NIH, USA - visit 2, subject 763678604 reassembly | 3300007714 | JGI/IMG-ER |
| Human stool microbial communities from NIH, USA - visit 2, subject 159551223 reassembly | 3300007717 | JGI/IMG-ER |
| Human stool microbial communities from NIH, USA - visit 2, subject 763860675 reassembly | 3300007793 | JGI/IMG-ER |
| Human stool microbial communities from NIH, USA - visit 2, subject 764325968 reassembly | 3300007797 | JGI/IMG-ER |
| Human stool microbial communities from NIH, USA - visit 2, subject 160158126 reassembly | 3300007798 | JGI/IMG-ER |
| Human stool microbial communities from NIH, USA - visit 2, subject 159268001 reassembly | 3300007802 | JGI/IMG-ER |
| Human stool microbial communities from NIH, USA - visit 2, subject 159814214 reassembly | 3300007804 | JGI/IMG-ER |
| Human stool microbial communities from NIH, USA - visit 1, subject 765560005 reassembly | 3300007853 | JGI/IMG-ER |
| Human stool microbial communities from NIH, USA - visit 2, subject 159369152 reassembly | 3300007969 | JGI/IMG-ER |
| Human stool microbial communities from NIH, USA - visit 2, subject 159510762 reassembly | 3300008058 | JGI/IMG-ER |
| Human stool microbial communities from NIH, USA - visit 1, subject 764447348 reassembly | 3300008063 | JGI/IMG-ER |
| Human stool microbial communities from NIH, USA - visit 1, subject 763860675 reassembly | 3300008077 | JGI/IMG-ER |
| Human stool microbial communities from NIH, USA - visit 2, subject 765074482 replicate 1 reassembly | 3300008081 | JGI/IMG-ER |
| Human stool microbial communities from NIH, USA - visit 1, subject 765701615 reassembly | 3300008096 | JGI/IMG-ER |
| Human stool microbial communities from NIH, USA - visit 2, subject 764892411 reassembly | 3300008103 | JGI/IMG-ER |
| Human stool microbial communities from NIH, USA - visit 2, subject 765094712 reassembly | 3300008129 | JGI/IMG-ER |
| Human stool microbial communities from NIH, USA - visit 1, subject 159207311 reassembly | 3300008132 | JGI/IMG-ER |
| Human stool microbial communities from NIH, USA - visit 2, subject 604812005 SPADES reassembly | 3300008146 | JGI/IMG-ER |
| Human stool microbial communities from NIH, USA - visit 2, subject 764669880 reassembly | 3300008149 | JGI/IMG-ER |
| Human stool microbial communities from NIH, USA - visit 1, subject 159551223 reassembly | 3300008260 | JGI/IMG-ER |
| Human stool microbial communities from NIH, USA - visit 1, subject 158499257 reassembly | 3300008268 | JGI/IMG-ER |
| Human stool microbial communities from NIH, USA - visit 1, subject 159166850 reassembly | 3300008282 | JGI/IMG-ER |
| Human stool microbial communities from NIH, USA - visit 2, subject 159571453 reassembly | 3300008299 | JGI/IMG-ER |
| Human stool microbial communities from NIH, USA - visit 1, subject 764224817 reassembly | 3300008360 | JGI/IMG-ER |
| Human stool microbial communities from NIH, USA - visit 1, subject 764325968 reassembly | 3300008361 | JGI/IMG-ER |
| Human stool microbial communities from NIH, USA - visit number 3 of subject 763536994 reassembly | 3300008385 | JGI/IMG-ER |
| Human stool microbial communities from NIH, USA - visit 2, subject 764002286 reassembly | 3300008404 | JGI/IMG-ER |
| Human stool microbial communities from NIH, USA - visit 1, subject 763961826 reassembly | 3300008421 | JGI/IMG-ER |
| Human stool microbial communities from NIH, USA - visit 1, subject 686765762 reassembly | 3300008455 | JGI/IMG-ER |
| Human stool microbial communities from NIH, USA - visit 2, subject 763982056 reassembly | 3300008478 | JGI/IMG-ER |
| Human stool microbial communities from NIH, USA - visit 2, subject 159490532 reassembly | 3300008482 | JGI/IMG-ER |
| Human stool microbial communities from NIH, USA - visit 1, subject 159814214 reassembly | 3300008491 | JGI/IMG-ER |
| Human stool microbial communities from NIH, USA - visit 1, subject 160178356 reassembly | 3300008496 | JGI/IMG-ER |
| Human stool microbial communities from NIH, USA - visit 1, subject 404239096 reassembly | 3300008497 | JGI/IMG-ER |
| Human stool microbial communities from NIH, USA - visit 1, subject 159490532 reassembly | 3300008499 | JGI/IMG-ER |
| Human stool microbial communities from NIH, USA - visit 1, subject 764487809 reassembly | 3300008520 | JGI/IMG-ER |
| Human stool microbial communities from NIH, USA - visit 2, subject 763597684 reassembly | 3300008523 | JGI/IMG-ER |
| Human stool microbial communities from NIH, USA - visit 1, subject 158802708 reassembly | 3300008551 | JGI/IMG-ER |
| Human stool microbial communities from NIH, USA - visit 1, subject 159369152 reassembly | 3300008619 | JGI/IMG-ER |
| Human stool microbial communities from NIH, USA - visit 1, subject 763536994 reassembly | 3300008620 | JGI/IMG-ER |
| Human stool microbial communities from NIH, USA - visit 2, subject 764487809 reassembly | 3300008672 | JGI/IMG-ER |
| Human stool microbial communities from NIH, USA - visit 1, subject 763982056 reassembly | 3300008705 | JGI/IMG-ER |
| Human stool microbial communities from NIH, USA - visit 1, subject 159571453 reassembly | 3300008712 | JGI/IMG-ER |
| Human stool microbial communities from NIH, USA - visit 1, subject 861967750 reassembly | 3300008716 | JGI/IMG-ER |
| Human stool microbial communities from NIH, USA - visit 1, subject 763678604 reassembly | 3300008725 | JGI/IMG-ER |
| Human stool microbial communities from NIH, USA - visit 1, subject 764669880 reassembly | 3300008726 | JGI/IMG-ER |
| Human stool microbial communities from NIH, USA - visit 2, subject 763496533 reassembly | 3300008744 | JGI/IMG-ER |
| Human fecal microbial communities from the University of Arizona (HMP) - Ef1 | 2149837015 | JGI/IMG-ER |
| Human fecal microbial communities from the University of Arizona (HMP) - Ef2 | 2149837016 | JGI/IMG-ER |
| Human fecal microbial communities from the University of Arizona (HMP) - Ef3 | 2149837017 | JGI/IMG-ER |
| Human fecal microbial communities from the University of Arizona (HMP) - Em1 | 2149837018 | JGI/IMG-ER |
| Human fecal microbial communities from the University of Arizona (HMP) - Em2 | 2149837019 | JGI/IMG-ER |
| Human fecal microbial communities from the University of Arizona (HMP) - Em3 | 2149837020 | JGI/IMG-ER |
| Human fecal microbial communities from the University of Arizona (HMP) - UAf1 | 2149837021 | JGI/IMG-ER |
| Human fecal microbial communities from the University of Arizona (HMP) - UAf2 | 2149837022 | JGI/IMG-ER |
| Human fecal microbial communities from the University of Arizona (HMP) - UAm1 | 2149837023 | JGI/IMG-ER |
| Human fecal microbial communities from the University of Arizona (HMP) - UAm2 | 2149837024 | JGI/IMG-ER |
| Human supragingival plaque microbial communities from NIH, USA - visit 2, subject 159005010 | 3300006247 | JGI/IMG-ER |
| Human supragingival plaque microbial communities from NIH, USA - visit 1, subject 246515023 | 3300006248 | JGI/IMG-ER |
| Human supragingival plaque microbial communities from NIH, USA - visit 1, subject 737052003 | 3300006255 | JGI/IMG-ER |
| Human supragingival plaque microbial communities from NIH, USA - visit 1, subject 764588959 | 3300006261 | JGI/IMG-ER |
| Human supragingival plaque microbial communities from NIH, USA - visit 1, subject 550534656 | 3300006290 | JGI/IMG-ER |
| Human supragingival plaque microbial communities from NIH, USA - visit 1, subject 809635352 | 3300006292 | JGI/IMG-ER |
| Human supragingival plaque microbial communities from NIH, USA - visit 1, subject 370425937 | 3300006317 | JGI/IMG-ER |
| Human supragingival plaque microbial communities from NIH, USA - visit 1, subject 159227541 | 3300006333 | JGI/IMG-ER |
| Human supragingival plaque microbial communities from NIH, USA - visit 1, subject 765337473 | 3300006457 | JGI/IMG-ER |
| Human supragingival plaque microbial communities from NIH, USA - visit 1, subject 158742018 | 3300006461 | JGI/IMG-ER |
| Human supragingival plaque microbial communities from NIH, USA - visit number 1 of subject 675950834 | 3300006488 | JGI/IMG-ER |
| Human supragingival plaque microbial communities from NIH, USA - visit 2, subject 809635352 | 3300006522 | JGI/IMG-ER |
| Human supragingival plaque microbial communities from NIH, USA - visit 2, subject 158256496 | 3300006744 | JGI/IMG-ER |
| Human supragingival plaque microbial communities from NIH, USA - visit 1, subject 764042746 | 3300007091 | JGI/IMG-ER |
| Human supragingival plaque microbial communities from NIH, USA - visit 2, subject 763820215 | 3300007093 | JGI/IMG-ER |
| Human supragingival plaque microbial communities from NIH, USA - visit 1, subject 765034022 | 3300007104 | JGI/IMG-ER |
| Human supragingival plaque microbial communities from NIH, USA - visit 2, subject 158944319 | 3300007121 | JGI/IMG-ER |
| Human supragingival plaque microbial communities from NIH, USA - visit 1, subject 158337416 | 3300007126 | JGI/IMG-ER |
| Human supragingival plaque microbial communities from NIH, USA - visit 1, subject 158458797 | 3300007135 | JGI/IMG-ER |
| Human supragingival plaque microbial communities from NIH, USA - visit 1, subject 763759525 reassembly | 3300007317 | JGI/IMG-ER |
| Human supragingival plaque microbial communities from NIH, USA - visit 2, subject 763759525 reassembly | 3300007335 | JGI/IMG-ER |
| Human supragingival plaque microbial communities from NIH, USA - visit 1, subject 764649650 reassembly | 3300007347 | JGI/IMG-ER |
| Human supragingival plaque microbial communities from NIH, USA - visit 1, subject 765640925 reassembly | 3300007355 | JGI/IMG-ER |
| Human supragingival plaque microbial communities from NIH, USA - visit 1, subject 160319967 reassembly | 3300007357 | JGI/IMG-ER |
| Human supragingival plaque microbial communities from NIH, USA - visit 1, subject 763840445 reassembly | 3300007500 | JGI/IMG-ER |
| Human supragingival plaque microbial communities from NIH, USA - visit 1, subject 159915365 reassembly | 3300007638 | JGI/IMG-ER |
| Human supragingival plaque microbial communities from NIH, USA - visit 2, subject 763840445 reassembly | 3300007646 | JGI/IMG-ER |
| Human supragingival plaque microbial communities from NIH, USA - visit number 3 of subject 158883629 reassembly | 3300007732 | JGI/IMG-ER |
| Human supragingival plaque microbial communities from NIH, USA - visit 1, subject 160502038 reassembly | 3300007746 | JGI/IMG-ER |
| Human supragingival plaque microbial communities from NIH, USA - visit 1, subject 160704339 reassembly | 3300007993 | JGI/IMG-ER |
| Human supragingival plaque microbial communities from NIH, USA - visit 2, subject 158883629 reassembly | 3300008073 | JGI/IMG-ER |
| Human supragingival plaque microbial communities from NIH, USA - visit 1, subject 765620695 reassembly | 3300008075 | JGI/IMG-ER |
| Human supragingival plaque microbial communities from NIH, USA - visit 2, subject 158337416 reassembly | 3300008082 | JGI/IMG-ER |
| Human supragingival plaque microbial communities from NIH, USA - visit 2, subject 159713063 reassembly | 3300008131 | JGI/IMG-ER |
| Human supragingival plaque microbial communities from NIH, USA - visit 2, subject 764143897 reassembly | 3300008144 | JGI/IMG-ER |
| Human supragingival plaque microbial communities from NIH, USA - visit 2, subject 246515023 reassembly | 3300008150 | JGI/IMG-ER |
| Human supragingival plaque microbial communities from NIH, USA - visit 1, subject 160582958 reassembly | 3300008152 | JGI/IMG-ER |
| Human supragingival plaque microbial communities from NIH, USA - visit 2, subject 370425937 reassembly | 3300008155 | JGI/IMG-ER |
| Human supragingival plaque microbial communities from NIH, USA - visit 1, subject 706846339 reassembly | 3300008161 | JGI/IMG-ER |
| Human supragingival plaque microbial communities from NIH, USA - visit 2, subject 638754422 reassembly | 3300008271 | JGI/IMG-ER |
| Human supragingival plaque microbial communities from NIH, USA - visit 1, subject 508703490 reassembly | 3300008335 | JGI/IMG-ER |
| Human supragingival plaque microbial communities from NIH, USA - visit 2, subject 159247771 reassembly | 3300008341 | JGI/IMG-ER |
| Human supragingival plaque microbial communities from NIH, USA - visit 2, subject 763577454 reassembly | 3300008364 | JGI/IMG-ER |
| Human supragingival plaque microbial communities from NIH, USA - visit 2, subject 764042746 reassembly | 3300008403 | JGI/IMG-ER |
| Human supragingival plaque microbial communities from NIH, USA - visit 1, subject 638754422 reassembly | 3300008412 | JGI/IMG-ER |
| Human supragingival plaque microbial communities from NIH, USA - visit 1, subject 160643649 reassembly | 3300008454 | JGI/IMG-ER |
| Human supragingival plaque microbial communities from NIH, USA - visit 2, subject 764811490 reassembly | 3300008489 | JGI/IMG-ER |
| Human supragingival plaque microbial communities from NIH, USA - visit 1, subject 158944319 reassembly | 3300008594 | JGI/IMG-ER |
| Human supragingival plaque microbial communities from NIH, USA - visit 1, subject 763901136 reassembly | 3300008635 | JGI/IMG-ER |
| Human supragingival plaque microbial communities from NIH, USA - visit 1, subject 764143897 reassembly | 3300008650 | JGI/IMG-ER |
| Human supragingival plaque microbial communities from NIH, USA - visit 1, subject 763577454 reassembly | 3300008679 | JGI/IMG-ER |
| Human supragingival plaque microbial communities from NIH, USA - visit 2, subject 763901136 reassembly | 3300008680 | JGI/IMG-ER |
| Human supragingival plaque microbial communities from NIH, USA - visit 2, subject 764062976 reassembly | 3300008682 | JGI/IMG-ER |
| Human supragingival plaque microbial communities from NIH, USA - visit 1, subject 159753524 reassembly | 3300008700 | JGI/IMG-ER |
| Human supragingival plaque microbial communities from NIH, USA - visit 1, subject 338793263 reassembly | 3300008727 | JGI/IMG-ER |
| Human supragingival plaque microbial communities from NIH, USA - visit 1, subject 765013792 reassembly | 3300009363 | JGI/IMG-ER |
| Human supragingival plaque microbial communities from NIH, USA - visit 1, subject 764508039 | 3300006249 | JGI/IMG-ER |
| Human supragingival plaque microbial communities from NIH, USA - visit 2, subject 159510762 | 3300006253 | JGI/IMG-ER |
| Human supragingival plaque microbial communities from NIH, USA - visit 1, subject 765135172 | 3300006294 | JGI/IMG-ER |
| Human supragingival plaque microbial communities from NIH, USA - visit 1, subject 160603188 | 3300006295 | JGI/IMG-ER |
| Human supragingival plaque microbial communities from NIH, USA - visit 1, subject 159551223 | 3300006322 | JGI/IMG-ER |
| Human supragingival plaque microbial communities from NIH, USA - visit 2 of subject 158802708 | 3300006348 | JGI/IMG-ER |
| Human supragingival plaque microbial communities from NIH, USA - visit 1, subject 159369152 | 3300006479 | JGI/IMG-ER |
| Human supragingival plaque microbial communities from NIH, USA - visit 1, subject 159591683 | 3300006498 | JGI/IMG-ER |
| Human supragingival plaque microbial communities from NIH, USA - visit 1, subject 159207311 | 3300006743 | JGI/IMG-ER |
| Human supragingival plaque microbial communities from NIH, USA - visit 1, subject 160765029 | 3300007066 | JGI/IMG-ER |
| Human supragingival plaque microbial communities from NIH, USA - visit 2, subject 764305738 | 3300007100 | JGI/IMG-ER |
| Human supragingival plaque microbial communities from NIH, USA - visit 2, subject 763860675 | 3300007119 | JGI/IMG-ER |
| Human supragingival plaque microbial communities from NIH, USA - visit 2, subject 159369152 | 3300007120 | JGI/IMG-ER |
| Human supragingival plaque microbial communities from NIH, USA - visit 2, subject 763982056 | 3300007122 | JGI/IMG-ER |
| Human supragingival plaque microbial communities from NIH, USA - visit 1, subject 763961826 | 3300007204 | JGI/IMG-ER |
| Human supragingival plaque microbial communities from NIH, USA - visit 1, subject 160380657 reassembly | 3300007294 | JGI/IMG-ER |
| Human supragingival plaque microbial communities from NIH, USA - visit 1, subject 158479027 reassembly | 3300007320 | JGI/IMG-ER |
| Human supragingival plaque microbial communities from NIH, USA - visit 1, subject 763860675 reassembly | 3300007348 | JGI/IMG-ER |
| Human supragingival plaque microbial communities from NIH, USA - visit 2, subject 158499257 reassembly | 3300007368 | JGI/IMG-ER |
| Human supragingival plaque microbial communities from NIH, USA - visit 1, subject 764487809 reassembly | 3300007499 | JGI/IMG-ER |
| Human supragingival plaque microbial communities from NIH, USA - visit 1, subject 765094712 reassembly | 3300007654 | JGI/IMG-ER |
| Human supragingival plaque microbial communities from NIH, USA - visit 1, subject 765074482 reassembly | 3300007666 | JGI/IMG-ER |
| Human supragingival plaque microbial communities from NIH, USA - visit 2, subject 764325968 reassembly | 3300007713 | JGI/IMG-ER |
| Human supragingival plaque microbial communities from NIH, USA - visit 1, subject 604812005 reassembly | 3300007736 | JGI/IMG-ER |
| Human supragingival plaque microbial communities from NIH, USA - visit 1, subject 159611913 reassembly | 3300007774 | JGI/IMG-ER |
| Human supragingival plaque microbial communities from NIH, USA - visit 1, subject 159268001 reassembly | 3300007791 | JGI/IMG-ER |
| Human supragingival plaque microbial communities from NIH, USA - visit 1, subject 159571453 reassembly | 3300007794 | JGI/IMG-ER |
| Human supragingival plaque microbial communities from NIH, USA - visit 2, subject 763435843 reassembly | 3300007925 | JGI/IMG-ER |
| Human supragingival plaque microbial communities from NIH, USA - visit 2, subject 159207311 reassembly | 3300007980 | JGI/IMG-ER |
| Human supragingival plaque microbial communities from NIH, USA - visit 2, subject 764083206 reassembly | 3300007997 | JGI/IMG-ER |
| Human supragingival plaque microbial communities from NIH, USA - visit number 3 of subject 763536994 reassembly | 3300008059 | JGI/IMG-ER |
| Human supragingival plaque microbial communities from NIH, USA - visit 1, subject 159632143 reassembly | 3300008078 | JGI/IMG-ER |
| Human supragingival plaque microbial communities from NIH, USA - visit 1, subject 764447348 reassembly | 3300008083 | JGI/IMG-ER |
| Human supragingival plaque microbial communities from NIH, USA - visit 1, subject 764285508 reassembly | 3300008089 | JGI/IMG-ER |
| Human supragingival plaque microbial communities from NIH, USA - visit 1, subject 404239096 reassembly | 3300008102 | JGI/IMG-ER |
| Human supragingival plaque microbial communities from NIH, USA - visit number 3 of subject 159510762 reassembly | 3300008123 | JGI/IMG-ER |
| Human supragingival plaque microbial communities from NIH, USA - visit 2, subject 763961826 reassembly | 3300008126 | JGI/IMG-ER |
| Human supragingival plaque microbial communities from NIH, USA - visit 2, subject 159571453 reassembly | 3300008134 | JGI/IMG-ER |
| Human supragingival plaque microbial communities from NIH, USA - visit 1, subject 764224817 reassembly | 3300008135 | JGI/IMG-ER |
| Human supragingival plaque microbial communities from NIH, USA - visit 2, subject 763496533 reassembly | 3300008140 | JGI/IMG-ER |
| Human supragingival plaque microbial communities from NIH, USA - visit 1, subject 159814214 reassembly | 3300008142 | JGI/IMG-ER |
| Human supragingival plaque microbial communities from NIH, USA - visit 1, subject 160178356 reassembly | 3300008159 | JGI/IMG-ER |
| Human supragingival plaque microbial communities from NIH, USA - visit 1, subject 686765762 reassembly | 3300008300 | JGI/IMG-ER |
| Human supragingival plaque microbial communities from NIH, USA - visit 1, subject 160421117 reassembly | 3300008304 | JGI/IMG-ER |
| Human supragingival plaque microbial communities from NIH, USA - visit 1, subject 765560005 reassembly | 3300008334 | JGI/IMG-ER |
| Human supragingival plaque microbial communities from NIH, USA - visit 1, subject 159490532 reassembly | 3300008337 | JGI/IMG-ER |
| Human supragingival plaque microbial communities from NIH, USA - visit 2, subject 159268001 reassembly | 3300008346 | JGI/IMG-ER |
| Human supragingival plaque microbial communities from NIH, USA - visit 2, subject 764892411 reassembly | 3300008347 | JGI/IMG-ER |
| Human supragingival plaque microbial communities from NIH, USA - visit 1, subject 764305738 reassembly | 3300008363 | JGI/IMG-ER |
| Human supragingival plaque microbial communities from the National Institute of Health, USA - subject 159490532, visit 2 reassembly | 3300008405 | JGI/IMG-ER |
| Human supragingival plaque microbial communities from NIH, USA - visit 1, subject 763982056 reassembly | 3300008408 | JGI/IMG-ER |
| Human supragingival plaque microbial communities from NIH, USA - visit 2, subject 160158126 reassembly | 3300008480 | JGI/IMG-ER |
| Human supragingival plaque microbial communities from NIH, USA - visit 1, subject 160400887 reassembly | 3300008481 | JGI/IMG-ER |
| Human supragingival plaque microbial communities from NIH, USA - visit 1, subject 764325968 reassembly | 3300008485 | JGI/IMG-ER |
| Human supragingival plaque microbial communities from NIH, USA - visit 1, subject 160158126 reassembly | 3300008486 | JGI/IMG-ER |
| Human supragingival plaque microbial communities from NIH, USA - visit 2, subject 764224817 reassembly | 3300008505 | JGI/IMG-ER |
| Human supragingival plaque microbial communities from NIH, USA - visit 2, subject 604812005 reassembly | 3300008541 | JGI/IMG-ER |
| Human supragingival plaque microbial communities from NIH, USA - visit 1, subject 158499257 reassembly | 3300008565 | JGI/IMG-ER |
| Human supragingival plaque microbial communities from NIH, USA - visit 1, subject 765701615 reassembly | 3300008634 | JGI/IMG-ER |
| Human supragingival plaque microbial communities from NIH, USA - visit 1, subject 763496533 reassembly | 3300008645 | JGI/IMG-ER |
| Human supragingival plaque microbial communities from NIH, USA - visit 1, subject 861967750 reassembly | 3300008688 | JGI/IMG-ER |
| Human supragingival plaque microbial communities from NIH, USA - visit 1, subject 764083206 replicate 1 reassembly | 3300008695 | JGI/IMG-ER |
| Human supragingival plaque microbial communities from NIH, USA - visit 2, subject 158479027 reassembly | 3300008717 | JGI/IMG-ER |
| Human supragingival plaque microbial communities from NIH, USA - visit 2, subject 764487809 reassembly | 3300008729 | JGI/IMG-ER |
| Human supragingival plaque microbial communities from NIH, USA - visit 2, subject 159591683 reassembly | 3300008743 | JGI/IMG-ER |
| Human supragingival plaque microbial communities from NIH, USA - visit 1, subject 764083206 replicate 2 reassembly | 3300008745 | JGI/IMG-ER |
| Human supragingival plaque microbial communities from NIH, USA - visit 2, subject 159611913 reassembly | 3300008746 | JGI/IMG-ER |
| Human supragingival plaque microbial communities from NIH, USA - visit 2, subject 686765762 reassembly | 3300009294 | JGI/IMG-ER |
| Human supragingival plaque microbial communities from NIH, USA - visit 2, subject 159551223 reassembly | 3300009381 | JGI/IMG-ER |
| Human supragingival plaque microbial communities from NIH, USA - visit 2, subject 159814214 reassembly | 3300009393 | JGI/IMG-ER |
| Left Retroauricular Crease | SRS016944 | www.hmpdacc.org |
| Left Retroauricular Crease | SRS024482 | www.hmpdacc.org |
| Left Retroauricular Crease | SRS013258 | www.hmpdacc.org |
| Left Retroauricular Crease | SRS024596 | www.hmpdacc.org |
| Left Retroauricular Crease | SRS024620 | www.hmpdacc.org |
| Left Retroauricular Crease | SRS020261 | www.hmpdacc.org |
| Left Retroauricular Crease | SRS017849 | www.hmpdacc.org |
| Right Retroauricular Crease | SRS058182 | www.hmpdacc.org |
| Right Retroauricular Crease | SRS013261 | www.hmpdacc.org |
| Right Retroauricular Crease | SRS024598 | www.hmpdacc.org |
| Right Retroauricular Crease | SRS020263 | www.hmpdacc.org |
| Right Retroauricular Crease | SRS017851 | www.hmpdacc.org |
| Right Retroauricular Crease | SRS045606 | www.hmpdacc.org |
| Right Retroauricular Crease | SRS058221 | www.hmpdacc.org |
| Right Retroauricular Crease | SRS019116 | www.hmpdacc.org |
| Right Retroauricular Crease | SRS015381 | www.hmpdacc.org |
| Right Retroauricular Crease | SRS046688 | www.hmpdacc.org |
| Right Retroauricular Crease | SRS018978 | www.hmpdacc.org |
| Right Retroauricular Crease | SRS057083 | www.hmpdacc.org |
| Right Retroauricular Crease | SRS052988 | www.hmpdacc.org |

**Supplementary Table 2.** **Pristine environment samples.**

271 different pristine environments were screened to search for ARGs detected in the analyzed HMP samples. Pristine environments were classified in 5 different groups: polar, desert, cave, hot spring, and submarine volcano environments. At the table the genome or sample name could be found along with the IMG Genome ID at JGI-IMG/ER (Markowitz et al., 2012) and the environment classification.

| **Clasified as** | **Genome Name / Sample Name** | **IMG Genome ID** |
| --- | --- | --- |
| Arid desert | Desert soil subsurface microbial communities from Kuchchh Desert, Runn of Kuch, Gujarat, India | 3300006060 |
| Arid desert | Gypsum crust hypoendolithic microbial communities from the Atacama Desert, Chile - KM37, HE | 3300013024 |
| Arid desert | Gypsum rock hypoendolithic microbial communities from the Atacama Desert, Chile - Cordon de Lila | 3300013026 |
| Arid desert | Gypsum rock hypoendolithic microbial communities from the Atacama Desert, Chile - Monturaqui | 3300013027 |
| Arid desert | Soil crust microbial communities from Colorado Plateau, Utah, USA - late stage, 18 hrs after wetting v1 | 3300018839 |
| Arid desert | Soil crust microbial communities from Colorado Plateau, Utah, USA - early stage, 3 min after wetting v1 | 3300018954 |
| Arid desert | Soil crust microbial communities from Colorado Plateau, Utah, USA - late stage, 3 min after wetting v1 | 3300018962 |
| Arid desert | Soil crust microbial communities from Colorado Plateau, Utah, USA - mid late stage, 3 min after wetting v1 | 3300019142 |
| Arid desert | Soil microbial communities from Anza Borrego desert, Southern California, United States - S3_20-13C | 3300021057 |
| Arid desert | Soil microbial communities from Anza Borrego desert, Southern California, United States - S1_5-13C | 3300021061 |
| Arid desert | Soil microbial communities from Anza Borrego desert, Southern California, United States - S1_10-13C | 3300021062 |
| Arid desert | Soil microbial communities from Anza Borrego desert, Southern California, United States - S3+v_5 | 3300024426 |
| Arid desert | Soil microbial communities from Anza Borrego desert, Southern California, United States - S3+v_20 | 3300024430 |
| Arid desert | Soil microbial communities from Mojave Desert, California, United States - 5NOC | 3300033988 |
| Arid desert | Biocrust microbial communities from Mojave Desert, California, United States - 20HNC | 3300033989 |
| Arid desert | Biocrust microbial communities from Mojave Desert, California, United States - 14HMC | 3300034000 |
| Arid desert | Biocrust microbial communities from Mojave Desert, California, United States - 16HMC | 3300034002 |
| Arid desert | Biocrust microbial communities from Mojave Desert, California, United States - 18HMC | 3300034003 |
| Arid desert | Biocrust microbial communities from Mojave Desert, California, United States - 22HNC | 3300034004 |
| Arid desert | Sub-biocrust soil microbial communities from Mojave Desert, California, United States - 26HNS | 3300034005 |
| Arid desert | Biocrust microbial communities from Mojave Desert, California, United States - 30SMC | 3300034006 |
| Arid desert | Biocrust microbial communities from Mojave Desert, California, United States - 32SMC | 3300034007 |
| Arid desert | Sub-biocrust soil microbial communities from Mojave Desert, California, United States - 40SMS | 3300034009 |
| Arid desert | Biocrust microbial communities from Mojave Desert, California, United States - 23HNC | 3300034024 |
| Arid desert | Biocrust microbial communities from Mojave Desert, California, United States - 36SMC | 3300034025 |
| Arid desert | Sub-biocrust soil microbial communities from Mojave Desert, California, United States - 42SMS | 3300034026 |
| Arid desert | Biocrust microbial communities from Mojave Desert, California, United States - 45SNC | 3300034027 |
| Arid desert | Biocrust microbial communities from Mojave Desert, California, United States - 46SNC | 3300034028 |
| Arid desert | Biocrust microbial communities from Mojave Desert, California, United States - 47SNC | 3300034029 |
| Arid desert | Biocrust microbial communities from Mojave Desert, California, United States - 49SNC | 3300034031 |
| Arid desert | Biocrust microbial communities from Mojave Desert, California, United States - 50SNC | 3300034032 |
| Arid desert | Sub-biocrust soil microbial communities from Mojave Desert, California, United States - 7HMS | 3300034131 |
| Arid desert | Biocrust microbial communities from Mojave Desert, California, United States - 11HMC | 3300034132 |
| Arid desert | Biocrust microbial communities from Mojave Desert, California, United States - 12HMC | 3300034133 |
| Arid desert | Biocrust microbial communities from Mojave Desert, California, United States - 24HNC | 3300034134 |
| Arid desert | Biocrust microbial communities from Mojave Desert, California, United States - 25HNC | 3300034135 |
| Arid desert | Sub-biocrust soil microbial communities from Mojave Desert, California, United States - 29HNS | 3300034136 |
| Arid desert | Biocrust microbial communities from Mojave Desert, California, United States - 39SMC | 3300034137 |
| Arid desert | Biocrust microbial communities from Mojave Desert, California, United States - 51SNC | 3300034138 |
| Arid desert | Biocrust microbial communities from Mojave Desert, California, United States - 52SNC | 3300034139 |
| Arid desert | Biocrust microbial communities from Mojave Desert, California, United States - 53SNC | 3300034140 |
| Arid desert | Biocrust microbial communities from Mojave Desert, California, United States - 54SNC | 3300034141 |
| Arid desert | Biocrust microbial communities from Mojave Desert, California, United States - 55SNC | 3300034142 |
| Arid desert | Sub-biocrust soil microbial communities from Mojave Desert, California, United States - 57SNS | 3300034143 |
| Arid desert | Sub-biocrust soil microbial communities from Mojave Desert, California, United States - 59SNS | 3300034145 |
| Arid desert | Sub-biocrust soil microbial communities from Mojave Desert, California, United States - 60SNS | 3300034146 |
| Arid desert | Sub-biocrust soil microbial communities from Mojave Desert, California, United States - 9HMS | 3300034172 |
| Arid desert | Biocrust microbial communities from Mojave Desert, California, United States - 21HNC | 3300034173 |
| Arid desert | Sub-biocrust soil microbial communities from Mojave Desert, California, United States - 28HNS | 3300034174 |
| Arid desert | Biocrust microbial communities from Mojave Desert, California, United States - 35SMC | 3300034175 |
| Arid desert | Biocrust microbial communities from Mojave Desert, California, United States - 17HMC | 3300034220 |
| Arid desert | Biocrust microbial communities from Mojave Desert, California, United States - 33SMC | 3300034221 |
| Arid desert | Biocrust microbial communities from Mojave Desert, California, United States - 34SMC | 3300034236 |
| Arid desert | Sub-biocrust soil microbial communities from Mojave Desert, California, United States - 43SMS | 3300034251 |
| Arid desert | Biocrust microbial communities from Mojave Desert, California, United States - 19HNC | 3300034376 |
| Arid desert | Sub-biocrust soil microbial communities from Mojave Desert, California, United States - 27HNS | 3300034377 |
| Arid desert | Biocrust microbial communities from Mojave Desert, California, United States - 13HMC | 3300034391 |
| Arid desert | Sub-biocrust soil microbial communities from Mojave Desert, California, United States - 8HMS | 3300034392 |
| Arid desert | Sub-biocrust soil microbial communities from Mojave Desert, California, United States - 10HMS | 3300034393 |
| Arid desert | Sub-biocrust soil microbial communities from Mojave Desert, California, United States - 56SNS | 3300034402 |
| Arid desert | Soil microbial communities from Mojave Desert, California, United States - 1NOC | 3300034687 |
| Arid desert | Sub-biocrust soil microbial communities from Mojave Desert, California, United States - 44SMS | 3300034760 |
| Arid desert | Sub-biocrust soil microbial communities from Mojave Desert, California, United States - 41SMS | 3300034779 |
| Arid desert | Biocrust microbial communities from Mojave Desert, California, United States - 31SMC | 3300034781 |
| Arid desert | Soil microbial communities from Joshua Tree National Park, Mojave Desert, California, United States - 20181207_30 | 3300035019 |
| Hot springs | Hot spring microbial communities from Yellowstone Bath Hot Springs, Wyoming, USA - Filamentous sample | 2007309000 |
| Hot springs | 3_050719R | 2010170001 |
| Hot springs | 4_050719Q | 2010170002 |
| Hot springs | Hot spring microbial communities from Yellowstone National Park, Wyoming, USA - YNP6 White Creek Site 3 | 2013515000 |
| Hot springs | Hot spring microbial communities from Yellowstone National Park, Wyoming, USA - YNP1 Alice Springs, Crater Hills | 2014031002 |
| Hot springs | Hot spring microbial communities from Yellowstone National Park, Wyoming, USA - YNP2 Nymph Lake 10 | 2015219001 |
| Hot springs | Hot spring microbial communities from Yellowstone National Park, Wyoming, USA - YNP16 Fairy Spring Red Layer | 2016842003 |
| Hot springs | Hot spring microbial communities from Yellowstone National Park, Wyoming, USA - YNP6 White Creek Site 3 | 2022920004 |
| Hot springs | Hot spring microbial communities from Yellowstone National Park, Wyoming, USA - YNP13 Bechler Spring | 2022920006 |
| Hot springs | Hot spring microbial communities from One Hundred Springs Plain, Yellowstone National Park, Wyoming, USA - YNP14 OSP Spring | 2022920007 |
| Hot springs | Hot spring microbial communities from Yellowstone National Park, Wyoming, USA - YNP1 Alice Springs, Crater Hills | 2022920009 |
| Hot springs | Hot spring microbial communities from Yellowstone National Park, Wyoming, USA - YNP9 Dragon Spring, Norris Geyser Basin | 2022920010 |
| Hot springs | Hot spring microbial communities from Yellowstone National Park, Wyoming, USA - YNP11 Octopus Springs | 2022920012 |
| Hot springs | Hot spring microbial communities from Yellowstone National Park, Wyoming, USA - YNP15 Mushroom Spring | 2022920016 |
| Hot springs | Hot spring microbial communities from Yellowstone National Park, Wyoming, USA - YNP19 Cistern Spring | 2022920017 |
| Hot springs | Hot spring microbial communities from Yellowstone National Park, Wyoming, USA - YNP18 Washburn Springs #1 | 2022920019 |
| Hot springs | Hot spring microbial communities from Yellowstone National Park, Wyoming, USA - YNP20 Bath Lake Vista Annex - Purple-Sulfur Mats | 2022920020 |
| Hot springs | Sample 300 | 2025206004 |
| Hot springs | Hot spring sediment microbial communities from Great Boiling Spring, Nevada - surface sediment | 2053563014 |
| Hot springs | Hot spring microbial communities from Elkhorn Slough, Monterey Bay, USA - CD2A | 3300000347 |
| Hot springs | Hot spring microbial communities from Elkhorn Slough, Monterey Bay, USA - MD6A | 3300000353 |
| Hot springs | Hot spring microbial communities from Elkhorn Slough, Monterey Bay, USA - MD2A | 3300000919 |
| Hot springs | Hot spring microbial communities from Mammoth Springs in Yellowstone National Park, Montana, USA - YNG_F_2009 | 3300002486 |
| Hot springs | Hot spring microbial communities from Dewar Creek, Canada | 3300002851 |
| Hot springs | Hot springs microbial communities from Mammoth Hot Springs, Yellowstone National Park, Wyoming, USA-MHS Pond Facies_Gas lift | 3300002920 |
| Hot springs | Hot spring sediment microbial communities from Chocolate Pots, Yellowstone National Park, Wyoming that are Fe(III) reducing sample CP Core 1 1cm | 3300003891 |
| Hot springs | Hot spring sediment microbial communities from Chocolate Pots, Yellowstone National Park, Wyoming that are Fe(III) reducing - CP Core 2, 1cm | 3300003892 |
| Hot springs | Hot spring water microbial communities from Wilbur Hot Springs, California, USA - Wilbur Geyser tube - 6/17/14 | 3300003902 |
| Hot springs | Hot spring thermophilic microbial communities from Obsidian Pool, Yellowstone National Park, USA - OP-RAMG-01 (SPADES assembly) | 3300005573 |
| Hot springs | Hot spring microbial communities from Yellowstone National Park, Wyoming, USA - YNP10 Narrow Gauge | 3300005620 |
| Hot springs | Hot spring microbial communities from Yellowstone National Park, Wyoming, USA - YNP10 Narrow Gauge | 3300005621 |
| Hot springs | Hot spring microbial communities from Yellowstone National Park, Wyoming, USA - YNP2 Nymph Lake 10 | 3300005623 |
| Hot springs | Hot spring microbial communities from Yellowstone National Park, Wyoming, USA - YNP2 Nymph Lake 10 | 3300005624 |
| Hot springs | Sediment microbial communities of hot springs in Rotorua, New Zealand Wai-O-Tapu hot spring, NZ1 | 3300005798 |
| Hot springs | Sediment microbial communities of hot springs in Rotorua, New Zealand Waimangu hot spring, NZ8 | 3300005799 |
| Hot springs | Hot spring microbial streamer communities from Conch Spring, Yellowstone National Park, USA - CON_C (SPADES assembly) | 3300005854 |
| Hot springs | Hot spring sediment microbial communities from Joseph's Coat, Yellowstone National Park, USA - JC3_ASED (SPADES assembly) | 3300005856 |
| Hot springs | Microbial communities from hot springs in Navsari district, Gujarat, India - Unai | 3300007847 |
| Hot springs | Microbial communities from hot springs in Panchmahal district, Gujarat, India - Tuva | 3300007848 |
| Hot springs | Combined Assembly of Gp0139325, Gp0139347, Gp0139348 | 3300009943 |
| Hot springs | Combined Assembly of Gp0139326, Gp0139349, Gp0139350, Gp0139351 | 3300009945 |
| Hot springs | Sediment microbial community from Chocolate Pots hot springs, Yellowstone National Park, Wyoming, USA. Combined Assembly of Gp0156111, Gp0156114, Gp0156117 | 3300010938 |
| Hot springs | Hot spring water viral communities from Western Cape, South Africa - Brandvlei | 3300012978 |
| Hot springs | Hot spring microbial communities from Little Hot Creek, USA to study Microbial Dark Matter (Phase II) - LHC4sed_matched (SPAdes) | 3300025092 |
| Hot springs | Hot spring sediment microbial communities from Zodletone spring, Oklahoma to study Microbial Dark Matter (Phase II) - Zodletone Spring source 0.5m metaG (SPAdes) | 3300025100 |
| Hot springs | Hot spring microbial communities from South Africa to study Microbial Dark Matter (Phase II) - Sagole hot spring metaG (SPAdes) | 3300025116 |
| Hot springs | Hot spring microbial communities from Beatty, Nevada to study Microbial Dark Matter (Phase II) - OV2 TP3 (SPAdes) | 3300025157 |
| Hot springs | Hot spring sediment bacterial and archeal communities from British Columbia, Canada, to study Microbial Dark Matter (Phase II) - Dewar Creek DC9 2012 metaG (SPAdes) | 3300025161 |
| Hot springs | Hot spring sediment bacterial and archeal communities from British Columbia, Canada, to study Microbial Dark Matter (Phase II) - Larsen N4 metaG (SPAdes) | 3300025310 |
| Hot springs | Hot spring and microbial mat streamer communities from Octopus Spring Streamers, Yellowstone National Park, USA - OCT_B (SPAdes) | 3300025440 |
| Hot springs | Hot spring microbial communities from Elkhorn Slough, Monterey Bay, USA - MD2A | 3300025717 |
| Hot springs | Hot spring microbial mat communities from Yellowstone National Park, Wyoming, USA - ECH_C virus_MetaG (SPAdes) | 3300026623 |
| Hot springs | Hot spring thermophilic microbial communities from Obsidian Pool, Yellowstone National Park, USA - site 1 bottle 13 (SPAdes) | 3300026776 |
| Hot springs | Hot spring thermophilic microbial communities from Obsidian Pool, Yellowstone National Park, USA - site 3 bottle 8 (SPAdes) | 3300026781 |
| Hot springs | Hot spring microbial mat communities from Yellowstone National Park, Wyoming, USA - ECH_B nyco_MetaG (SPAdes) | 3300026821 |
| Hot springs | Hot spring sediment microbial communities from Yellowstone National Park, WY, United States - YNP-CB-003-1 | 3300028675 |
| Hot springs | Hot spring microbial mat communities from Yellowstone National Park, WY, United States - YNP-CB-006-1 | 3300028735 |
| Hot springs | Hot spring sediment microbial communities from Yellowstone National Park, WY, United States - YNP-CB-019-1 | 3300031463 |
| Hot springs | Hot spring phototrophic mat microbial communities from Octopus Spring, Yellowstone National Park, Wyoming, United States - 20060914_OS12-60 | 3300031509 |
| Hot springs | Extremophilic microbial mat communities from Washburn Hot Springs, YNP, Wyoming, USA - WHS_1_MG | 3300031749 |
| Hot springs | Hot spring phototrophic mat microbial communities from Octopus Spring, Yellowstone National Park, Wyoming, United States - 20090730_OS65 | 3300031950 |
| Hot springs | Hot spring phototrophic mat microbial communities from Mushroom Spring, Yellowstone National Park, Wyoming, United States - 20090730_MS55 | 3300031966 |
| Hot springs | Hot spring phototrophic mat microbial communities from Octopus Spring, Yellowstone National Park, Wyoming, United States - 20090730_OS60 | 3300032049 |
| Hot springs | Hot spring phototrophic mat microbial communities from Mushroom Spring, Yellowstone National Park, Wyoming, United States - 20090730_MS50 | 3300032058 |
| Hot springs | Hot spring water microbial communities from Geyser Creek Basin, Yellowstone National Park, WY, United States - GCR.EP_P | 3300033431 |
| Hot springs | Hot spring water microbial communities from Norris Geyser Basin, Yellowstone National Park, WY, United States - NOR.PS_P | 3300033484 |
| Hot springs | Hot spring sediment microbial communities from Norris-Mammoth Corridor, Yellowstone National Park, WY, United States - NMC.RSN_S | 3300036468 |
| Hot springs | Hot spring microbial communities from Yellowstone National Park, Wyoming, USA - Bijah Spring | 3300038505 |
| Polar environments | soil microbial communities from McMurdo Dry Valleys (Wright Valley), Antarctica | 2170459001 |
| Polar environments | Polar desert microbial communities from Antarctic Dry Valleys - UQ255 | 3300002548 |
| Polar environments | Arctic soil microbial communities form glacier forefield, Midre Lovenbreen, Svalbard, Norway (Sample 9 - S13.3.40.a - transect 3, age 29 years, surface depth). | 3300011176 |
| Polar environments | Arctic soil microbial communities form glacier forefield, Midre Lovenbreen, Svalbard, Norway (Sample 16 - S13.2.60.1.a - transect 2, repeat 1, age 113 years, surface depth) | 3300011191 |
| Polar environments | Arctic soil microbial communities form glacier forefield, Midre Lovenbreen, Svalbard, Norway (Sample 17 - S13.2.60.2.a - transect 2, repeat 2, age 113 years, surface depth) | 3300011196 |
| Polar environments | Arctic soil microbial communities form glacier forefield, Midre Lovenbreen, Svalbard, Norway (Sample 3 - S13.3.20.a - transect 3, age 3 years, surface depth). | 3300011199 |
| Polar environments | Arctic soil microbial communities form glacier forefield, Midre Lovenbreen, Svalbard, Norway (Sample 6 - S13.3.30.a - transect 3, age 5 years, surface depth) | 3300011200 |
| Polar environments | Arctic soil microbial communities form glacier forefield, Midre Lovenbreen, Svalbard, Norway (Sample 10 - S13.1.50.a - transect 1, age 50 years, surface depth). | 3300011227 |
| Polar environments | Arctic soil microbial communities form glacier forefield, Midre Lovenbreen, Svalbard, Norway (Sample 1 - S13.2.10.a - transect 2, age 0 years, surface depth) | 3300011239 |
| Polar environments | Glacer surface microbial communities from an Arctic cyroconite hole, Midre Lovenbreen, Svalbard, Norway (Sample 22) | 3300011246 |
| Polar environments | Polar desert sand microbial communities from Dry Valleys, Antarctica - metaG UQ469 (22.06) | 3300012183 |
| Polar environments | Polar desert sand microbial communities from Dry Valleys, Antarctica - metaG UQ134 (22.06) | 3300012184 |
| Polar environments | Polar desert sand microbial communities from Dry Valleys, Antarctica - metaG UQ353 (21.06) | 3300012185 |
| Polar environments | Polar desert sand microbial communities from Dry Valleys, Antarctica - metaG UQ416 (21.06) | 3300012186 |
| Polar environments | Polar desert sand microbial communities from Dry Valleys, Antarctica - metaG UQ448 (21.06) | 3300012187 |
| Polar environments | Arctic soils microbial communities. Combined Assembly of 23 SPs | 3300012668 |
| Polar environments | Arctic sediment microbial communities from supraglacial cryoconite, Rabots glacier, Tarfala, Sweden (Sample Rb cryoconite) | 3300015024 |
| Polar environments | Arctic soil microbial communities from a glacier forefield, Russell Glacier, Kangerlussuaq, Greenland (Sample G5B, Northern proglacial tributary margin, adjacent to top of river) | 3300015061 |
| Polar environments | Arctic soil microbial communities from a glacier forefield, Storglaciären, Tarfala, Sweden (Sample st-3b, vegetated patch on medial moraine) | 3300015063 |
| Polar environments | Arctic soil microbial communities from a glacier forefield, Russell Glacier, Kangerlussuaq, Greenland (Sample G7A, Adjacent to main proglacial river, mid transect (Watson river)) | 3300015067 |
| Polar environments | Arctic soil microbial communities from a glacier forefield, Russell Glacier, Kangerlussuaq, Greenland (Sample G8C, Adjacent to main proglacial river, end of transect (Watson river)) | 3300015068 |
| Polar environments | Arctic soil microbial communities from a glacier forefield, Russell Glacier, Kangerlussuaq, Greenland (Sample G4C, Ice margin, adjacent to proglacial lake | 3300015069 |
| Polar environments | Arctic soil microbial communities from a glacier forefield, Russell Glacier, Kangerlussuaq, Greenland (Sample G5C, Northern proglacial tributary margin, adjacent to top of river) | 3300015075 |
| Polar environments | Arctic soil microbial communities from a glacier forefield, Storglaciären, Tarfala, Sweden (Sample st-11a, vegetated hydrological feature) | 3300015078 |
| Polar environments | Arctic soil microbial communities from a glacier forefield, Storglaciären, Tarfala, Sweden (Sample st-6b, vegetation/snow interface) | 3300015079 |
| Polar environments | Arctic soil microbial communities from a glacier forefield, Storglaciären, Tarfala, Sweden (Sample st-11c, vegetated hydrological feature) | 3300015082 |
| Polar environments | Arctic soil microbial communities from a glacier forefield, Russell Glacier, Kangerlussuaq, Greenland (Sample G1C, Ice margin) | 3300015083 |
| Polar environments | Arctic soil microbial communities from a glacier forefield, Storglaciären, Tarfala, Sweden (Sample st-5a, rocky medial moraine) | 3300015084 |
| Polar environments | Arctic soil microbial communities from a glacier forefield, Russell Glacier, Kangerlussuaq, Greenland (Sample G4B, Ice margin, adjacent to proglacial lake) | 3300015085 |
| Polar environments | Arctic soil microbial communities from a glacier forefield, Russell Glacier, Kangerlussuaq, Greenland (Sample G8A, Adjacent to main proglacial river, end of transect (Watson river)) | 3300015089 |
| Polar environments | Arctic soil microbial communities from a glacier forefield, Russell Glacier, Kangerlussuaq, Greenland (Sample G5A, Northern proglacial tributary margin, adjacent to top of river) | 3300015090 |
| Polar environments | Arctic soil microbial communities from a glacier forefield, Russell Glacier, Kangerlussuaq, Greenland (Sample G3C, Proglacial river margin, by glacier terminus) | 3300015159 |
| Polar environments | Arctic soil microbial communities from a glacier forefield, Russell Glacier, Kangerlussuaq, Greenland (Sample G1B, Ice margin) | 3300015161 |
| Polar environments | Arctic soil microbial communities from a glacier forefield, Storglaciären, Tarfala, Sweden (Sample st-4c, rock/ice/stream interface) | 3300015162 |
| Polar environments | Arctic soil microbial communities from a glacier forefield, Storglaciären, Tarfala, Sweden (Sample st-4b, rock/ice/stream interface) | 3300015164 |
| Polar environments | Arctic soil microbial communities from a glacier forefield, Russell Glacier, Kangerlussuaq, Greenland (Sample G3A, Proglacial river margin, by glacier terminus) | 3300015165 |
| Polar environments | Arctic soil microbial communities from a glacier forefield, Russell Glacier, Kangerlussuaq, Greenland (Sample G4A, Ice margin, adjacent to proglacial lake) | 3300015168 |
| Polar environments | Arctic soil microbial communities from a glacier forefield, Storglaciären, Tarfala, Sweden (Sample st-4a, rock/ice/stream interface) | 3300015190 |
| Polar environments | Arctic soil microbial communities from a glacier forefield, Rabots glacier, Tarfala, Sweden (Sample Rb6, proglacial stream) | 3300015193 |
| Polar environments | Arctic soil microbial communities from a glacier forefield, Rabots glacier, Tarfala, Sweden (Sample Rb1c, glacier snout) | 3300015194 |
| Polar environments | Arctic soil microbial communities from a glacier forefield, Russell Glacier, Kangerlussuaq, Greenland (Sample G2C, Ice surface) | 3300015196 |
| Polar environments | Arctic soil microbial communities from a glacier forefield, Russell Glacier, Kangerlussuaq, Greenland (Sample G6B, Proglacial plain, adjacent to northern proglacial tributary) | 3300015197 |
| Polar environments | Arctic soil microbial communities from a glacier forefield, Russell Glacier, Kangerlussuaq, Greenland (Sample G2A, Ice surface) | 3300015198 |
| Polar environments | Arctic soil microbial communities from a glacier forefield, Storglaciären, Tarfala, Sweden (Sample st-2c, rock/snow interface) | 3300015199 |
| Polar environments | Arctic sediment microbial communities from supraglacial cryoconite, Storglaciären, Tarfala, Sweden (Sample st-12a, ablation zone cryoconite) | 3300015202 |
| Polar environments | Arctic soil microbial communities from a glacier forefield, Russell Glacier, Kangerlussuaq, Greenland (Sample G2B, Ice surface) | 3300015204 |
| Polar environments | Arctic soil microbial communities from a glacier forefield, Russell Glacier, Kangerlussuaq, Greenland (Sample G8B, Adjacent to main proglacial river, end of transect (Watson river)) | 3300015206 |
| Polar environments | Arctic soil microbial communities from a glacier forefield, Russell Glacier, Kangerlussuaq, Greenland (Sample G3B, Proglacial river margin, by glacier terminus) | 3300015209 |
| Polar environments | Saline lake microbial communities from Rauer Lake, Antarctica, in enrichment culture - Antartic Rauer Lake 3 Metagenome Rauer3 (SPAdes) | 3300025343 |
| Polar environments | Polar desert microbial communities from Antarctic Dry Valleys - UQ493 (SPAdes) | 3300027414 |
| Polar environments | Polar desert microbial communities from Antarctic Dry Valleys - UQ313 (SPAdes) | 3300027415 |
| Polar environments | Polar desert microbial communities from Antarctic Dry Valleys - UQ255 (SPAdes) | 3300027618 |
| Polar environments | Polar desert microbial communities from Antarctic Dry Valleys - UQ889 (SPAdes) | 3300027638 |
| Polar environments | Marine microbial communities from Northeast Subartic Pacific Ocean, Canada - LP_J_2011_P26_500m | 3300028192 |
| Polar environments | Marine microbial communities from Northeast Subartic Pacific Ocean, Canada - LP_J_2011_P26_10m | 3300028194 |
| Polar environments | Marine microbial communities from Northeast Subartic Pacific Ocean, Canada - LP_J_2011_P26_2000m | 3300028487 |
| Polar environments | Marine microbial communities from Northeast Subartic Pacific Ocean, Canada - LP_J_2015_P26_500m | 3300028535 |
| Submarine Volcano | Marine microbial communities from a deep-sea hydrothermal vent at Loihi Seamount, Hawaii | 3300000098 |
| Submarine Volcano | Marine microbial communities from a deep-sea hydrothermal vent at Loihi Seamount, Hawaii | 3300000272 |
| Submarine Volcano | Marine microbial communities from a deep-sea hydrothermal vent at Loihi Seamount, Hawaii | 3300000327 |
| Submarine Volcano | Hydrothermal vent microbial communities from the Southwest Indian Ridge | 3300001391 |
| Submarine Volcano | Hydrothermal vent plume microbial communities from Kilo Moana, Pacific Ocean, of black smokers | 3300001515 |
| Submarine Volcano | Hydrothermal vent plume microbial communities from Tahi Moana, Pacific Ocean, of black smokers | 3300001516 |
| Submarine Volcano | Hydrothermal vent plume microbial communities from Tui Malila, Pacific Ocean, of black smokers | 3300001522 |
| Submarine Volcano | Black smokers hydrothermal plume microbial communities from Abe, Lau Basin, Pacific Ocean (2) | 3300001524 |
| Submarine Volcano | Black smokers hydrothermal plume microbial communities from Mariner, Lau Basin, Pacific Ocean -IDBA | 3300001678 |
| Submarine Volcano | Black smokers hydrothermal plume microbial communities from Tahi Moana, Lau Basin, Pacific Ocean | 3300001679 |
| Submarine Volcano | Black smokers hydrothermal plume microbial communities from Kilo Moana, Pacific Ocean | 3300001680 |
| Submarine Volcano | Black smokers hydrothermal plume microbial communities from Abe, Lau Basin, Pacific Ocean | 3300001681 |
| Submarine Volcano | Hydrothermal vent plume microbial communities from Guaymas Basin, Pacific Ocean | 3300001683 |
| Submarine Volcano | Hydrothermal vent plume microbial communities from the Cayman Rise, Cayman Islands | 3300001707 |
| Submarine Volcano | Hydrothermal vent plume microbial communities from the Cayman Rise, Cayman Islands | 3300001763 |
| Submarine Volcano | Hydrothermal vent plume microbial communities from the Cayman Rise, Cayman Islands | 3300001768 |
| Submarine Volcano | Hydrothermal vent plume microbial communities from the Cayman Rise, Cayman Islands | 3300001780 |
| Submarine Volcano | Hydrothermal vent plume microbial communities from the Cayman Rise, Cayman Islands | 3300001781 |
| Submarine Volcano | Hydrothermal vent plume microbial communities from the Cayman Rise, Cayman Islands | 3300001783 |
| Submarine Volcano | Hydrothermal vent plume microbial communities from the Cayman Rise, Cayman Islands | 3300001835 |
| Submarine Volcano | Hydrothermal vent plume microbial communities from the Cayman Rise, Cayman Islands | 3300001840 |
| Submarine Volcano | Hydrothermal vent plume microbial communities from the Cayman Rise, Cayman Islands | 3300001845 |
| Submarine Volcano | Hydrothermal vent plume microbial communities from the Cayman Rise, Cayman Islands | 3300001921 |
| Submarine Volcano | Marine sediment microbial communities from the Hellenic Volcanic Arc | 3300002242 |
| Submarine Volcano | Diffuse hydrothermal flow volcanic vent microbial communities from Axial Seamount, northeast Pacific ocean | 3300003514 |
| Submarine Volcano | Diffuse hydrothermal flow volcanic vent microbial communities from Axial Seamount, northeast Pacific ocean | 3300003537 |
| Submarine Volcano | Diffuse hydrothermal flow volcanic vent microbial communities from Axial Seamount, northeast Pacific ocean | 3300003538 |
| Submarine Volcano | Diffuse hydrothermal flow volcanic vent microbial communities from Axial Seamount, northeast Pacific ocean | 3300003540 |
| Submarine Volcano | Diffuse hydrothermal flow volcanic vent microbial communities from Axial Seamount, northeast Pacific ocean | 3300003542 |
| Submarine Volcano | Diffuse hydrothermal flow volcanic vent microbial communities from Axial Seamount, northeast Pacific ocean | 3300003543 |
| Submarine Volcano | Hydrothermal vent plume microbial communities from the Mid Cayman Rise - Piccard2013-Plume | 3300003690 |
| Submarine Volcano | Black Smoker Hydrothermal vent Microbial communities from Manus Basin , Bismarck Sea | 3300003873 |
| Submarine Volcano | Black smoker hydrothermal vent sediment microbial communities from the Guaymas Basin, Mid-Atlantic Ridge, South Atlantic Ocean | 3300003885 |
| Submarine Volcano | Diffuse hydrothermal vent microbial communities from Menez Gwen hydrothermal field, Mid Atlantic ridge | 3300003979 |
| Submarine Volcano | Microbial community analysis of hydrothermal vent diffuse flow samples from Mid-Cayman Rise, Pacific Ocean | 3300005749 |
| Submarine Volcano | Microbial community analysis of hydrothermal vent diffuse flow samples from Mid-Cayman Rise, Pacific Ocean | 3300005753 |
| Submarine Volcano | Microbial community analysis of hydrothermal vent diffuse flow samples from Mid-Cayman Rise, Pacific Ocean | 3300005814 |
| Submarine Volcano | Microbial community analysis of hydrothermal vent diffuse flow samples from Mid-Cayman Rise, Pacific Ocean | 3300005816 |
| Submarine Volcano | Diffuse hydrothermal flow volcanic vent microbial communities from Axial Seamount, northeast Pacific ocean | 3300006083 |
| Submarine Volcano | Diffuse hydrothermal flow volcanic vent microbial communities from Axial Seamount, northeast Pacific ocean | 3300006611 |
| Submarine Volcano | Diffuse hydrothermal flow volcanic vent microbial communities from Axial Seamount, northeast Pacific ocean | 3300006612 |
| Submarine Volcano | Hydrothermal vent microbial communities from Crab Spa hydrothermal vent, East Pacific Rise | 3300007279 |
| Submarine Volcano | Diffuse hydrothermal flow volcanic vent microbial communities from Axial Seamount, northeast Pacific ocean | 3300007756 |
| Submarine Volcano | Diffuse hydrothermal flow volcanic vent microbial communities from Axial Seamount, northeast Pacific ocean | 3300007771 |
| Submarine Volcano | Diffuse hydrothermal flow volcanic vent microbial communities from Axial Seamount, northeast Pacific ocean | 3300007776 |
| Submarine Volcano | Diffuse hydrothermal flow volcanic vent microbial communities from Axial Seamount, northeast Pacific ocean | 3300007777 |
| Submarine Volcano | Deep subsurface microbial communities from various oceans to uncover new lineages of life (NeLLi) | 3300009481 |
| Submarine Volcano | Microbial community analysis of hydrothermal vent diffuse flow samples from Mid-Cayman Rise, Pacific Ocean | 3300009485 |
| Submarine Volcano | Deep subsurface microbial communities from various oceans to uncover new lineages of life (NeLLi) | 3300009702 |
| Submarine Volcano | Deep subsurface microbial communities from various oceans to uncover new lineages of life (NeLLi) | 3300009703 |
| Submarine Volcano | Inactive Hydrothermal Chimney Metagenome Study | 3300010967 |
| Submarine Volcano | Inactive Hydrothermal Chimney Metagenome Study | 3300010968 |
| Submarine Volcano | Inactive Hydrothermal Chimney Metagenome Study | 3300010969 |
| Submarine Volcano | Deep subsurface microbial communities from various oceans to uncover new lineages of life (NeLLi) | 3300011013 |
| Submarine Volcano | Deep-sea hydrothermal vent sediment bacterial and viral communities from Southwest Indian Ocean | 3300014662 |
| Submarine Volcano | Microbial communities from sediments and microbial mats in various locations | 3300021490 |
| Submarine Volcano | Microbial communities from sediments and microbial mats in various locations | 3300021491 |
| Submarine Volcano | Microbial communities from sediments and microbial mats in various locations | 3300021494 |
| Submarine Volcano | Microbial communities from sediments and microbial mats in various locations | 3300021504 |
| Submarine Volcano | Microbial communities from sediments and microbial mats in various locations | 3300021505 |
| Submarine Volcano | Microbial communities from sediments and microbial mats in various locations | 3300021580 |
| Submarine Volcano | Microbial communities from sediments and microbial mats in various locations | 3300021589 |
| Submarine Volcano | Deep subsurface microbial communities from various oceans to uncover new lineages of life (NeLLi) | 3300024344 |
| Submarine Volcano | Marine microbial communities from hydrothermal vents in the Atlantic and Pacific Ocean | 3300026531 |
| Submarine Volcano | Marine microbial communities from hydrothermal vents in the Atlantic and Pacific Ocean | 3300028417 |
| Submarine Volcano | Deep-sea hydrothermal vent sediment bacterial and viral communities from Southwest Indian Ocean | 3300038346 |
| Caves | Subaerial biofilm microbial communities from sulfidic caves, Italy, that are extremely acidic - Ramo Sulfureo RS9 | 2199352029 |
| Caves | Speleothem and rock wall surfaces microbial communities from Kartchner Caverns, Benson, Arizona, USA | 2199352031 |
| Caves | Speleothem and rock wall surfaces microbial communities from Kartchner Caverns, Benson, Arizona, USA | 2199352032 |
| Caves | Speleothem and rock wall surfaces microbial communities from Kartchner Caverns, Benson, Arizona, USA | 2199352033 |
| Caves | Speleothem and rock wall surfaces microbial communities from Kartchner Caverns, Benson, Arizona, USA | 2209111007 |
| Caves | Subaerial biofilm microbial communities from sulfidic caves, Italy, that are extremely acidic - Acquasanta AS5 | 3300000825 |
| Caves | Stalagmite microbial communities from Echo Passage, Kartchner Caverns, Arizona, USA | 3300004454 |
| Caves | White microbial mat communities from a basaltic lava cave in the Kipuka Kanohina Cave System on the Island of Hawaii, USA - MA170107-4 metaG | 3300019487 |
| Caves | Cave wall surface microbial communities from Maniniholo Cave, Kauai, Hawaii, United States - 20181024_15A | 3300035000 |
| Caves | Cave wall surface microbial communities from Maniniholo Cave, Kauai, Hawaii, United States - 20181031_20A | 3300035004 |
| Caves | Cave wall surface microbial communities from Makauwahi Cave, Kauai, Hawaii, United States - 20181107_10A | 3300035008 |
| Caves | Cave wall surface microbial communities from Waikapalae Cave, Hawaii, United States - 20190126_53 | 3300036446 |
| Caves | Cave wall surface microbial communities from Maniniholo Cave near Haena, Hawaii, United States - 20190131_40 | 3300037800 |
| Caves | Cave wall surface microbial communities from Waikapalae Cave, Hawaii, United States - 20190625_16 | 3300038554 |
| Caves | Rock surface microbial communities from Maniniholo Cave, near Haena, Kauai, HI, United States - 20190625_3 | 3300039303 |

**Supplementary Table 3**. **Raw data used to compare unassembled versus assembled results.**

Samples used for comparing ARG results in unassembled and assembled data. Five gut samples and five buccal mucosa were obtained from the HMP webpage (<https://downloads.hmpdacc.org/dacc/hhs/genome/microbiome/wgs/analysis/hmwgsqc>) and reads were used to annotate antibiotic resistance genes with DeepARG (Arango-Argoty et al., 2018) and BLASTx and then compared with the assembled data results from the same subjects (see Methods).

| **Body part** | **Subject ID** | **SRS Code** |
| --- | --- | --- |
| Gut | 159005010 | SRS023583 |
| Gut | 159247771 | SRS024009 |
| Gut | 159369152 | SRS023971 |
| Gut | 763961826 | SRS014683 |
| Gut | 246515023 | SRS023346 |
| Buccal mucosa | 370425937 | SRS052668 |
| Buccal mucosa | 764325968 | SRS049283 |
| Buccal mucosa | 604812005 | SRS046623 |
| Buccal mucosa | 246515023 | SRS023354 |
| Buccal mucosa | 809635352 | SRS065431 |

**Supplementary Table 4.** **Resistome characterization of the analyzed human samples.** For the different body parts included in this study, the percentage (%) of samples without antibiotic resistance genes (ARGs) obtained from dividing the number of samples without ARGs (N° Samples without ARGs) by the number of all the analysed samples (N° Total analysed samples) for the different body parts. It is also displayed the % of the total number of proteins that were considered as ARGs (bs≥70, id≥90% with CARD (Jia et al., 2017), ARG_ANNOT (Gupta et al., 2014) and RESFAMS (Gibson et al., 2015) databases) compared with the number of total analysed proteins (N° Total analysed proteins).

| Body site | No. Samples without ARGs | No. Total analysed samples | Samples without ARGs (%) | ARGs hits (bs≥70,  id≥90%) | N° Total analysed proteins | No. of ARGs/No. of total proteins (%) |
| --- | --- | --- | --- | --- | --- | --- |
| **Nares** | 13 | 94 | 13.83 | 938 | 1174770 | 0.08 |
| **Vagina** | 29 | 65 | 44.61 | 191 | 546877 | 0.03 |
| **Oral cavity** | 1 | 414 | 0.24 | 21819 | 63001299 | 0.03 |
| **Skin** | 2 | 47 | 4.25 | 642 | 1435242 | 0.04 |
| **Gut** | 0 | 151 | 0 | 5124 | 25549140 | 0.02 |
| TOTAL |  | **771** |  | **28714** | **91707328** |  |

**Supplementary Table 5.** **Different ARGs found in the human body.** Antibiotic resistance gene entries (ARG entry) from the database CARD (Jia et al., 2017), ARG_ANNOT (Gupta et al., 2014) and RESFAMS (Gibson et al., 2015) found in the studied samples with an identity ≥90% and a bit-score ≥70. ARG entries were grouped by their gene name (ARG) and the antibiotic class they confer resistance to, according to CARD database (Jia et al., 2017).

| **ARG entry** | **ARG** | **Antibiotic class** |
| --- | --- | --- |
| gi\|490429857\|ref\|WP_004301991_1\| | A2 | beta-lactamase |
| gi\|134047089\|ref\|YP_001102018_1\| | AAC(3)-VI | aminoglycoside |
| (AGly)aac6-Aph2:M13771:304-1743:1440 | aac6-aph2 | aminoglycoside |
| (AGly)aac6-Im:AF337947:1215-1751:537 | aac6-lm | aminoglycoside |
| (AGly)aadA1:M95287:3320-4111:792 | aadA1 | aminoglycoside |
| (AGly)aadA1-pm:JQ690540:7968-8798:831 |  |  |
| AJ584652_2_gene7_p01 | aadA2 | aminoglycoside |
| gb\|CAJ32491.1\|ARO:3002622\|aadA6/aadA10 | aadA6/aadA10 | aminoglycoside |
| (AGly)aadC:V01282:225-701:477 | aadD | aminoglycoside |
| (AGly)aadD:AF181950:3176-3946:771 |  |  |
| gi\|190410481\|ref\|YP_001965484_1\| | aadE | aminoglycoside |
| (AGly)aadS:NG_047380:101-964:864 | aadS | aminoglycoside |
| gi\|57651525\|ref\|YP_185623_1\| | ABC-transporter | multidrug |
| gi\|57651526\|ref\|YP_185624_1\| |  |  |
| (Bla)aci1:AJ007350:240-1094:855 | ACI-1 | beta-lactamase |
| gb\|AAC73565.1\|ARO:3004043\|Escherichia | AcrA | multidrug |
| gb\|ABG77965.1\|ARO:3004042\|Enterobacter |  |  |
| gb\|CAC41008.1\|ARO:3004041\|Klebsiella |  |  |
| gi\|485715312\|ref\|WP_001347297_1\| | AcrAB | multidrug |
| gb\|AAC73564.1\|ARO:3000216\|acrB | acrB | multidrug |
| gb\|AAC76297.1\|ARO:3000499\|acrE |  |  |
| gb\|BAA16344.1\|ARO:3000491\|acrD | acrD | aminoglycoside |
| NC_002695_1_915267_p01 |  |  |
| NC_007793_3913486_p01 |  |  |
| gb\|AAC76298.1\|ARO:3000502\|acrF | acrF | multidrug |
| NC_002952_2861052_p01 |  |  |
| NC_002953_2862818_p01 |  |  |
| gb\|AAC76296.1\|ARO:3000656\|acrS | acrS | multidrug |
| gb\|AAX14802.1\|ARO:3000781\|adeJ | adeJ | multidrug |
| gi\|113727\|sp\|P05364_1\|AMPC_ENTCL | AmpC | beta-lactamase |
| NC_002695_1_914045_p01 |  |  |
| (Bla)AmpC1_Ecoli:FN649414:2765051-2766355:1302 | AmpC1 | beta-lactamase |
| (Bla)AmpC2_Ecoli:CP002970:332756-333889:1134 | AmpC2 | beta-lactamase |
| (Bla)ampH:CP003785:4208384-4209544:1161 | ampH | beta-lactamase |
| (Bla)ampH_Ecoli:AP012030:395554-396711:1158 |  |  |
| gb\|CAA26199.1\|ARO:3004089\|ANT(3'')-IIa | aadA1 | aminoglycoside |
| gi\|479146460\|ref\|YP_007776889_1\| | aad(6) | aminoglycoside |
| gb\|AHE40557.1\|ARO:3002626\|ANT(6)-Ia |  |  |
| (AGly)ant6-Ia:AF330699:22-930:909 |  |  |
| (AGly)ant(9)-Ia:NG_047397:101-883:783 | ant(9)-Ia | aminoglycoside |
| (AGly)ant6-Ib:FN594949:27482-28339:858 | ant6-lb | aminoglycoside |
| gb\|AAW34150.1\|ARO:3004191\|APH(2'')-If | APH(2'')-If | aminoglycoside |
| (AGly)aph(2'')-Ig:NG_047407:101-1021:921 | APH(2'')-Ig | aminoglycoside |
| (AGly)aph(2'')-Ib:AF207840:272-1171:900 | APH(2'')-IIa | aminoglycoside |
| (AGly)aph(2'')-IIa:NG_047402:1-900:900 |  |  |
| (AGly)aph(2'')-Id:AF016483:131-1036:906 | APH(2'')-ld | aminoglycoside |
| gi\|32455908\|ref\|NP_862671_1\| | APH(3')-Ia | aminoglycoside |
| (AGly)aph(3'')-Ia:FJ172370:38668-39483:816 | APH(3'')-Ia | aminoglycoside |
| gi\|170576369\|ref\|XP_001893601_1\| |  |  |
| (AGly)strA:AB366441:22458-23261:804 | APH(3'')-Ib | aminoglycoside |
| AF024602_1_gene5_p01 |  | aminoglycoside |
| (AGly)aphA2:X57709:1-795:795 | APH(3')-Iia | aminoglycoside |
| gb\|CAA23892.1\|ARO:3002644\|APH(3')-IIa |  |  |
| (AGly)aph(3'')-III:V01547:535-1329:795 | APH(3'')-III | aminoglycoside |
| (AGly)aph4-Ia:V01499:231-1256:1026 | APH(4)-Ia | aminoglycoside |
| gi\|487835679\|ref\|WP_001909145_1\| | APH(6)-I | aminoglycoside |
| (AGly)strB:FJ474091:264-1100:837 | APH(6)-Id | aminoglycoside |
| (AGly)aph7:GG774704:686456-687373:918 | APH(7'') | aminoglycoside |
| (AGly)apH-Stph:HE579073:1778413-1779213:801 | APH-Stph | aminoglycoside |
| gb\|ATC67679.1\|ARO:3000838\|arlR | arlR | multidrug |
| gb\|ABD30512.1\|ARO:3000839\|arlS | arlS | multidrug |
| gb\|AAC76093.1\|ARO:3002986\|bacA | bacA | peptide |
| gb\|BAA15935.1\|ARO:3000828\|baeR | baeR | multidrug |
| NC_002695_1_916589_p01 |  |  |
| gb\|BAA15934.1\|ARO:3000829\|baeS | baeS | multidrug |
| (Bla)blaBRO-1:Z54180:84-1028:945 | blaBRO-1 | beta-lactamase |
| (Bla)blaBRO-2:Z54181:63-1007:945 | blaBRO-2 | beta-lactamase |
| gi\|110808074\|ref\|YP_691594_1\| | blaEC | beta-lactamase |
| (Fcd)far1:NC_005127:19072-19713:642 | blaFAR-1 | beta-lactamase |
| (Bla)blaI:NG_047499:101-481:381 | blaI | beta-lactamase |
| NC_010066_5774788_p01 |  |  |
| NC_010079_5776237_p01 |  |  |
| NC_010419_6155809_p01 |  |  |
| (Bla)blaOXA-209:JF268688:5408-6232:825 | blaOXA-209 | beta-lactamase |
| (Bla)blaOXA-347:JN086160:1583-2407:825 | blaOXA-347 | beta-lactamase |
| (Bla)blaOXA-85:AY227054:1-765:765 | blaOXA-85 | beta-lactamase |
| NC_002952_2858974_p01 | blaR1 | beta-lactamase |
| NC_005054_2598288_p01 |  |  |
| NC_005127_2610353_p01 |  |  |
| NC_005951_2853425_p01 |  |  |
| NC_007931_3978624_p01 |  |  |
| NC_009477_5169314_p01 |  |  |
| NC_010419_6155810_p01 |  |  |
| NC_011035_1_6447343_p01 |  |  |
| (Bla)blaSPU-1:GQ919044:1-882:882 | blaSPU-1 | beta-lactamase |
| (Bla)blaROB-1:AF022114:303-1220:918 | blaROB-1 | beta-lactamase |
| (Bla)blaZ:AB245469:2235-3080:861 | BlaZ | beta-lactamase |
| gb\|ABX30738.1\|ARO:3000621\|PC1 |  |  |
| NC_005054_2598287_p01 |  |  |
| NC_005127_2610354_p01 |  |  |
| NC_005951_2853409_p01 |  |  |
| NC_007931_3978604_p01 |  |  |
| NC_010063_5774822_p01 |  |  |
| gb\|AAA23018.1\|ARO:3004454\|Campylobacter | cat | chloramphenicol |
| gb\|AAB23649.1\|ARO:3004460\|Vibrio |  |  |
| gb\|AGR48358.1\|ARO:3004459\|Shigella |  |  |
| gi\|145301309\|ref\|YP_001144149_1\| |  |  |
| gi\|154799922\|emb\|CAO82974_1\| |  |  |
| NC_010427_6155852_p01 | catA9 | chloramphenicol |
| gb\|CAA37806.1\|ARO:3002684\|catII | catII | chloramphenicol |
| gi\|119633061\|ref\|YP_918951_1\| |  |  |
| gb\|AAB51421.1\|ARO:3002686\|catP | catP | chloramphenicol |
| gb\|CAA52904.1\|ARO:3002688\|catS | catS | chloramphenicol |
| (Bla)cblA:NG_047624:101-991:891 | cblA | beta-lactamase |
| gi\|390946537\|ref\|YP_006410297_1\| | CcrA-like_MBL-B1 | beta-lactamase |
| (Bla)cepA:FR688019:1-903:903 | cepA | beta-lactamase |
| gb\|AAA21532.1\|ARO:3003559\|cepA |  |  |
| gi\|492287460\|ref\|WP_005799525_1\| |  |  |
| gi\|53712542\|ref\|YP_098534_1\| |  |  |
| (Bla)cepA-29:U05884:556-1458:903 | cepA-29 | beta-lactamase |
| (Bla)cepA-44:U05885:556-1458:903 | cepA-44 | beta-lactamase |
| (Bla)cepA-49:U05886:404-1306:903 | cepA-49 | beta-lactamase |
| (Bla)CfiA1:AB087225:1-750:750 | CfiA1 | beta-lactamase |
| (Bla)cfiA4:AB087229:1-750:750 | cfiA4 | beta-lactamase |
| NC_009632_5316674_p01 | Cfr23 | multidrug |
| (Bla)cfxA:U38243:150-1115:966 | cfxA | beta-lactamase |
| gi\|496046347\|ref\|WP_008770854_1\| |  |  |
| (Bla)cfxA2:AF504910:1-966:966 | cfxA2 | beta-lactamase |
| gb\|AAD23513.1\|ARO:3003002\|CfxA2 |  |  |
| gi\|21327013\|gb\|AAM48121_1\|AF504914_1 |  |  |
| gi\|21327021\|gb\|AAM48125_1\|AF504918_1 |  |  |
| gb\|AAL79549.2\|ARO:3003003\|CfxA3 | cfxA3 | beta-lactamase |
| (Bla)cfxA4:AY769933:1-966:966 | cfxa4 | beta-lactamase |
| (Bla)cfxA5:AY769934:28-993:966 | cfxa5 | beta-lactamase |
| (Bla)cfxA6:GQ342996:798-1793:966 | cfxa6 | beta-lactamase |
| gi\|169797578\|ref\|YP_001715371_1\| | cmlA | multidrug |
| gi\|521232603\|ref\|YP_008167116_1\| |  |  |
| gb\|AFH35853.1\|ARO:3001328\|Escherichia |  |  |
| gb\|AAG03380.1\|ARO:3002703\|cmx | cmx | chloramphenicol |
| gi\|30387222\|ref\|NP_848198_1\| |  |  |
| gb\|BAB38260.1\|ARO:3000830\|cpxA | cpxA | multidrug |
| gb\|BAE77933.1\|ARO:3000518\|CRP | CRP | multidrug |
| gb\|ACI32877.1\|ARO:3002859\|dfrA14 | dfrA14 | diaminopyrimidine |
| gb\|ABG91835.1\|ARO:3002860\|dfrA17 | dfrA17 | diaminopyrimidine |
| (Tmt)dfrA17:AB126604:98-571:474 |  |  |
| gb\|AAO04716.1\|ARO:3002865\|dfrC | dfrC | diaminopyrimidine |
| gb\|AAD01868.1\|ARO:3002867\|dfrF | dfrF | diaminopyrimidine |
| (Tmt)dfrF:NG_047755:101-595:495 |  |  |
| gb\|BAE15963.1\|ARO:3002868\|dfrG | dfrG | diaminopyrimidine |
| gb\|BAB43543.1\|ARO:3002132\|DHA-1 | DHA-1 | beta-lactamase |
| NC_002695_1_913290_p01 | ECs1601 | MLS |
| gb\|BAA16547.1\|ARO:3000027\|emrA | emrA | fluoroquinolone |
| gb\|AAC75733.1\|ARO:3000074\|emrB | emrB | fluoroquinolone |
| NC_009641_5331371_p01 |  |  |
| NC_013450_8615150_p01 |  |  |
| NC_013450_8615214_p01 |  |  |
| NC_002695_1_915420_p01 | emrD | multidrug |
| gb\|CAA77936.1\|ARO:3004039\|Escherichia | emrE | multidrug |
| NC_002695_1_913273_p01 |  |  |
| gb\|BAA11236.1\|ARO:3000206\|emrK | emrK | tetracycline |
| NC_002695_1_915653_p01 |  |  |
| gb\|AAC75731.1\|ARO:3000516\|emrR | emrR | fluoroquinolone |
| gb\|BAA11237.1\|ARO:3000254\|emrY | emrY | fluoroquinolone |
| gb\|BAE78116.1\|ARO:3003576\|eptA | eptA | peptide |
| gb\|AKO71461.1\|ARO:3004608\|EreD | ereD | MLS |
| AJ313523_1_orf0_gene_p01 | Erm(33) | MLS |
| gb\|AAF86219.1\|ARO:3000375\|ErmB | ErmB | MLS |
| gi\|182684293\|ref\|YP_001836040_1\| |  |  |
| gi\|428160746\|ref\|YP_007078965_1\| |  |  |
| (MLS)erm(B):M11180:714-1451:738 |  |  |
| gb\|AAA98296.1\|ARO:3000250\|ErmC | ErmC | MLS |
| gi\|94995100\|ref\|YP_603198_1\| |  |  |
| (MLS)erm(C):M19652:988-1722:735 |  |  |
| NC_007209_3523503_p01 |  |  |
| NC_007792_3912746_p01 |  |  |
| NC_010685_6295744_p01 |  |  |
| gb\|AAA88675.1\|ARO:3000498\|ErmF | ErmF | MLS |
| (MLS)erm(F):M14730:241-1041:801 |  |  |
| gb\|AAC37034.1\|ARO:3000522\|ErmG | ErmG | MLS |
| M15332_gene_p01 |  |  |
| gb\|AAC36915.1\|ARO:3000593\|ErmQ | ErmQ | MLS |
| AM903082_gene3_p01 | ErmX | MLS |
| gb\|AAA98484.1\|ARO:3000596\|ErmX |  |  |
| (MLS)erm(CX):AF024666:2208-2969:762 |  |  |
| gb\|BAB36671.1\|ARO:3000832\|evgA | evgA | multidrug |
| gb\|AAC75429.1\|ARO:3000833\|evgS | evgS | multidrug |
| gb\|AAF40763.1\|ARO:3003961\|farA | farA | antibacterial |
| gb\|AAF40764.1\|ARO:3003962\|farB | farB | antibacterial |
| NC_010079_5776908_p01 | fmtC | peptide |
| (Fcyn)FosB:AHLO01000073:63139-63558:420 | fosB | fosfomycin |
| gb\|ANK04027.1\|ARO:3003838\|gadW | gadW | multidrug |
| gb\|BAE77778.1\|ARO:3000508\|gadX | gadX | multidrug |
| gb\|ADO96486.1\|ARO:3003953\|hmrM | hmrM | fluoroquinolone |
| gb\|BAB35162.1\|ARO:3000676\|H-NS | H-NS | multidrug |
| gb\|AAD08227.1\|ARO:3003964\|hp1181 | hp1181 | multidrug |
| gb\|EGE18576.1\|ARO:3004466\|ICR-Mc | ICR-Mc | peptide |
| gb\|AAY32951.1\|ARO:3002837\|lnuC | InuC | MLS |
| (MLS)lnu(C):AY928180:1150-1644:495 |  |  |
| gb\|AAL05554.1\|ARO:3003206\|lsaE | IsaE | multidrug |
| gb\|AAC73788.1\|ARO:3003841\|kdpE | kdpE | aminoglycoside |
| gb\|BAH63251.1\|ARO:3004580\|Klebsiella | KpnE | multidrug |
| gb\|BAH63252.1\|ARO:3004583\|Klebsiella | KpnF | multidrug |
| gb\|EHL92831.1\|ARO:3004588\|Klebsiella | KpnG | multidrug |
| gb\|AAW38464.1\|ARO:3004572\|Staphylococcys | lmrS | multidrug |
| NC_009632_5316779_p01 |  |  |
| gb\|AAA26652.1\|ARO:3002835\|lnuA | lnuA | MLS |
| gb\|AEA37904.1\|ARO:3003112\|lsaC | lsaC | multidrug |
| gb\|AAV85981.1\|ARO:3000533\|macA | macA | MLS |
| NC_002695_1_917702_p01 |  |  |
| gb\|AAV85982.1\|ARO:3000535\|macB | macB | MLS |
| gb\|BAA15221.2\|ARO:3000263\|marA | marA | multidrug |
| gb\|BAE06008.1\|ARO:3003699\|mexQ | mdsB | multidrug |
| gb\|AAL19305.1\|ARO:3000790\|mdsB |  |  |
| gb\|AAC75135.2\|ARO:3000792\|mdtA | mdtA | aminocoumarin |
| gb\|AAC75136.1\|ARO:3000793\|mdtB | mdtB | aminocoumarin |
| gb\|AAC75137.1\|ARO:3000794\|mdtC | mdtC | aminocoumarin |
| gb\|BAE77781.1\|ARO:3000795\|mdtE | mdtE | multidrug |
| gb\|AAC76539.1\|ARO:3000796\|mdtF | mdtF | multidrug |
| gb\|ABV18113.1\|ARO:3001329\|mdtG | mdtG | fosfomycin |
| gb\|AAC74149.2\|ARO:3001216\|mdtH | mdtH | fluoroquinolone |
| gb\|AAC77293.1\|ARO:3001214\|mdtM | mdtM | multidrug |
| gb\|BAE78084.1\|ARO:3003548\|mdtN | mdtN | aminoglycoside |
| gb\|BAE78083.1\|ARO:3003549\|mdtO | mdtO | aminoglycoside |
| gb\|BAE78082.1\|ARO:3003550\|mdtP | mdtP | aminoglycoside |
| (Bla)mecA:AB221124:91-2097:2007 | mecA | beta-lactamase |
| gb\|AGC51118.1\|ARO:3000617\|mecA |  |  |
| gb\|BAB41258.1\|ARO:3000124\|mecI | mecI | beta-lactamase |
| gb\|ABQ47844.1\|ARO:3000215\|mecR1 | mecR1 | beta-lactamase |
| NC_007793_3913613_p01 |  |  |
| gb\|AAF74725.1\|ARO:3004659\|Mef(En2) | Mef(En2) | MLS |
| (MLS)Mef(En2):NG_047980:101-1306:1206 |  |  |
| gb\|ACJ63262.1\|ARO:3003107\|mefB | mefB | MLS |
| gb\|AAK99775.1\|ARO:3000614\|mefE | mefE | MLS |
| gb\|AAL73129.1\|ARO:3000616\|mel | mel | multidrug |
| (MLS)mef(A):U70055:314-1531:1218 |  |  |
| gb\|AAU95768.1\|ARO:3000026\|mepA | mepA | multidrug |
| gb\|BAB56495.1\|ARO:3000746\|mepR | mepR | multidrug |
| gb\|AAA74437.1\|ARO:3000378\|MexB | mexB | multidrug |
| gb\|AAG05882.1\|ARO:3000804\|MexF |  |  |
| gb\|AAG07064.1\|ARO:3003693\|mexK | mexK | multidrug |
| gb\|BAB41874.1\|ARO:3000815\|mgrA | mgrA | multidrug |
| gb\|AAG57600.1\|ARO:3000318\|mphB | mphB | MLS |
| gb\|BAA34540.1\|ARO:3000319\|mphC | mphC | MLS |
| (MLS)mph(C):AF167161:5665-6564:900 |  |  |
| gi\|57866874\|ref\|YP_188509_1\| | MprF | peptide |
| gb\|AAC74000.1\|ARO:3003950\|msbA | msbA | nitroimidazole |
| (MLS)msr(A):AY591760:274-1740:1467 | msrA | multidrug |
| gb\|CAA36304.1\|ARO:3000251\|msrA |  |  |
| (MLS)msr(D):AF274302:2462-3925:1464 | msrD | MLS |
| gb\|CCP46065.1\|ARO:3000816\|mtrA | mtrA | multidrug |
| gb\|AAF42063.1\|ARO:3000810\|mtrC | mtrC | multidrug |
| gb\|AAF42062.1\|ARO:3000811\|mtrD | mtrD | multidrug |
| gb\|CAA64891.1\|ARO:3000812\|mtrE | mtrE | multidrug |
| gb\|ACF30254.1\|ARO:3000817\|mtrR | mtrR | multidrug |
| gb\|CAA53189\|ARO:3000521\|mupA | mupA | mupirocin |
| (Ntmdz)NimB_Nitroimidazole_Gene:X71443:794-1288:495 | NimB | Nitroimidazole |
| (Flq)norA:D90119:478-1644:1167 | norA | multidrug |
| gb\|AAS68233.1\|ARO:3000391\|norA |  |  |
| NC_002952_2859165_p01 |  |  |
| NC_007622_3793623_p01 |  |  |
| (AGly)npmA:AB261016:3069-3728:660 | npmA | aminoglycoside |
| gb\|CAA09666.1\|ARO:3004122\|Klebsiella | OmpK37 | beta-lactamase |
| (Flq)OqxA:EU370913:46652-47827:1176 | OqxA | multidrug |
| (Flq)OqxBgb:EU370913:47851-51003:3153 | OqxBgb | multidrug |
| gb\|AAK76137.1\|ARO:3000024\|patA | patA | fluoroquinolone |
| gb\|AAK76136.1\|ARO:3000025\|patB | patb | fluoroquinolone |
| (Bla)PBP1a:JN645776:1-2160:2160 | Pbp1A | beta-lactamase |
| (Bla)PBP1b:AF101781:1-2466:2466 | PBP1b | beta-lactamase |
| (Bla)penA:AB511945:1298-3049:1762 | Pena | beta-lactamase |
| (Bla)Penicillin_Binding_Protein_Ecoli:CP002291:664439-666340:1902 | Penicillin_Binding_Protein | beta-lactamase |
| gb\|BAG33043.1\|ARO:3003920\|pgpB | pgpB | peptide |
| gb\|AAK99679.1\|ARO:3000822\|pmrA | pmrA | fluoroquinolone |
| gb\|AAC75314.1\|ARO:3003578\|pmrF | pmrF | peptide |
| gb\|BAJ09383.1\|ARO:3003046\|qacA | qacA | fluoroquinolone |
| gb\|AAQ10694.1\|ARO:3003047\|qacB | qacB | fluoroquinolone |
| gb\|AFK13828.1\|ARO:3000823\|ramA | ramA | multidrug |
| gb\|AJD73064.1\|ARO:3001301\|RlmA(II) | RlmAII | MLS |
| gi\|387965694\|gb\|AFK13827_1\| | robA | multidrug |
| gi\|22127597\|ref\|NP_671020_1\| |  |  |
| (AGly)sat-2A:X51546:518-1042:525 | SAT-1 | aminoglycoside |
| (AGly)sat4A:X92945:38870-39412:543 | SAT-4 | aminoglycoside |
| gb\|AAB53445.1\|ARO:3002897\|SAT-4 |  | aminoglycoside |
| NC_002758_1121879_p01 | SAV1866 | multidrug |
| CP000034_1_gene4504_p01 | soxR |  |
| NC_012469_1_7686946_p01 | SPT_0145 | MLS |
| NC_012469_1_7686878_p01 | SPT_1414 | MLS |
| NC_012469_1_7685735_p01 | SPT_1593 | MLS |
| gb\|AEJ33969.1\|ARO:3000410\|sul1 | sul1 | sulfonamide |
| gb\|AAL59753.1\|ARO:3000412\|sul2 | sul2 | sulfonamide |
| gb\|BAC67143.1\|ARO:3000166\|tet(B) | tet(B) | tetracycline |
| gb\|AAB28795.1\|ARO:3000178\|tet(K) | tet(K) | tetracycline |
| gb\|ACH87088.1\|ARO:3000196\|tet32 | tet32 | tetracycline |
| (Tet)tet(32):DQ647324:181-2100:1920 |  |  |
| gb\|AAN28721.1\|ARO:3002871\|tet37 | tet37 | tetracycline |
| AY825285_gene_p01 | tet38 | tetracycline |
| gb\|CAM12479.1\|ARO:3000567\|tet(40) | tet40 | tetracycline |
| (Tet)tet(40):AM419751:14211-15431:1221 |  |  |
| FN594949_1_gene24_p01 | tet44 | tetracycline |
| gb\|CBH51823.1\|ARO:3000556\|tet44 |  |  |
| AB084246_gene_p01 | tetA | tetracycline |
| AF534183_gene_p01 |  |  |
| CP001485_1_gene2821_p01 |  |  |
| gb\|NP_862226.1\|ARO:3004639\|Corynebacterium |  |  |
| NC_010410_6002612_p01 |  |  |
| X75761_gene_p01 |  |  |
| gb\|AET10444.1\|ARO:3004032\|tetA(46) | tetA(46) | tetracycline |
| gb\|ANZ79240.1\|ARO:3004035\|tetA(60) | tetA(60) | tetracycline |
| NC_010558_1_6275971_p01 | tetA(B) | tetracycline |
| gb\|AET10445.1\|ARO:3004033\|tetB(46) | tetB(46) | tetracycline |
| gb\|ANZ79241.1\|ARO:3004036\|tetB(60) | tetB(60) | tetracycline |
| (Tet)tetG:AF133140:757-1932:1176 | tetG | tetracycline |
| (Tet)tetK:M16217:305-1684:1380 | tetK | tetracycline |
| gb\|AAA22851.1\|ARO:3000179\|tet(L) | tetL | tetracycline |
| (Tet)tetL:FN435329:1-1377:1377 |  |  |
| gb\|CAQ49384.1\|ARO:3000186\|tetM | tetM | tetracycline |
| (Tet)tetM:DQ534550:1451-3370:1920 |  |  |
| AJ222769_gene_p01 | TetO | tetracycline |
| AJ295238_gene_p01 |  |  |
| AY485126_gene_p01 |  |  |
| DQ294299_gene_p01 |  |  |
| gb\|AAA23033.2\|ARO:3000190\|tetO |  |  |
| M85225_gene_p01 |  |  |
| NC_009782_5559075_p01 |  |  |
| (Tet)tetO:M18896:207-2126:1920 |  |  |
| X04388_gene_p01 |  |  |
| gb\|AAA20116.1\|ARO:3000180\|tetA(P) | tetP | tetracycline |
| gb\|CAA79727.1\|ARO:3000191\|tetQ | tetQ | tetracycline |
| (Tet)tetQ:Z21523:362-2287:1926 |  |  |
| (Tet)tetR:HF545434:53576-54226:651 | tetR | tetracycline |
| (Tet)tetR(G):S52438:113-745:633 | tetR(G) | tetracycline |
| gb\|CAA10975.1\|ARO:3000194\|tetW | tetW | tetracycline |
| (Tet)tetW:AJ222769:3687-5606:1920 |  |  |
| gb\|AMP42147.1\|ARO:3004442\|tet(W/N/W) | tetWNW | tetracycline |
| gb\|AAA27471.1\|ARO:3000205\|tetX | tetX | tetracycline |
| (Tet)tetX:M37699:586-1752:1167 |  |  |
| gi\|326634054\|pdb\|2XDO\|A | tetx2 | tetracycline |
| gb\|QBQ85438.1\|ARO:3004719\|Tet(X3) | tetX3 | tetracycline |
| gb\|QBQ69719.1\|ARO:3004720\|Tet(X4) | tetX4 | tetracycline |
| gb\|ACN32294.1\|ARO:3000237\|tolC | tolC | multidrug |
| NC_002695_1_916248_p01 |  |  |
| gb\|AAC75089.1\|ARO:3003577\|ugd | ugd | peptide |
| (Gly)vanA-G:AY271782:157-606:450 | vanAG | glycopeptide |
| gb\|ABA71731.1\|ARO:3002909\|vanG | vanG | glycopeptide |
| AF253562_2_orf0_gene_p01 | vanRG | glycopeptide |
| DQ212986_1_gene11_p01 | vanTG | glycopeptide |
| gb\|AAO82019.1\|ARO:3004254\|vanVB | vanVB | glycopeptide |
| AF253562_2_orf3_gene_p01 | vanW | glycopeptide |
| DQ212986_1_gene7_p01 | vanWG | glycopeptide |
| gb\|ABA71732.1\|ARO:3003069\|vanXYG | vanXYG | glycopeptide |
| gb\|AAA26684.1\|ARO:3002829\|vgaA | vgaA | multidrug |
| gb\|ABH10964.1\|ARO:3002830\|vgaALC | vgaALC | multidrug |
| gb\|AAC75271.1\|ARO:3003952\|yojI | yojl | peptide |

**Supplementary Table 6. Study of the ARGs richness and abundance.** For the different studied samples, a different number of ARGs were found (richness). Also, the most abundant ARG in each case is pointed and the drug class they confer resistance against.

| **Body part** | **Nº different ARGs** | **More abundant ARG** | **Resistence to** |
| --- | --- | --- | --- |
| Vagina | 37 | *tetM* | Tetracyclin |
| Gut | 155 | *tetQ* | Tetracyclin |
| Skin | 63 | *mprF* | Peptide antibiotic |
| Nares | 89 |  |  |
| Oral cavity | 116 | *patB* | Fluoroquinolone |
|  |  |  |  |
|  | No. of different ARGs in all the body | 235 |  |

**Supplementary Table 7.** **ARGs from Human Microbiome Project dataset in pristine environments.** 9 ARG were found in pristine environments (IMG genome ID) in contigs (contig ID) with at least 4 proteins, an amino acid identity of 90% and a bit-score of 70 compared with the HMP antibiotic resistance genes. Those contigs belonging to taxons that could not be associated with a specific environment were discarded. The table includes the taxonomic annotation (annotation), the ARG, body part where it was also found and their classification: Contamination (e.g. laboratory bacterial contaminants) or Autochthonous (autochthonous bacteria with an ARG previously found in HMP dataset).

| **Pristine Environment** | **IMG Genome ID** | **Contig ID** | **Annotation** | **ARG** | **Body part** | **Classified as** |
| --- | --- | --- | --- | --- | --- | --- |
| Submarine volcano | 3300000098 | ZETA_00024 | *Escherichia coli* | APH(3')-I | Feces | Contamination |
|  |  |  |  | APH3-Ia | Oral | Contamination |
| Submarine volcano | 3300000272 | LM34_100081 | *Escherichia coli* | APH3-Ia | Oral | Contamination |
| Submarine volcano | 3300000327 | ES34_100970 | *Escherichia coli* | APH3-Ia | Oral | Contamination |
| Submarine volcano | 3300000327 | ES34_1009954 | *Rhodobacteraceae* | CAT | Skin | Autochthonous |
| Hot springs | 2010170002 | BISONQ_C849 | *Enterobacter sp.* | CRP | Feces | Contamination |
| Hot springs | 2010170002 | BISONQ_C10353 | *Enterobacter sp.* | rob | Feces | Contamination |
| Hot springs | 3300005799 | Ga0079638_101036 | *Escherichia coli* | APH(3')-IIa | Oral | Contamination |
|  |  |  |  | APH(3')-IIa | Oral | Contamination |

**References**

Arango-Argoty, G., Garner, E., Pruden, A., Heath, L. S., Vikesland, P., and Zhang, L. (2018). DeepARG: a deep learning approach for predicting antibiotic resistance genes from metagenomic data. *Microbiome* 6, 23. doi:10.1186/s40168-018-0401-z.

Avalos, E., Catanzaro, D., Catanzaro, A., Ganiats, T., Brodine, S., Alcaraz, J., et al. (2015). Frequency and Geographic Distribution of gyrA and gyrB Mutations Associated with Fluoroquinolone Resistance in Clinical Mycobacterium Tuberculosis Isolates: A Systematic Review. *PLoS One* 10, e0120470. doi:10.1371/journal.pone.0120470.

Chen, I. M. A., Chu, K., Palaniappan, K., Ratner, A., Huang, J., Huntemann, M., et al. (2021). The IMG/M data management and analysis system v.6.0: New tools and advanced capabilities. *Nucleic Acids Res.* 49, D751–D763. doi:10.1093/nar/gkaa939.

Gibson, M. K., Forsberg, K. J., and Dantas, G. (2015). Improved annotation of antibiotic resistance determinants reveals microbial resistomes cluster by ecology. *ISME J.* 9, 207–16. doi:10.1038/ismej.2014.106.

Gupta, S. K., Padmanabhan, B. R., Diene, S. M., Lopez-Rojas, R., Kempf, M., Landraud, L., et al. (2014). ARG-ANNOT, a new bioinformatic tool to discover antibiotic resistance genes in bacterial genomes. *Antimicrob. Agents Chemother.* 58, 212–220. doi:10.1128/AAC.01310-13.

Huttenhower, C., Gevers, D., Knight, R., Abubucker, S., Badger, J. H., Chinwalla, A. T., et al. (2012). Structure, function and diversity of the healthy human microbiome. *Nature* 486, 207–214. doi:10.1038/nature11234.

Jia, B., Raphenya, A. R., Alcock, B., Waglechner, N., Guo, P., Tsang, K. K., et al. (2017). CARD 2017: expansion and model-centric curation of the comprehensive antibiotic resistance database. *Nucleic Acids Res.* 45, D566–D573. doi:10.1093/nar/gkw1004.

Markowitz, V. M., Chen, I.-M. A., Palaniappan, K., Chu, K., Szeto, E., Grechkin, Y., et al. (2012). IMG: the integrated microbial genomes database and comparative analysis system. *Nucleic Acids Res.* 40, D115–D122. doi:10.1093/nar/gkr1044.
